# Synergistic anion-cation descriptor for bidirectional electrocatalyst in Li-CO_2_ battery

**DOI:** 10.1126/sciadv.aee9103

**Published:** 2026-06-19

**Authors:** Xingwu Zhai, Yuchun Liu, Mi Luo, Tianchen Wei, Hang Wang, Jing Zhang, Leyi Su, Zhaodi Fan, Zhixin Sun, Zhuohui Zhang, Liang Wu, Hongjun Zhang, Bangjiao Ye, Min Zhou

**Affiliations:** ^1^ Department of Radiology, The First Affiliated Hospital of USTC, Hefei National Research Center for Physical Sciences at the Microscale, School of Chemistry and Materials Science, University of Science and Technology of China, Hefei, Anhui 230026, China.; ^2^ State Key Laboratory of Particle Detection and Electronics, Department of Modern Physics, University of Science and Technology of China, Hefei 230026, China.

## Abstract

The slow kinetics of lithium carbonate (Li_2_CO_3_) nucleation/decomposition hinder voltage gap minimization in lithium-carbon dioxide (Li-CO_2_) batteries. Although symmetry-broken cation motifs can enhance reactivity, designing optimal catalysts remains challenging. Moving beyond cation-centric views, we recognize anions as active participants that regulate charge and stabilize intermediates, yet their degradation worsens the activity-stability trade-off. To address this, we develop a dual Φ descriptor quantifying anion-cation orbital coupling and reconstruction energy. It establishes a volcano correlation with the voltage gap in metal sulfides, showing that symmetry-broken units optimally balance binding and stability. Guided by this, we synthesize oriented WS_2_ rich in C_4v_ configurations, achieving a record-low gap of 0.76 volts and superior cycling (>1268 hours) among dichalcogenides. This work shifts the paradigm from cation-only tuning to synergistic anion-cation design, repositioning anions as co-catalytic architects. By linking orbital insights to performance, we provide a universal descriptor for developing efficient, stable Li-CO_2_ batteries.

## Teaser

A dual Φ that combines electronic and structural effects guides the design of bidirectional catalysts for Li-CO_2_ batteries.

## INTRODUCTION

**Fig. 1.**
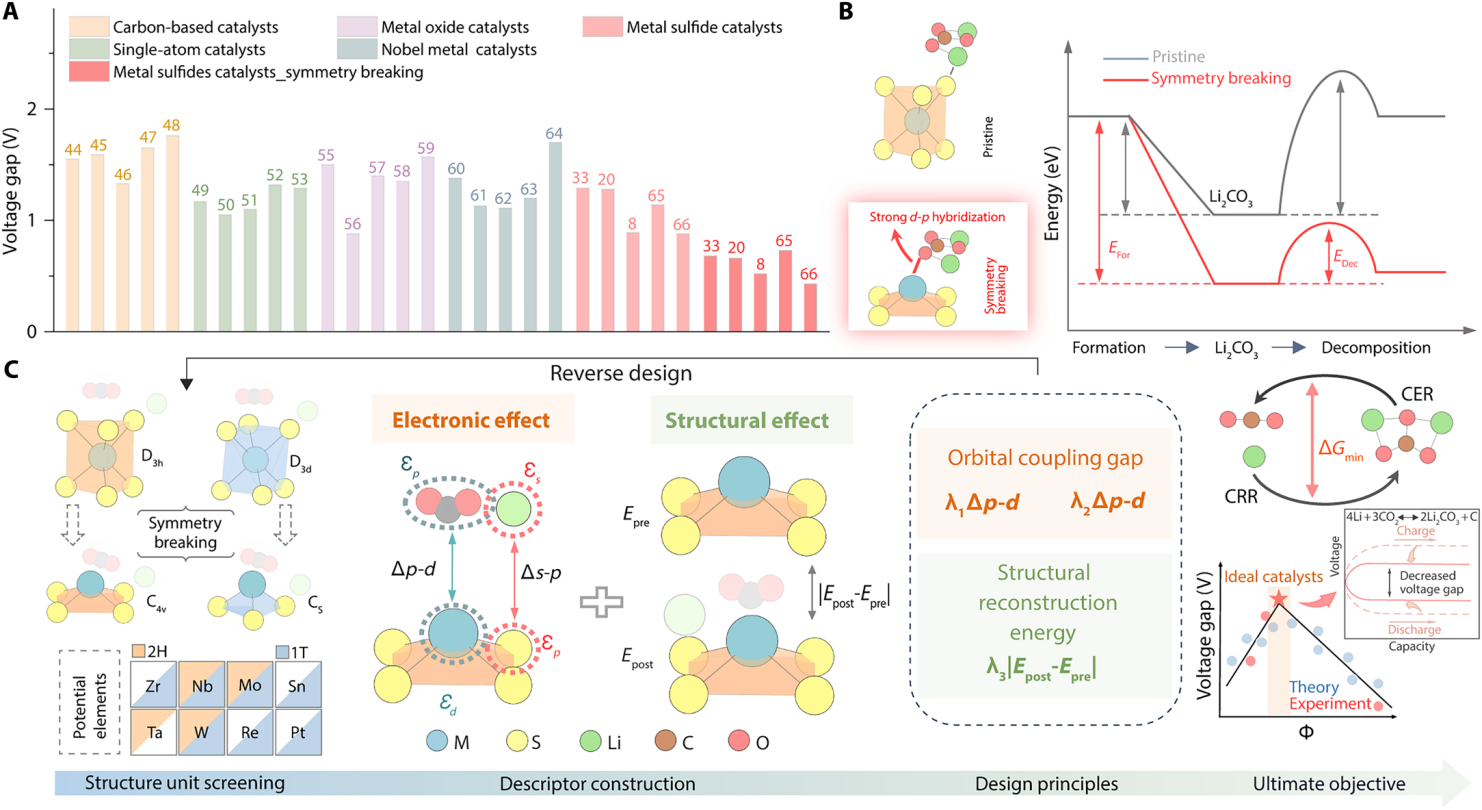
Framework for designing electronic and structural descriptors. (**A**) Performance comparison of reported catalysts in tables S3 and S4 (*8*, *20*, *33*, *44*–*66*). (**B**) Facilitated Li_2_CO_3_ nucleation and decomposition through symmetry-breaking engineering. (**C**) Revealing the relationship of the quantitative descriptor with voltage gap.

To promote the sluggish kinetics of the CO_2_ reduction/evolution reaction (CO_2_RR/CO_2_ER) in rechargeable Li-CO_2_ batteries (*1*–*10*), the exploration of efficient bidirectional catalysts with minimal voltage gap poses critical challenges. In general, an efficient bidirectional catalyst should facilitate the formation and decomposition of solid products like Li_2_CO_3_ (*11*–*14*). In the context of bifunctional catalysis for Li-CO_2_ batteries, transition metal dichalcogenides (TMDs) exhibit exceptional performance characterized by ultralow voltage gaps (Fig. 1A). The slow kinetics of CO_2_RR/CO_2_ER on high-symmetry cation-centered structures (e.g., D_3d_ and D_3h_) are attributed to insufficient *d-p* hybridization between metal *d*-orbitals and Li_2_CO_3_ *p*-orbitals, induced by negative coordination shielding. Introducing symmetry-breaking motifs enhances this orbital interaction, promotes the reversible nucleation/decomposition of Li_2_CO_3_, and lowers the activation energy, ultimately leading to superior catalytic activity with a minimal voltage gap (Fig. 1B). The lack of robust theoretical guidance from frameworks and the development of bidirectional catalysts still rely heavily on the trial-and-error method prevalent in laboratories, which greatly impedes the design of bidirectional catalysts. Therefore, it is imperative to establish a universal descriptor to quantitatively describe the relationship between structure and activity in Li-CO_2_ batteries. Conventionally, electronic parameters, such as the valence electron, electronegativity, and energy level of the *d* orbital state (*7*, *15*), have been used to predict the electrochemical performance batteries by modifying the valence electron states of active metal sites and optimizing the adsorption/desorption behavior of intermediates. These parameters may not provide definitive selection criteria because they have predominantly targeted metal cations while neglecting the crucial contributions of nonmetal anions.

Within a bifunctional catalyst, anions play versatile roles that transcend their conventional perception as static charge compensators. First, anions actively modulate the electronic structure of adjacent cations through orbital hybridization and charge transfer. Anionic configurations can proactively redistribute charge density at cationic sites. Second, anions can be directly involved in reactive intermediate stabilization via preferred bonding with Li-electrophilic atoms. Third, the above-mentioned dual functionalities are critically undermined by progressive anionic degradation during cycling, including electrochemical dissolution, structural collapse, parasitic reactions, etc. Catalytic activity is diminished through the depletion of active sites for intermediate stabilization and thereby accelerates phase collapse and interfacial side reactions, leading to an irreversible overpotential increase. Anionic spatial degradation during cycling results in the unresolved activity-stability trade-off. Therefore, electronic parameters combined with cations and anions may contribute more to capturing the intrinsic essence of CO_2_RR/CO_2_ER activities in Li-CO_2_ batteries. A descriptor that integrates contributions of nonmetal anions and pre- and postreaction states for accurate predictions of complex systems remains unexplored in Li-CO_2_ batteries.

Previous efforts in descriptor development have primarily focused on relatively simple systems, such as single-atom–modified MXenes for predicting charge voltages or a limited set of noble metal–based materials for voltage gaps (*7*, *15*). Although layered TMDs with intrinsic edge symmetry-breaking motifs and well-defined anion-cation configurations exhibit excellent performance in Li-CO_2_ batteries, the fundamental complexity and variability of these configurations pose a substantial challenge for formulating specific descriptors (*16*–*20*). Here, we establish a quantitative descriptor to accurately predict the voltage gaps for Li-CO_2_ batteries, considering the synergistic anion-cation dual electron coupling and the structural durability upon pre- and postreaction (Fig. 1C). Specifically, we first predicted the theoretical voltage gap using density functional theory (DFT) calculations, followed by Pearson correlation analysis to identify the most relevant feature values correlated with the voltage gap. By analyzing feature values, a descriptor (Φ) that encapsulates both the orbital coupling gap and structural reconstruction energy was constructed to analyze and predict the voltage gap in Li-CO_2_ batteries. Our findings indicate that the voltage gap is governed by the strength and adaptability of the interaction between LiCO_2_ intermediates and the catalyst. A bidirectional catalyst that exhibits the smallest voltage gap should have good LiCO_2_ intermediates interfacial conversion kinetics and a relatively stable structure. We have extended the dual descriptor Φ to other layered TMDs, demonstrating its potential as a universal descriptor for the design of efficient catalysts in Li-CO_2_ batteries. The direct visualization of Li_2_CO_3_ nucleation/decomposition sites offers conclusive evidence that C_4v_ low-symmetry motifs facilitate these reactions. Last, the accuracy of Φ is verified by experimental measurements of charge-discharge voltages and voltage gaps. As a result, the predicted WS_2_ with rich C_4v_ configurations demonstrates the smallest voltage gap of 0.76 V at 20 μA cm^−2^ and the longest cycling stability of 1268 hours.

## RESULTS

Given the synergistic effects of cations and anions in symmetry-breaking structural units, layered TMDs featuring intrinsic symmetry breaking serve as ideal models for elucidating reaction mechanisms. The basal planes (D_3h_ unit), which constitute most of the surface area in semiconducting TMDs (figs. S1 and S2), exhibit poor intrinsic reactivity relative to the symmetry-breaking edge sites (*21*, *22*). Typically, layered TMDs exhibit armchair and zigzag edges (C_4v_ symmetry) (*23*), with the zigzag edge (labeled as zig-C_4v_) being energetically favorable (*24*, *25*). Taking the rich zig-C_4v_ units of MoS_2_ and WS_2_ as examples, we show the convergence tests in figs. S3 and S4 and table S1. The spin density distribution (up-down) calculations reveal that the MoS_2_/WS_2_ armchair edges (arm-C_4v_) do not generate magnetic moments, while the magnetic moments of zig-C_4v_ unit originate primarily from unpaired electrons at the edge Mo/W and S atoms, with negligible contribution from inner atoms (fig. S5) (*26*, *27*). It should be noted that the observed spin polarization at zig-C_4v_ edge serves only as a structural probe for unsaturated coordination, rather than an indicator of spin-dependent reactivity. For the density of states (DOS) of D_3h_ unit of the basal plane and zig-C_4v_ unit models, the D_3h_ unit exhibits semiconductor characteristics, whereas the zig-C_4v_ unit model demonstrates metallic behavior. This distinction is attributed to the electronic states of the metal atoms and S atoms in the zig-C_4v_ unit at the Fermi level (fig. S6). This is further visualized by the partial charge density calculations (fig. S7). Moreover, by comparing the adsorption of Li, CO_2_, and Li_2_CO_3_ on the D_3h_ and zig-C_4v_ units, it is found that the zig-C_4v_ unit can promote the adsorption of Li, CO_2_, and Li_2_CO_3_ intermediates (fig. S8). The charge density difference analysis (fig. S9) reveals pronounced interfacial charge redistribution, confirming the strong electronic coupling that facilitates Li_2_CO_3_ decomposition on the zig-C_4v_ unit. While spin density calculations offer a convenient visualization tool for probing edge-localized symmetry-breaking structures, the catalytic activity of zig-C_4v_ edges is fundamentally governed by their thermodynamic stability, metallic electronic character, enhanced intermediate adsorption, and reduced reaction barriers. These characteristics are intrinsic properties of the symmetry-breaking coordination environment and are entirely independent of any spin-selective reaction mechanisms. Accordingly, the spin polarization observed in our calculations serves solely as a structural probe to verify the presence of symmetry-breaking configurations, rather than as a descriptor of spin-dependent reactivity. In subsequent calculations, the zig-C_4v_ unit will be consistently designated as the active site.

**Fig. 2.**
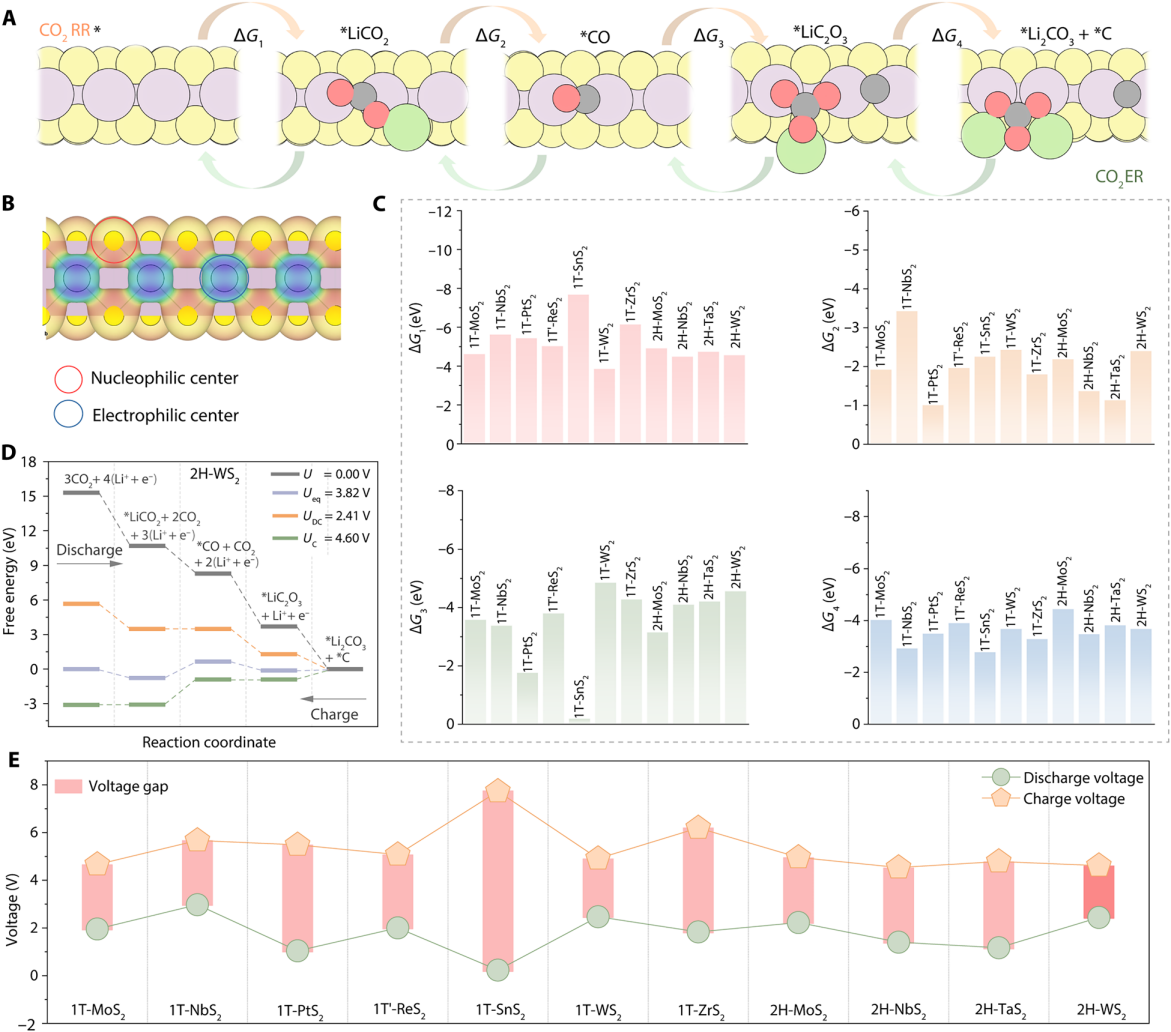
DFT calculations of voltage gap for all sulfide candidates. (**A**) The reaction mechanism of the CO_2_RR/CO_2_ER process. (**B**) Surface electrostatic potential diagram of the 2H-WS_2_ zigzag edge. (**C**) Gibbs free energy change (Δ*G*) for each elementary reaction step along the four-electron reaction pathway. (**D**) Calculated discharge/charge and equilibrium potential for 2H-WS_2_. (**E**) Discharge/charge voltage and voltage gaps for 11 selected layered TMDs as electrocatalysts for Li-CO_2_ batteries.

As shown in Fig. 2A, the process of the Li-CO_2_ battery involves a four-electron conversion reaction (*15*). During the discharging process (CO_2_RR process), Li and CO_2_ act as key reaction intermediates and participate in the first step of the reaction. To initiate the catalytic cycle, the catalyst must have active sites capable of binding and activating Li and CO_2_. The surface electrostatic potential diagrams (Fig. 2B) of the metal-terminated zig-C_4v_ units indicate that the nucleophilic S sites and electrophilic metal sites in the zig-C_4v_ units serve as adsorption sites of Li and CO_2_, respectively, compared to the D_3h_ unit (fig. S10). Further adsorption energy calculations (fig. S11) also confirm this conclusion. Li tends to bind more readily with S, while CO_2_ molecules prefer to bind with metal sites. In terms of the charging process (CO_2_ER process), the decomposition of the most common discharge product (Li_2_CO_3_) is critical (*7*). The comparison of the adsorption energies of Li_2_CO_3_ reveals that its adsorption at zig-C_4v_ units is stronger than on the D_3h_ units. Li_2_CO_3_ is adsorbed on the D_3h_ units through Li-S bonds, while at zig-C_4v_ units, it is adsorbed through both Li-S bonds and Mo/W-O bonds (fig. S12). The incorporation of Mo/W-O bonds can further weaken the Li-O bonds and promote the decomposition of Li_2_CO_3_, which is evidenced by the stretched Li-O length and the more positive integrated crystal occupation Hamiltonian population values (figs. S13 and S14). In addition, the decomposition energy barrier of Li_2_CO_3_ using the climbing-image nudged elastic band (CI-NEB) method further confirms the origin of high catalytic activity of zig-C_4v_ units from a kinetic perspective (figs. S15 and S16). These results further support that the symmetry-breaking zig-C_4v_ units of layered TMDs provide highly active sites for Li-CO_2_ battery.

To obtain the theoretical voltage gap, we calculated the Gibbs free energy change (Δ*G*) of four elementary steps for selected layered TMDs (Fig. 2C). The results demonstrate that for almost all systems, the formation of the first Li_2_CO_3_ molecule is the rate-determining step (RDS) of the discharging process, whereas the RDS for the charging process is the decomposition of LiCO_2_ intermediate. Figure 2D depicts the Gibbs free energy profiles of 2H-WS_2_ during discharge/charge for Li-CO_2_ batteries (corresponding optimized structures and information of several typical semiconductor phases of layered TMDs are as seen in figs. S17 and S18). The results indicate that the elementary four-electron transfer steps during discharge are spontaneous (Δ*G* < 0) when *U* = 0 V. During the CO_2_RR/CO_2_ER process, 2H-WS_2_ exhibits a higher discharge voltage (2.41 V) and a lower charge voltage (4.60 V) compared to other layered TMDs, indicating a higher reversibility in Li-CO_2_ batteries. Figure 2E summarizes the discharge/charge voltage and voltage gap for 11 selected layered TMDs. Among the candidates, 2H-WS_2_ exhibits the smallest voltage gap of 2.19 V, suggesting its outstanding performance in Li-CO_2_ batteries.

**Fig. 3.**
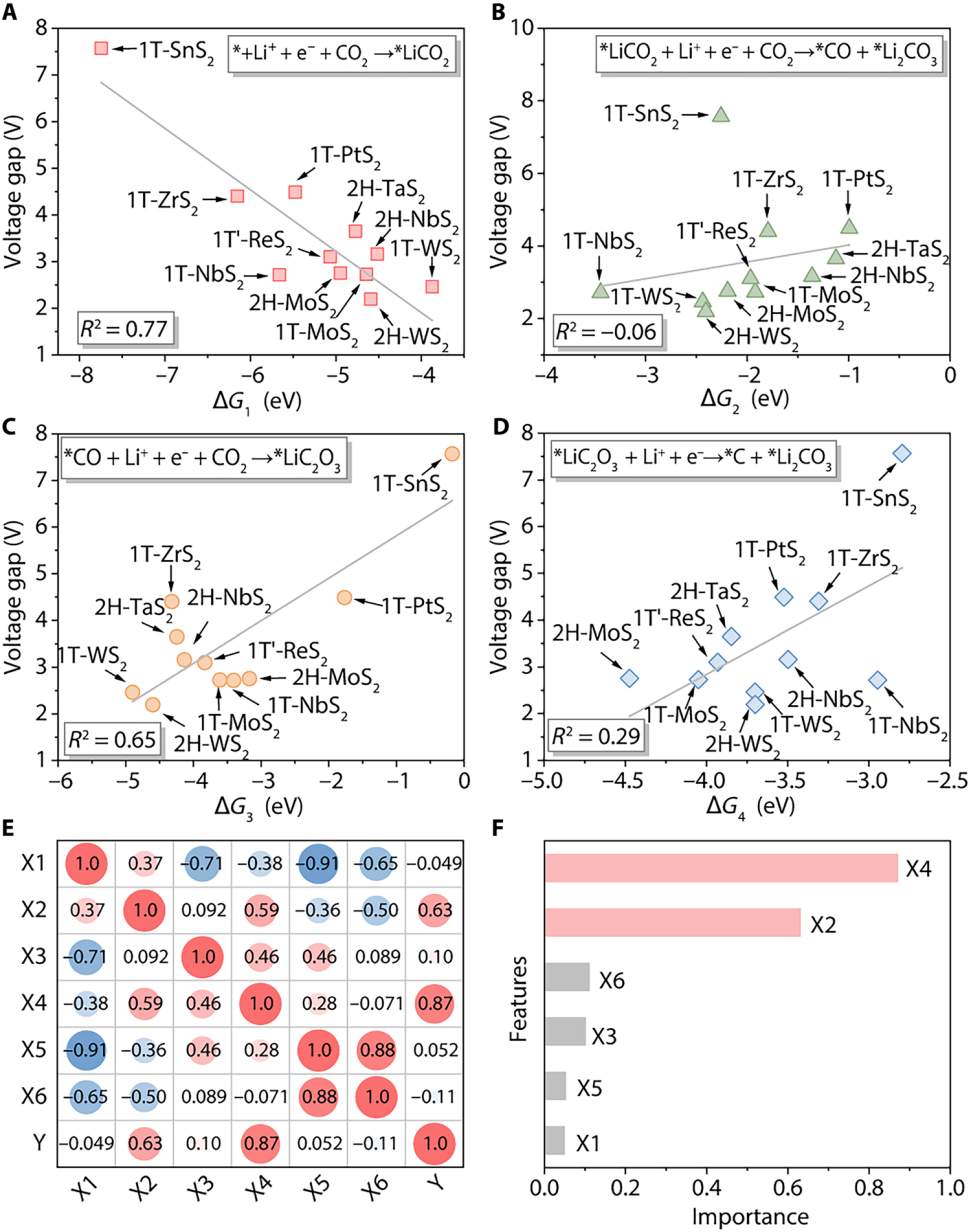
Extraction of critical features for descriptor construction. Voltage gap versus (**A**) Δ*G*_1_, (**B**) Δ*G*_2_, (**C**) Δ*G*_3_, and (**D**) Δ*G*_4_ for all layered TMDs in Li-CO_2_ batteries. (**E**) Heatmap of Pearson correlation coefficients between the voltage gap and six key features. X1, *d*-band center of the metal; X2, *p*-band center of S; X3, electronegativity of metal; X4, M-S bond energy; X5, the first ionization energy of metal; X6, the atomic number of metals; Y, the voltage gap. (**F**) Corresponding importance rankings of the six parameters.

During the CO_2_RR/CO_2_ER in Li-CO_2_ batteries, the energy barriers of the RDS are primarily determined by the adsorption free energies of the reaction intermediates *LiCO_2_, *CO, *LiC_2_O_3_, and *Li_2_CO_3_ + *C. In other words, the differences in the voltage gap for the reactions are attributed to the distinct binding strengths of the adsorbed intermediates on different catalysts. Therefore, establishing the relationship between the changes in adsorption free energy of the intermediates and the voltage gap is crucial for exploring the smallest voltage gap. Figure 3 (A to D) plots the relationship between voltage gap and the Gibbs free energy change (Δ*G*_1_, Δ*G*_2_, Δ*G*_3_, and Δ*G*_4_) of the four elementary steps, which involve four different reaction intermediates. On the basis of the relationships between the voltage gap and the Δ*G* of each step as fitted by linear regression, the changes in adsorption free energy of the LiCO_2_ intermediates (Δ*G*_1_) are found to be most strongly correlated with the voltage gap (*R*^2^ = 0.77), suggesting that the adsorption strengths of the LiCO_2_ intermediates may be the primary factor determining the voltage gap (*28*). Thus, we keep on focusing on the adsorption of LiCO_2_ intermediates in depth in the following sections.

To identify the key intrinsic factors governing Δ*G*_1_ and its relationship with the voltage gap, we calculated Pearson correlation coefficients among relevant parameters, revealing several dominant factors: the *d*-band center (ε*_d_*) of metal atoms, the *p*-band center (ε*_p_*) of sulfur anions in layered TMDs, metal electronegativity, M-S bond energy, and the first ionization energy and atomic number of the metal (Fig. 3E). Feature importance analysis demonstrates that the S ε*_p_* exerts a more pronounced influence on the voltage gap than the metal ε*_d_*, challenging the conventional emphasis on transition metal cation ε*_d_* in catalytic activity (Fig. 3F) (*29*–*31*). Correlation analysis between ε*_d_*/ε*_p_* and voltage gaps further highlights the crucial role of nonmetal anions in the catalytic process (*32*), indicating their notable impact on catalytic activity. As shown in fig. S19, although the ε*_p_* of S anions exhibits a stronger correlation with the voltage gap compared to the ε*_d_* of transition metal cations, its relatively low coefficient of determination (*R*^2^ = 0.32) prevents it from serving as a reliable standalone descriptor. These results demonstrate that the intrinsic electronic properties of catalysts, as represented by traditional ε*_d_*/ε*_p_* parameters, are insufficient to fully explain the voltage gap regulation by layered TMDs. Instead, electronic coupling between key intermediates (e.g., LiCO_2_) and catalyst surfaces emerges as a critical factor in determining voltage gaps. Moreover, the observed correlation between M-S bond energy and the voltage gap suggests that structural characteristics of active sites also contribute substantially to voltage gap modulation. Consequently, it is warranted to develop a descriptor that considers both electronic coupling and structural stability.

**Fig. 4.**
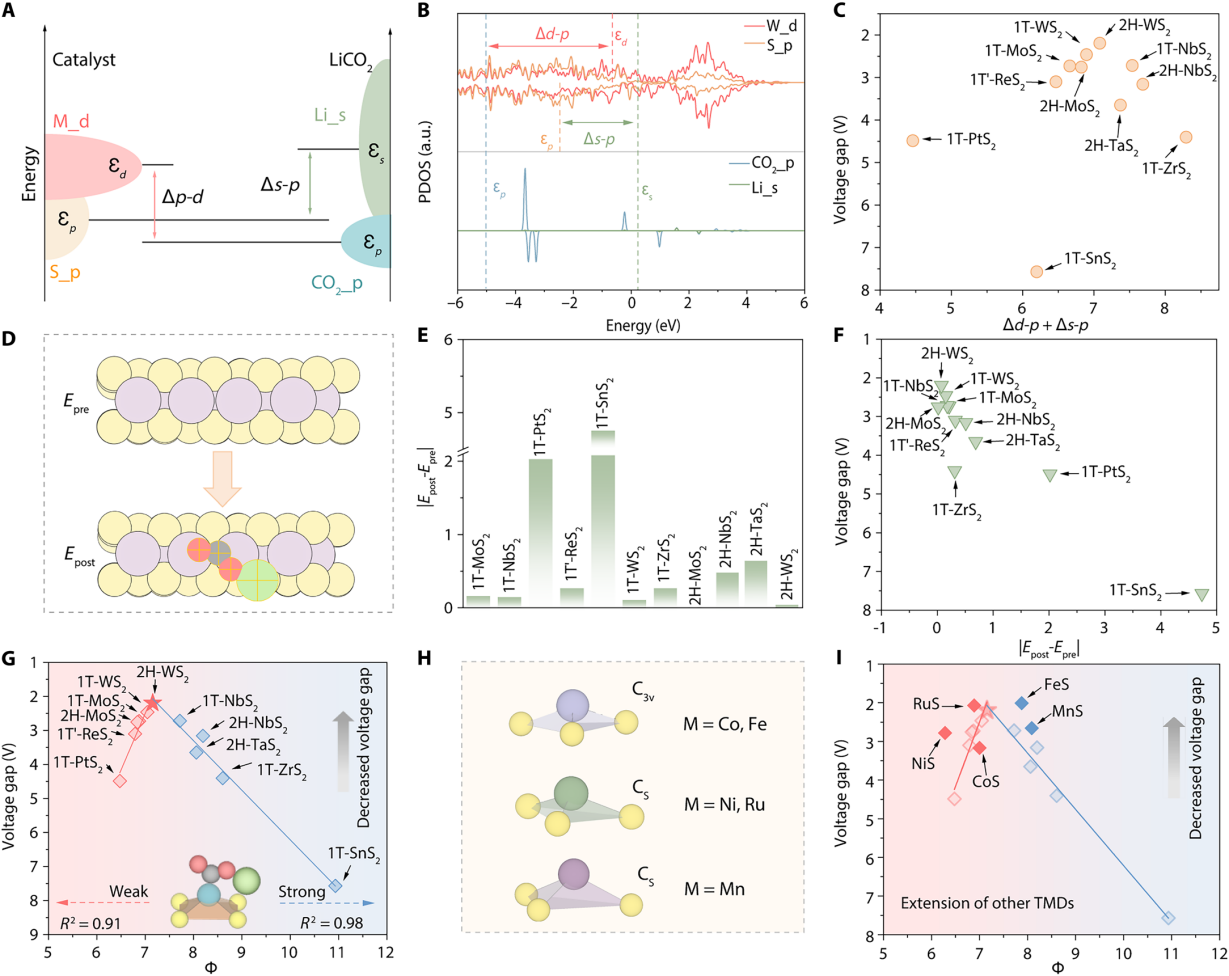
Construction of descriptors from electronic and structural effects. (**A**) Schematic diagram of *d-p* and *s-p* orbital coupling. (**B**) Projected density of states (PDOS) of 2H-WS_2_ and LiCO_2_ intermediate, W *d*-band center (ε*_d_*), S *p*-band center (ε*_p_*), Li *s*-band center (ε*_s_*), CO_2_ *p*-band center (ε*_p_*), the energy gap [Δ(*p-d*)] between W ε*_d_* and CO_2_ ε*_p_*, and the energy gap [Δ(*s-p*)] between Li ε*_s_* and S ε*_p_* are plotted. (**C**) Voltage gap versus orbital coupling gap (Δ*p-d* + Δ*s-p*). (**D**) Schematic diagram of structural reconstruction. (**E**) The structural reconstruction energy and (**F**) voltage gap versus structural reconstruction energy (*|E*_post_*-E*_pre_*|*). (**G**) The volcano-shaped relationship between descriptor Φ and voltage gap. (**H**) Extended symmetry-breaking structure and (**I**) corresponding volcano-shaped Φ-voltage gap plot. a.u., arbitrary units.

Building on the established foundation, the voltage gap in Li-CO_2_ batteries is fundamentally governed by the adsorption-free energy of the LiCO_2_ intermediate, under the concerted influence of M-S bond energetics and the electronic characteristics of the S *p*-band center. From the perspective of molecular orbital theory, the Li and C/O atoms within the LiCO_2_ intermediate engage in specific interactions with the anionic S sites and metal cations in layered TMDs, respectively. This distinctive charge distribution gives rise to a theoretical framework of dual-channel electronic coupling. Specifically, this coupling mechanism comprises two central processes: *d-p* orbital hybridization between the transition metal *d*-orbitals and the *p*-orbitals of the CO_2_ group in Li-CO_2_ and *s-p* orbital interaction between the S *p*-orbitals and the s-orbitals of the Li atom (Fig. 4A). We define the orbital coupling gap (Δ*d-p* + Δ*s-p*) as the energetic superposition of these two hybridization processes. This composite parameter essentially characterizes the energy level alignment between the frontier molecular orbitals of the layered material and the reaction intermediate, where a reduction in its magnitude directly correlates with diminished interfacial charge-transfer resistance and enhanced electronic coupling strength.

Using PDOS calculations, we systematically analyzed the evolution of the orbital coupling gap before and after LiCO_2_ adsorption, with 2H-WS_2_ as a model system (Fig. 4B and figs. S20 and S21). The consistency between theoretical analysis and computational results establishes the existence of a dual *d-p* and *s-p* hybridization-mediated electronic coupling mechanism. It should be clarified that the orbital coupling gap does not follow a smaller is better trend. Consistent with the Sabatier principle, an optimal interaction strength exists for the key intermediate LiCO_2_. If the gap is too large, then LiCO_2_ stabilization is insufficient. If it is too small, then overly strong M-O bonds poison active sites. Therefore, the orbital coupling gap exhibits an inherent volcano-shaped relationship with the voltage gap as presented in Fig. 4C. A strong correlation exists between the orbital coupling gap and the voltage gap, validating its role as an electronic descriptor. However, notable deviations of systems such as 1T-PtS_2_, 1T-SnS_2_, and 2H-TaS_2_ from the volcano plot trend reveal the inherent limitations of a purely electronic descriptor. These deviations fundamentally reflect the neglect of dynamic structural reconstruction of the catalyst during the reaction process. When the reaction intermediate interacts with the active site, the local coordination environment undergoes adaptive geometric rearrangements. The energy cost associated with this structural evolution directly affects the reversibility of the catalytic cycle. Therefore, constructing a universally predictive model necessitates the simultaneous consideration of both electronic structure modulation and the dynamic response of the geometric configuration. Only by establishing a composite descriptor incorporating both electronic and structural degrees of freedom can the complex behavior of the catalytic system under realistic reaction conditions be accurately captured. This insight motivated our proposal of a composite descriptor strategy that integrates both orbital coupling and structural reconstruction.

Motivated by the impact of structural evolution on the voltage gap, we conducted an in-depth study of the structural transformation behavior of catalytic materials under electrochemical operation. We introduced the structural reconstruction energy (*|E*_post_*-E*_pre_*|*) as a critical descriptor, where *E*_pre_ and *E*_post_ represent the ground-state energies of the catalytic system before and after LiCO_2_ adsorption, respectively (Fig. 4D). This parameter establishes a quantitative relationship between catalyst structural stability and electrochemical performance, fundamentally reflecting the configuration-preserving capability of active sites during interfacial processes. Lower reconstruction energy values signify superior structural reversibility and enhanced cycling stability within the Li-CO_2_ battery system. Systematic computational results reveal a definitive correlation between structural reconstruction energy and voltage gap (Fig. 4, E and F). Notably, 1T-PtS_2_, 1T-SnS_2_, and 2H-TaS_2_, which exhibit substantial deviation from the volcano-type trend in the electronic descriptor mapping (Fig. 4C), concurrently demonstrate the highest structural reconstruction energy values. This direct correlation explains their deviation. The substantial structural distortion upon LiCO_2_ adsorption incurs an energetic penalty that is not captured by a static electronic descriptor, thereby disrupting the expected volcano relationship. This finding underscores the limitation of unitary electronic descriptors: Relying solely on orbital coupling interactions proves insufficient for accurately capturing the dynamic behavior of catalytic systems under operational conditions. When reaction intermediates interact with active sites, substantial geometric reconstruction occurs within the local coordination environment, with the associated energy penalty directly governing both the thermodynamic reversibility and kinetic efficiency of the catalytic cycle.

Building on these insights, we developed a dual-parameter descriptor Φ that integrates both electronic and structural contributions, mathematically defined as λ_1_Δ*d-p* + λ_2_Δ*s-p* + λ_3_*|E*_post_*-E*_pre_*|*. From a physical consistency perspective, the three components Δ*d-p*, Δ*s-p*, and *|E*_post_*-E*_pre_*|* share the same energy unit (electron volts) and exhibit comparable numerical ranges (0.02 to 5.65 eV) across all investigated TMD systems. They represent coordinate processes in the catalytic mechanism, where Δ*d-p* and Δ*s-p* describe electronic coupling via orbital interactions, while *|E*_post_*-E*_pre_*|* captures the structural response upon intermediate adsorption. These terms collectively determine the adsorption energetics and reversibility of LiCO_2_, and weighting them differently would artificially bias the energy balance of the adsorption process. Guided by the maximum entropy principle, equal weighting represents the least biased choice in the absence of prior knowledge favoring any particular component. Following Occam’s razor, the equal-weight scheme introduces no additional free parameters, avoiding overfitting risks given the limited sample size.

To empirically validate this choice, we performed a systematic sensitivity analysis by allowing each λ to vary independently of 0.2 to 2.8 in increments of 0.01 while monitoring the *R*^2^ (fig. S22). This range was selected for two reasons: (i) it is centered at unity and extends symmetrically to comprehensively explore the parameter space around the equal-weight scheme; (ii) within this range, *R*^2^ remains above 0.93 across all combinations, whereas scanning beyond this range causes *R*^2^ to drop below 0.93, indicating that regions outside this interval lack meaningful correlation and are irrelevant for further consideration. The results demonstrate that λ_2_ optimally converges to exactly 1.000, providing direct empirical support for λ_2_ = 1. The optimal λ_1_ value of 1.191 represents a marginal difference from unity, with the corresponding improvement in *R*^2^ upon adjusting λ_1_ from 1 to its optimal value being negligible (Δ*R*^2^ = 0.0016), confirming that λ_1_ = 1 lies within a plateau region of the optimization landscape. Although λ_3_ exhibits the largest deviation with an optimal value of 0.809, the resulting improvement in *R*^2^ is merely 0.0045, which lacks statistical substance given the limited sample size. The maximum fitting gain achievable by deviating from equal weighting does not exceed 0.46%, rendering the equal-weight formulation clearly superior when weighed against the overfitting risk associated with increased parametric complexity. The equally weighted composite descriptor Φ = λ_1_Δ*d-p* + λ_2_Δ*s-p* + λ_3_*|E*_post_*-E*_pre_*|* (λ_1_ = λ_2_ = λ_3_ = 1) is robustly supported by both domain theory and empirical data, establishing it as a reasonable and reliable parameterization in this study.

As shown in Fig. 4G, Φ exhibits a characteristic volcano-shaped relationship with the voltage gap, directly reflecting the intrinsic trade-off between electronic and structural effects. Notably, the previously anomalous systems including 1T-PtS_2_, 1T-SnS_2_, and 2H-TaS_2_ now align perfectly with this volcano plot. This confirms that incorporating structural reconstruction energy resolves the discrepancy and provides a complete description of the catalytic behavior. Moderate electronic coupling both promotes reaction kinetics and prevents site deactivation caused by excessively strong coupling. Since this process is inherently accompanied by structural reconstruction, incorporating the structural reconstruction energy refines the volcano relationship. Consequently, optimal performance is achieved not at the extremes but in the intermediate Φ region. This descriptor constructs a direct bridge connecting microscopic electronic structure with macroscopic battery performance, providing an effective strategy to circumvent the complexities inherent in conventional mechanistic analyses. Among the investigated material systems, 2H-WS_2_ demonstrates exceptional performance potential with its near-optimal Φ value of 7.16, validating the descriptor’s utility in guiding rational catalyst design for Li-CO_2_ batteries.

The generality of Φ was further validated through electrochemical evaluation of Li-CO_2_ batteries based on additional layered TMDs with symmetry-breaking configurations (Fig. 4H and fig. S23). As illustrated in Fig. 4I, the volcano-shaped relationship between Φ and the voltage gap, established earlier, also holds for these TMD systems. These findings elucidate the connection between the intrinsic properties of TMDs and the Li-CO_2_ voltage gap, highlighting two key criteria for minimizing the voltage gap: a moderate orbital coupling gap to facilitate reaction kinetics and a low structural reconstruction energy to ensure catalytic reversibility during cycling. Thus, the orbital coupling gap and structural reconstruction energy, which respectively characterize the binding strength and structural stability with the LiCO_2_ intermediate, can serve as effective indicators for designing high-performance catalysts with minimal voltage gaps. Overall, these results verify that a material’s adaptability to LiCO_2_ is an intrinsic factor governing the voltage gap in Li-CO_2_ batteries, demonstrating that Φ can be regarded as a universal descriptor for predicting the voltage gap.

To evaluate the descriptor Φ for predicting voltage gaps in symmetry-breaking systems, we synthesized four layered TMDs (SnS_2_, ReS_2_, MoS_2_, and WS_2_). These materials were selected because they (i) span a broad Φ range (6.79 to 10.93) covering the volcano peak and both branches, (ii) represent diverse symmetry-breaking motifs (C_4v_ for WS_2_/MoS_2_ and C_s_ with varying distortion for SnS_2_/ReS_2_), and (iii) have controlled morphologies with similar interlayer structures, thereby excluding interference of interlayer structures with surface electronic properties. This allows for a comprehensive experimental validation of Φ. First, x-ray diffraction (XRD) analysis verified the high crystallinity of all materials, effectively ruling out any substantial influence from crystal imperfections or lattice distortions (fig. S24). Valence band spectra further confirmed consistent semiconducting characteristics across all four samples, thereby eliminating electrical conductivity variations as a determining factor in performance differences (figs. S25 to S28). Having established this, we confirmed that the synthesized MS_2_/carbon paper (CP) nanosheets exhibit uniform size and crystallinity with vertically aligned morphology (figs. S29 and S30), maximizing edge site exposure (*33*). This specific configuration was engineered to decouple the effects of morphology and stacking, ensuring that edge structures remain the primary variable under investigation. Furthermore, high-resolution transmission electron microscopy observations of abundant layered lattice fringes provide definitive evidence for exposed edge sites (fig. S31). The termination points of these fringes, where the periodic crystal structure ends, offer atomic-scale verification of the symmetry-breaking configurations.

**Fig. 5.**
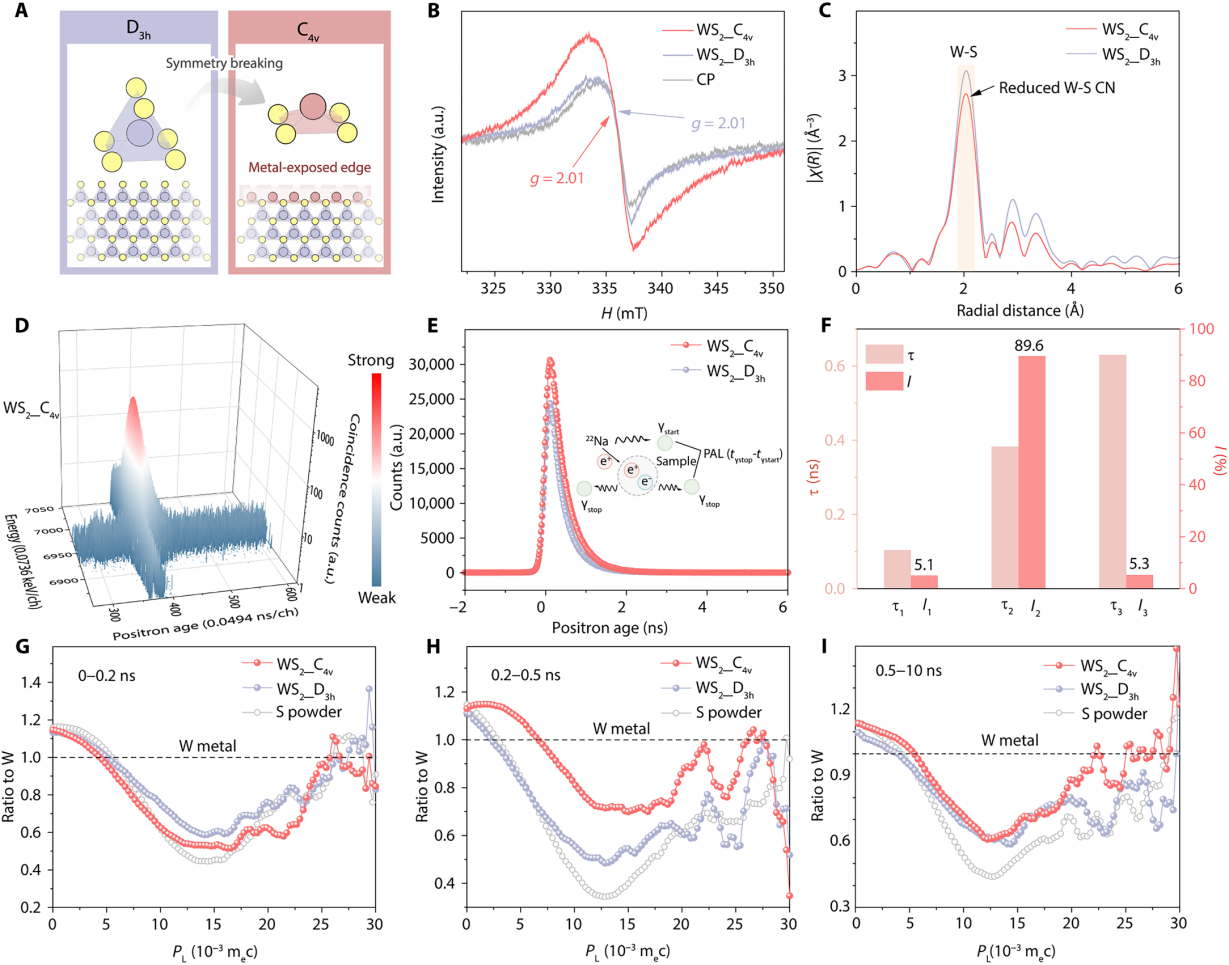
Experimental validation of symmetry-breaking features. (**A**) Schematic diagram illustrating the symmetry breaking achieved by controlling the growth orientation. (**B**) EPR spectra of WS_2__C_4v_ and WS_2__D_3h_ samples. (**C**) Fourier-transformed R-space spectra of the W L_3_-edge. (**D**) AMOC spectrum of WS_2__C_4v_. (**E**) Positron annihilation lifetime (PAL) spectra of WS_2__C_4v_ and WS_2__D_3h_ acquired via AMOC spectroscopy. (**F**) Positron annihilation lifetime components and their intensities of WS_2__C_4v_. Ratio curves of Doppler broadening spectra (derived from their AMOC spectra) of WS_2__C_4v_ and WS_2__D_3h_ samples in the positron age regions of (**G**) 0 to 0.2 ns, (**H**) 0.2 to 0.5 ns, and (**I**) 0.5 to 10 ns with respect to that of pure W metal.

Among these samples, WS_2_ was identified by the Φ descriptor as having the smallest predicted voltage gap. Guided by this theoretical prediction, we selected WS_2_ for targeted validation of its symmetry-breaking structure featuring exposed cation sites (Fig. 5A). The presence of intrinsic symmetry-breaking in the edge structure was verified by electron paramagnetic resonance (EPR). To clearly distinguish between the different symmetries, the as-synthesized and commercial WS_2_ samples are denoted as WS_2__C_4v_ and WS_2__D_3h_, respectively. The EPR signal of unpaired electrons at *g* = 2.01 was markedly more intense in WS_2__C_4v_ than in WS_2__D_3h_ (Fig. 5B), indicating a higher density of symmetry-breaking edge sites in the former. This finding aligns with the conclusions from prior spin density calculations. Furthermore, the Fourier-transformed R-space spectra and extended x-ray absorption fine structure (EXAFS) analysis revealed a lower W-S coordination number in WS_2__C_4v_ (Fig. 5C), which we attribute to the increased prevalence of unsaturated W-S bonds at the symmetry-breaking edge sites.

As mentioned above, this viewpoint is further demonstrated by the positron annihilation age-momentum correlation (AMOC) spectroscopy (Fig. 5D). Advanced AMOC spectroscopy, by simultaneously measuring positron annihilation lifetimes and the momenta of electron-positron pairs (*34*, *35*), enables the separation of different positron annihilation states, thus emerging as a powerful technique for elucidating a local atomic environment around the symmetry-breaking feature. The positron annihilation lifetime spectra of WS_2__C_4v_ and WS_2__D_3h_ (Fig. 5E) obtained by AMOC spectroscopy yield three lifetime components (τ_1_, τ_2_, and τ_3_) as shown in Fig. 5F and fig. S32. The shortest positron annihilation lifetime (τ_1_) corresponds to the bulk material and isolated tiny vacancies, the intermediate lifetime (τ_2_) is associated with small vacancy associates or defect clusters, and the longest lifetime (τ_3_) is linked to larger defects or vacancy clusters (*36*). The intensities of positron lifetime components quantify the concentration of defects. The value of *I*_2_ = 89.6% indicates that intermediate lifetimes τ_2_ dominate in WS_2__C_4v_, with predominantly small vacancy associates present. These small vacancy associates are associated with the symmetry-breaking characteristics. Moreover, AMOC spectra, as a function of positron age and electron momentum, reveal positron annihilation characteristics across multiple spatial scales, aiding the understanding of atomic local environments in symmetry-breaking engineering. Figure 5 (G to I) and fig. S33 present the ratio curves of Doppler broadening for WS_2__C_4v_, WS_2__D_3h_, and S powder with respect to that of W which are derived from those of AMOC spectra in different positron age regions. It is evident that the electronic momentum of WS_2__C_4v_ and WS_2__D_3h_ are basically consistent in the positron age ranges of 0 to 0.2 ns and 0.5 to 10 ns, while the differences are mainly concentrated in the annihilation time range of 0.2 to 0.5 ns, indicating the difference in atomic local environments. The AMOC spectrum of WS_2__C_4v_ approaches 1 within the 0.2- to 0.5-ns positron age range, indicating that the W/S ratio in WS_2__C_4v_ is higher than that in WS_2__D_3h_ under a symmetry-breaking atomic local environment. These results clearly demonstrate the successful realization of symmetry-breaking edge structures, which is consistent with our theoretical calculation model, where the symmetry-breaking edges are primarily W exposed.

Prior DFT calculations identified Li_2_CO_3_ as the final discharge product in a pure CO_2_ atmosphere. To validate the proposed reaction mechanism, we systematically characterized the morphological and compositional evolution during cycling, along with the complete discharge profiles. As shown in fig. S34, complete discharge tests of WS_2_ in CO_2_ and Ar atmospheres confirmed that CO_2_ actively participates as a reactant. Ex situ XRD analysis of the four discharged cathodes revealed distinct diffraction peaks of Li_2_CO_3_. Notably, after charging, trace residual Li_2_CO_3_ was detected in three of the four cathodes, whereas none was observed in the WS_2_ cathode (figs. S35 to S38), indicating its efficient decomposition of Li_2_CO_3_. Ex situ Raman spectroscopy of WS_2_ (fig. S39) further corroborated the reaction pathway involving Li_2_CO_3_. For WS_2__D_3h_, the XRD pattern after deep discharge (fig. S40) similarly confirmed highly crystalline Li_2_CO_3_ as the primary discharge product, consistent with DFT predictions. Together, these results confirm the superior Li_2_CO_3_ decomposition capability of the WS_2_ cathode over the other materials studied, thereby validating the rationality of constructing the descriptor Φ based on Li_2_CO_3_ as the final discharge product.

**Fig. 6.**
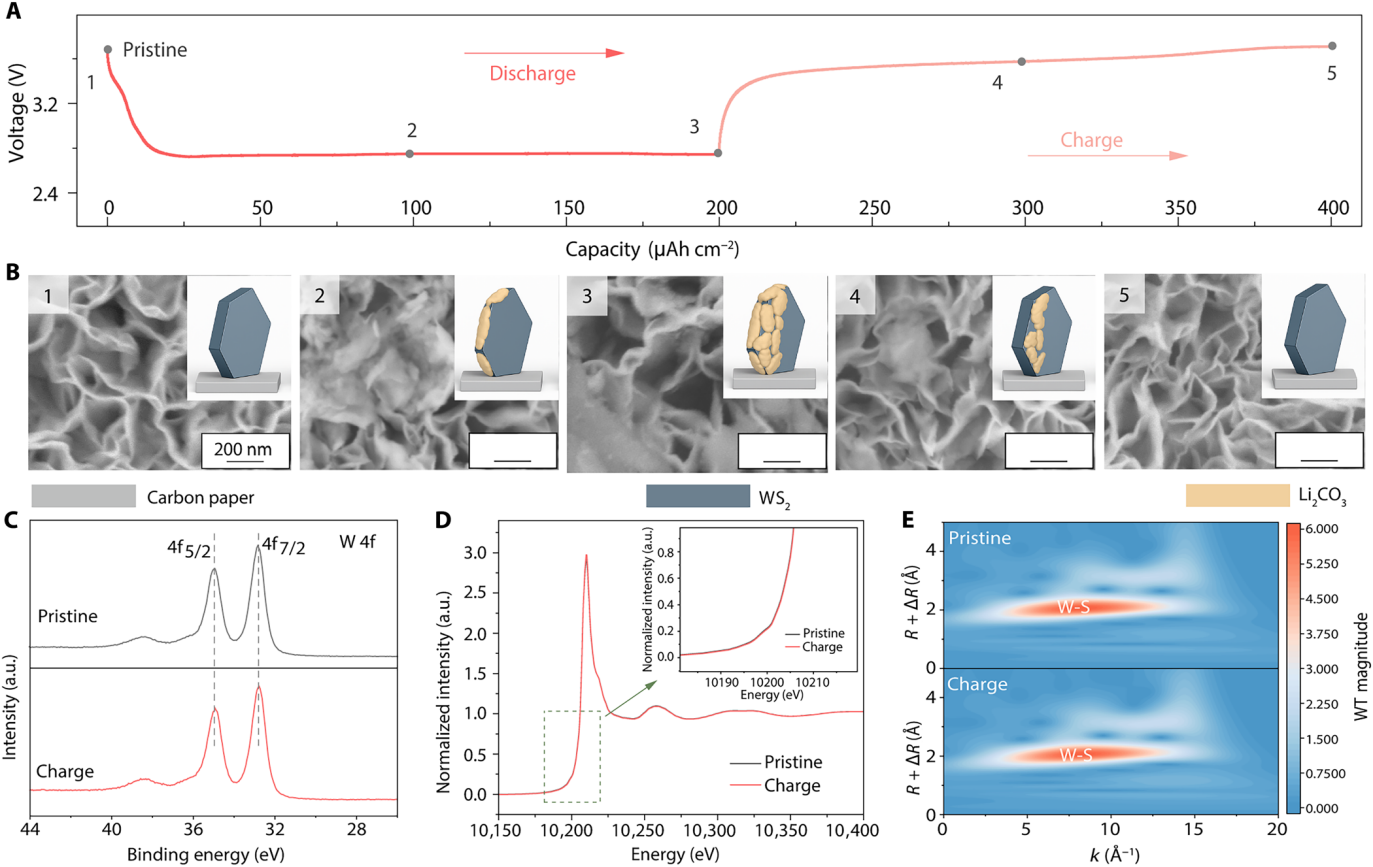
Promotion of Li_2_CO_3_ nucleation/decomposition by robust symmetry-breaking structural motifs. (**A**) Presented are the GDC curves for the WS_2_ cathode, recorded under a current density of 20 μA cm^−2^ and a limited capacity of 200 μAh cm^−2^. Five designated states are indicated: the initial pristine material (1), during discharge to 100 (2) and 200 (3) μAh cm^−2^, and during charge to 100 (4) and 200 (5) μAh cm^−2^. (**B**) SEM images corresponding to each of these states. Scale bars, 200 nm. The inset shows a schematic depicting Li_2_CO_3_ nucleation/decomposition sites during the discharge/charge process, derived from SEM image analysis. (**C**) W 4f spectra of WS_2_ under pristine and charged states. (**D**) XANES spectra and (**E**) WT of *k*^2^-weighted EXAFS signals of the W L_3_-edge under pristine and charged states.

The mechanistic origin of the efficient Li_2_CO_3_ decomposition on WS_2_ was investigated by tracing the local evolution of its nucleation and decomposition sites during discharge/charge processes using an ex situ scanning electron microscope (SEM). As shown in Fig. 6 (A and B), Li_2_CO_3_ nucleation during discharge occurs preferentially at the C_4v_-symmetry edges of WS_2_ before propagating to the D_3h_ basal planes. The decomposition during charging initiates at these same C_4v_-edge sites. This site specificity in both nucleation and decomposition confirms that the C_4v_ configuration serves as the primary active center, consistent with our theoretical proposition. The structural integrity of all four samples was maintained throughout cycling, as confirmed by postcycling characterization (fig. S41), indicating preservation of the catalytic active sites. Furthermore, XRD, Raman, and x-ray photoelectron spectroscopy analyses corroborate that WS_2_ retains its original phase and chemical structure during repeated cycling (Fig. 6C and figs. S42 to S44). The stability was further verified by x-ray absorption near-edge structure (XANES) spectra and wavelet transform (WT) analysis of the *k*^2^-weighted EXAFS signals, which reveal that the outstanding Li-CO_2_ performance of WS_2_ is attributable to its robust structural and electronic integrity under operating conditions (Fig. 6, D and E, and fig. S45).

**Fig. 7.**
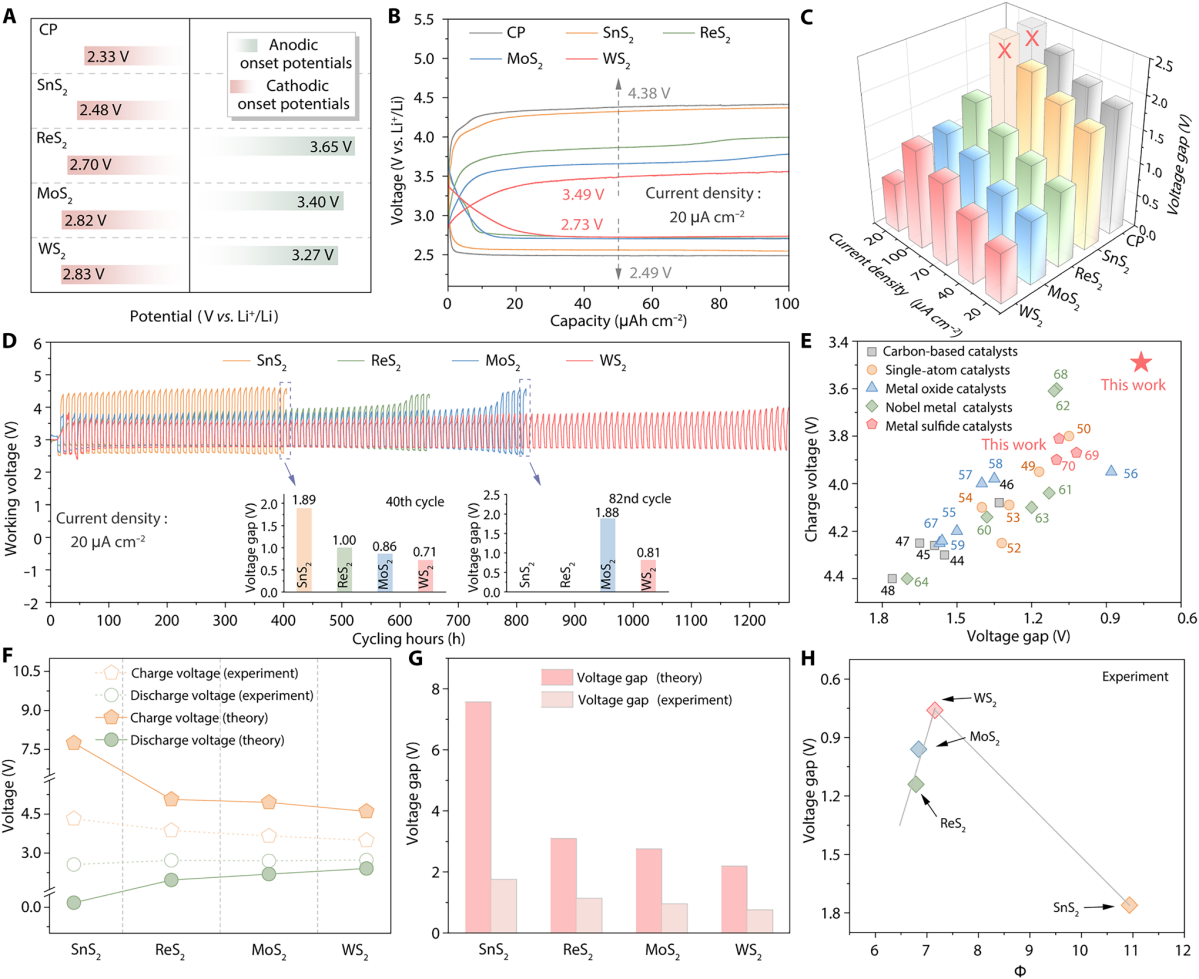
Electrochemical performance of Li-CO_2_ battery. (**A**) Onset potential measured at 40 μA cm^−2^ from CV curves. (**B**) GDC profiles at a capacity limit of 100 μAh cm^−2^ and a current density of 20 μA cm^−2^. (**C**) Voltage gaps measured at current densities ranging from 20 to 100 μA cm^−2^. (**D**) Cycling performance of the four cathodes at 20 μA cm^−2^. (**E**) Performance comparison with previously reported catalysts (*44*–*50*, *52*–*64*, *67*–*70*). (**F** and **G**) Theoretical and experimental values for the discharge/charge voltage and voltage gap. (**H**) The volcano-shaped relationship between the descriptor Φ and the experimental voltage gap.

A series of electrochemical performance evaluations was conducted on Li-CO_2_ batteries to validate the effectiveness of the descriptor Φ in predicting experimental catalytic activity. The kinetics of the CO_2_RR and CO_2_ER were probed by determining the onset potential at a current density of 40 μA cm^−2^ from cyclic voltammetry (CV) curves (Fig. 7A and fig. S46). The results show that the cathodic onset potentials increase in the order of CP, SnS_2_, ReS_2_, MoS_2_, and WS_2_, whereas the anodic onset potentials follow the reverse trend. Notably, the anodic onset potentials for CP and SnS_2_ could not be determined under this criterion due to their negligible anodic currents. This indicates that WS_2_ exhibits the most favorable CO_2_RR and CO_2_ER kinetics, aligning with prior theoretical predictions. Galvanostatic discharge-charge (GDC) profiles were recorded at 20 μA cm^−2^ with a fixed capacity of 100 μAh cm^−2^ (Fig. 7B). The resulting voltage gaps for CP, SnS_2_, ReS_2_, MoS_2_, and WS_2_ are 1.89, 1.76, 1.14, 0.96, and 0.76 V, respectively. Rate performance, median discharge/charge voltages, and energy efficiencies at various current densities are provided in figs. S47 to S49 and table S2. The experimental voltage gaps are consistent with trends predicted by DFT calculations. As the current density increases from 20 to 100 μA cm^−2^, the voltage gaps for all materials retain their relative order (Fig. 7C). The voltage gaps for CP and SnS_2_ at 100 μA cm^−2^ are not attainable, as their charge voltages reach the cutoff limit before full capacity delivery. Cycling stability tests across the same current density range (Fig. 7D and figs. S50 to S53) further confirm that WS_2_ achieves the smallest voltage gap and the longest cycle life among all samples.

These results collectively demonstrate that WS_2_ with C_4v_ symmetry not only delivers an ultralow charge voltage and a minimal voltage gap (0.76 V) at 20 μA cm^−2^ but also exhibits exceptional cycling stability (1268 hours), outperforming other reported materials (Fig. 7E and table S3) as well as WS_2_ with D_3h_ basal plane configuration (fig. S54). Electrochemical impedance spectroscopy further reveals a lower charge-transfer resistance for WS_2__C_4v_ than WS_2__D_3h_ (fig. S55), confirming that the C_4v_ edge sites facilitate faster interfacial charge transfer. After ~400 hours of operation, the voltage gap maintains the trend of WS_2_ < MoS_2_ < ReS_2_ < SnS_2_, confirming the predictive utility of Φ throughout the entire cycling process. Even after 1268 hours, WS_2_ retains a relatively low voltage gap (1.09 V), further attesting to the robustness of the descriptor. Moreover, the stable operation of coin cells over a wide temperature range (25° to 80°C) and the performance integrity of flexible pouch cells under bending angles of 0-180^o^ substantiate the practical feasibility of WS_2_-based Li-CO_2_ batteries (figs. S56 and S57). A comparison between theoretical and experimental charge/discharge voltages and voltage gaps is presented in Fig. 7 (F and G). It should be noted that direct quantitative comparison between the absolute values of DFT-calculated and experimentally measured voltage gaps is inherently challenging. The theoretical voltage gap represents an intrinsic thermodynamic limit derived from an idealized surface model under static conditions, whereas the experimental value encompasses complex electrolyte interactions and electrochemical environments that cannot be fully captured by computational methods. Nevertheless, the relative activity trends predicted by DFT, particularly the material performance ranking of WS_2_ > MoS_2_ > ReS_2_ > SnS_2_, show excellent consistency with experimental observations, validating the predictive capability of our descriptor Φ. This correlation is further manifested as a volcano-type relationship between Φ and the experimental voltage gap in Fig. 7H, highlighting the reliability of Φ as a predictor for the voltage gap in Li-CO_2_ batteries.

## DISCUSSION

In summary, we have developed a dual-parameter descriptor Φ, which incorporates the synergistic effects of anions and cations to guide the exploration of the minimal voltage gaps in Li-CO_2_ batteries. Our findings reveal that anions not only modulate the orbital coupling with LiCO_2_ intermediates but also contribute to structural stabilization. The Φ descriptor, integrating both electronic and structural factors, demonstrates that a moderate orbital coupling gap enhances the interfacial conversion kinetics of LiCO_2_ intermediates. Concurrently, a low structural reconstruction energy ensures excellent reversibility of the catalyst during cycling. This design principle was successfully extended to other layered TMDs. Using four representative materials (SnS_2_, ReS_2_, MoS_2_, and WS_2_) as model systems, the descriptor Φ accurately predicted the trends in discharge/charge voltages and voltage gaps. Among them, WS_2_, which has a moderate Φ value, exhibited the smallest voltage gap, in strong agreement with experimental measurements. Φ provides a rational and transferable framework for minimizing the voltage gap in Li-CO_2_ batteries, showing promise as a universal tool for the design of advanced electrocatalysts.

## MATERIALS AND METHODS

### Chemicals

Dimethyl sulfoxide (DMSO, anhydrous), 1-ethyl-3-methylimidazolium tetrafluoroborate (EMIM BF4), lithium bis((trifluoromethyl)sulfonyl)azanide (LiTFSI, 99%), thiourea (CH_4_N_2_S, 99%), thioacetamide (C_2_H_5_NS, 99%), tin (IV) chloride pentahydrate (SnCl_4_·5H_2_O, 99.995%), ammonium perrhenate (NH_4_ReO_4_, 99%), hydroxylammonium chloride (H_3_NO·HCl, 99.99%), ammonium molybdate tetrahydrate [(NH_4_)_6_Mo_7_O_24_·4H_2_O, 99.9%], and ammonium metatungstate hydrate [(NH_4_)_6_H_2_W_12_O_40_·XH_2_O, 99.5%] were purchased from Aladdin. All chemicals were used without any further purification.

### Preparation of CP

The CP (2.5 cm by 3 cm) was obtained by the reaction of CP (TGP-H-60) at 650°C for 20 min in air.

### Preparation of SnS_2_/CP

SnS_2_/CP was prepared by a solvothermal method on CP (*37*). Typically, tin (IV) chloride pentahydrate (SnCl_4_·5H_2_O) (73 mg) and thioacetamide (C_2_H_5_NS) (146 mg) were dissolved in 20 ml of isopropanol and stirred for 30 min, and then the solution was transferred to a 45-ml Teflon-lined stainless steel autoclave. The CP (2.5 cm by 3 cm) was placed in above solution and heated at 180°C for 24 hours. After cooling, the CP was removed and washed several times with deionized water and ethanol. Last, it was dried overnight at 60°C in a vacuum oven.

### Preparation of ReS_2_/CP

According to previous reports, the ReS_2_/CP was prepared using the hydrothermal method (*38*). In a typical procedure, 107.3 mg of ammonium perrhenate (NH_4_ReO_4_), 136.7 mg of thiourea, and 83 mg of hydroxylammonium chloride (H_3_NO·HCl) were dispersed in 20 ml of deionized water followed by stirring at room temperature for 30 min to form a homogeneous solution. The CP (2.5 cm by 3 cm) was transferred into a 45-ml Teflon-lined stainless steel autoclave and heated at 220°C for 20 hours. After cooling, the obtained ReS_2_/CP was washed several times with deionized water and ethanol and then dried overnight in a vacuum oven at 60°C.

### Preparation of MoS_2_/CP

The MoS_2_/CP was prepared by using hydrothermal method (*36*). Typically, 62 mg of ammonium molybdate tetrahydrate [(NH_4_)_6_Mo_7_O_24_·4H_2_O] and 114.0 mg of thiourea (CH_4_N_2_S) were dissolved in 20 ml of deionized water by stirring 30 min to form a homogeneous solution. Then, the CP (2.5 cm by 3 cm) was placed in above solution, and it was transferred into a 45-ml Teflon-lined autoclave, maintained at 220°C for 12 hours, and allowed to cool to room temperature naturally. The final product (MoS_2_/CP) was washed with water and absolute ethanol several times and dried at 60°C under vacuum.

### Preparation of WS_2_/CP

The WS_2_/CP was prepared using a one-step solvothermal method. Typically, 0.84 g of ammonium metatungstate hydrate [(NH_4_)_6_H_2_W_12_O_40_·XH_2_O] and 1.28 g of thioacetamide (C_2_H_5_NS) were dispersed in 20 ml of *N*,*N*-dimethylformamide and stirred for 30 min. The CP (2.5 cm by 3 cm) was added to the aforementioned mixture and was then transferred to a 45-ml Teflon-lined autoclave, reacting at 200°C for 24 hours. After natural cooling, the obtained product was washed several times with deionized water and ethanol and then dried overnight in a vacuum oven at 60°C. The final product (WS_2_/CP) was obtained by calcining at 400°C for 2 hours in Ar gas.

### Characterization

The structure of samples or cathodes was characterized by powder XRD patterns (SmartLab SE, Rigaku), Raman spectroscopies (LabRAM HR Evolution). The surface compositions of materials and valence of elements were carried out by x-ray photoelectron spectroscopy (Thermo Scientific ESCALAB 250Xi). SEM images were collected on a Gemini SEM 360 SEM. TEM images were taken on a JEM-2100 Plus transmission electron microscope. EPR spectra were collected using a JEOL JES-FA200 electron spin resonance spectrometer. The AMOC spectroscopy data were collected at room temperature in vacuum using a high counting rate AMOC system [approximately 180 counts per second (cps)], featuring a time resolution of about 200 ps and an energy resolution (full width at half maximum) of 1.22 keV at 511 keV. X-ray absorption fine structure (XAFS) spectra of the W L_3_ edge were collected at the beamline station BL14W1.

### Battery assembly and electrochemical measurements

Freestanding CP, SnS_2_/CP, ReS_2_/CP, MoS_2_/CP, and WS_2_/CP (12 mm in diameter) were directly used as the cathodes. The coin cells (2032, with 19 holes on the cathodes side) are assembled in an argon-filled glove box, with the concentration of water and oxygen kept below 0.01 parts per million. Whatman Glass fiber separators were used, and the liquid electrolyte was also prepared by dissolving 0.5 M LiTFSI in EMIM BF4/DMSO solution (volume ratio: 25/75). All prepared electrodes were sealed in glass test cells filled with CO_2_ for electrochemical testing. The galvanostatic charge-discharge tests were performed on a Neware battery testing system. For CV measurement, scan rates were chosen from 0.1 mV/s in the voltage range of 2.0 to 4.5 V on an electrochemical workstation (BioLogic VSP).

### Theoretical calculation

All spin-polarized DFT calculations were carried out using the Vienna ab initio simulation package with the projector augmented wave method (*39*, *40*). The generalized gradient approximation in the form of Perdew-Burke-Ernzerhof functional was applied to describe the exchange-correlation term (*41*). The MoS_2_ and WS_2_ (001) plane was modeled by building a 4 by 4 by 1 supercell, and the Brillouin zone was sampled using a Γ-centered k-point mesh of 2 by 2 by 1. The MS_2_ edges are constructed by cutting a single-layered MS_2_, with lattice constants greater than 12 Å in the periodic direction and greater than 15 Å in the vacuum direction. The cutoff energy for the plane-wave basis was set to 450 eV. All calculations adopted the empirically corrected Grimme’s DFT-D3 scheme to accurately describe van der Waals interactions (*42*). Atomic structures were fully relaxed until the force on each atom was <0.05 eV/Å, and the energy convergence criterion was set to 10^−5^ eV. The Li_2_CO_3_ decomposition barrier was evaluated by applying the CI-NEB method (*43*).

The Li-CO_2_ battery reaction process includes the CO_2_ reduction reaction (CRR) process during discharge and the CO_2_ evolution reaction process (CER) process during charge. The DFT calculations based on a four-electron reaction pathway were performed to explore the overall catalytic activity of CRR and CER. The reaction mechanism is depicted as follows (* denotes the catalytic surface): * + Li^+^ + e^−^ + CO_2_ ⇆ *LiCO_2_; *LiCO_2_ + Li^+^ + e^−^ + CO_2_ ⇆ *CO + Li_2_CO_3_; *CO + Li^+^ + e^−^ + CO_2_ ⇆ *LiC_2_O_3_; *LiC_2_O_3_ + Li^+^ + e^−^ ⇆ *C + *Li_2_CO_3_.

The Gibbs free energy change (Δ*G*) for each elementary step was computed by

$$\Delta G=\Delta E_{\text{DFT}}+\Delta E_{\text{ZPE}}–\mathrm{T\Delta}S+\Delta G_{U}$$

In this equation, Δ*E*_DFT_ is the reaction energy obtained from DFT calculations, Δ*E*_ZPE_ and Δ*S* are the zero-point energy correction and entropy change obtained from frequency calculations at 298.15 K. The Δ*G_U_* = −*eU* represents the influence of the applied electrode potential on the Gibbs free energy. The Gibbs free energy of Li^+^ + e^−^ is set to 0, which is balanced by bulk Li at *U* = 0 V. In the CRR process, each elementary step’s Gibbs free energy change is denoted by Δ*G*_1_, Δ*G*_2_, Δ*G*_3_, and Δ*G*_4_, respectively.

Gibbs free energy profiles were constructed in accordance with the Nernst equation

U=−ΔG/ne

In which *n* and *e* are the number of electrons transferred and the charge for each elementary step, respectively. The discharge/charge voltage (*U*_DC_/*U*_C_) is defined as the maximum/minimum voltage required to sustain the CRR/CER process. The *U*_eq_ represents the equilibrium voltage, with a higher *U*_DC_ value indicating better discharge ability and a lower *U*_C_ value signifying an easier charging process. The voltage gap is defined as *U*_C_ − *U*_DC_, and a smaller voltage gap indicates superior Li-CO_2_ battery performance.

## Supplementary Materials

### This PDF file includes:

Figs. S1 to S57 Tables S1 to S4 References
