# Supplementary material for: Synergistic anion-cation descriptor for bidirectional electrocatalyst in Li-CO2 battery
Source: Sci Adv. 2026 Jun 19;12(25):eaee9103. doi: 10.1126/sciadv.aee9103 (PMC13281819; doi:10.1126/sciadv.aee9103)
Supplement: Supplementary file 1 — Figs. S1 to S57 Tables S1 to S4 References [file sciadv.aee9103_sm.pdf]

Science Advances
AAAS

Supplementary Materials for

Synergistic anion-cation descriptor for bidirectional electrocatalyst in Li-CO_2 battery

Xingwu Zhai *et al.*

Corresponding author: Min Zhou, mzchem@ustc.edu.cn

Sci. Adv. **12**, eaee9103 (2026)
DOI: 10.1126/sciadv.aee9103

This PDF file includes:

Figs. S1 to S57
Tables S1 to S4
References

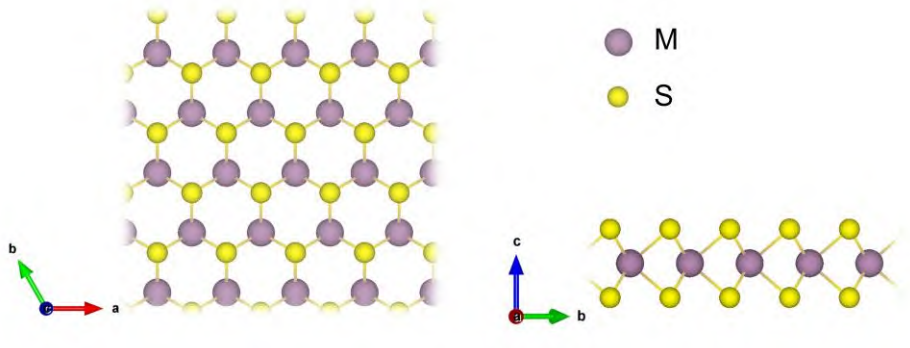

Fig. S1. Structural model. Structure of a typical 2H-MS_2 (M=Mo, W) basal plane.

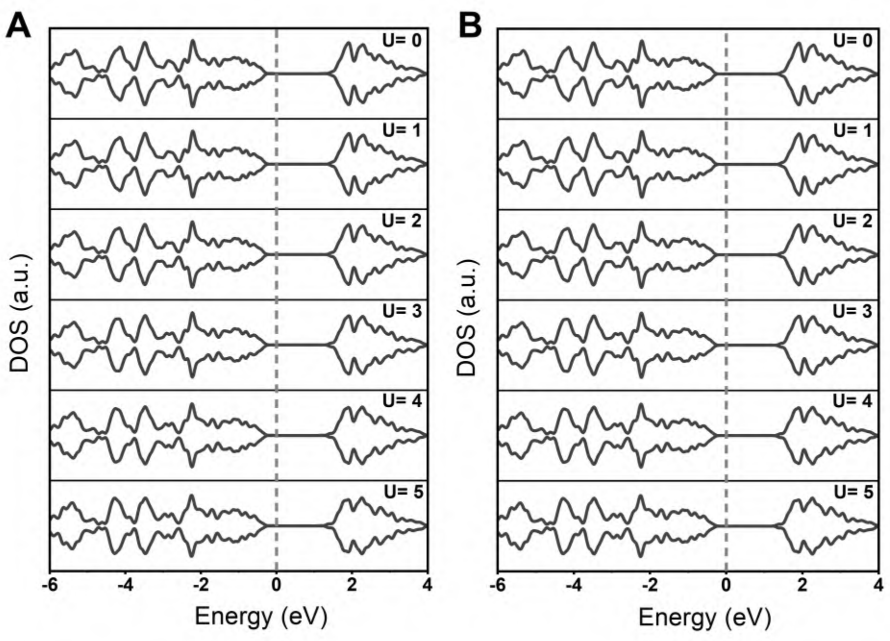

Fig. S2. DFT+U validation. The density of states (DOS) of (**A**) 2H-MoS_2 basal plane and (**B**) 2H-WS_2 basal plane as a function of U. The DOS does not change with the introduction of the U term into the computations. Thus, DFT+U method was not adopted in this work.

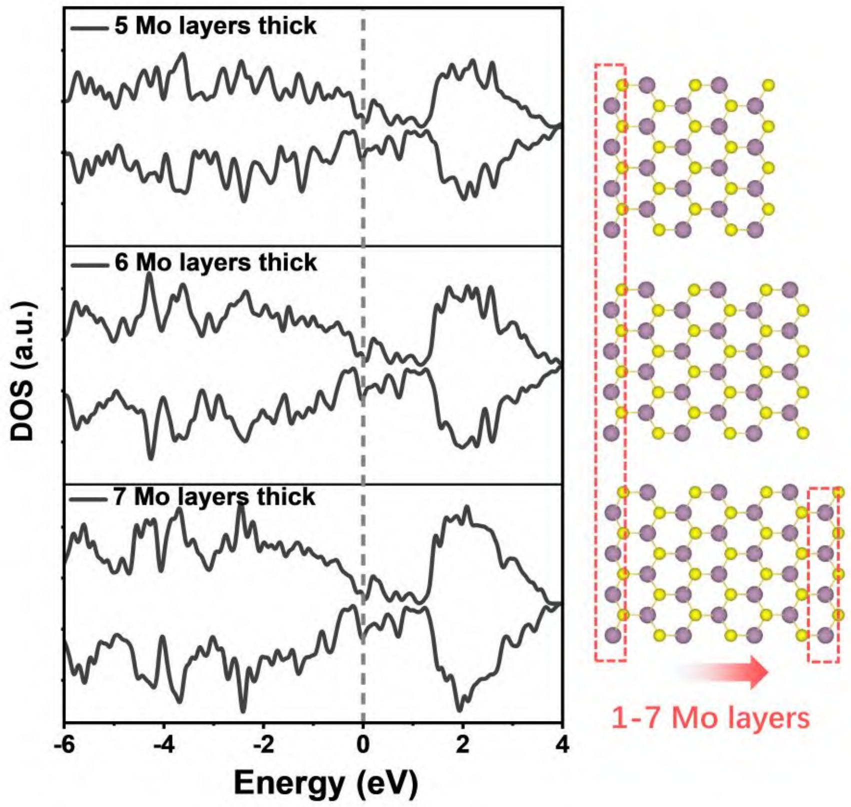

Fig. S3. Convergence test for 2H-MoS_2 zigzag edge. The DOS of 2H-MoS_2 zigzag edge with different Mo layers and corresponding structures. The DOS does not change significantly with the increase of the Mo layers suggesting that a thickness of 5 layers is sufficient for the calculation.

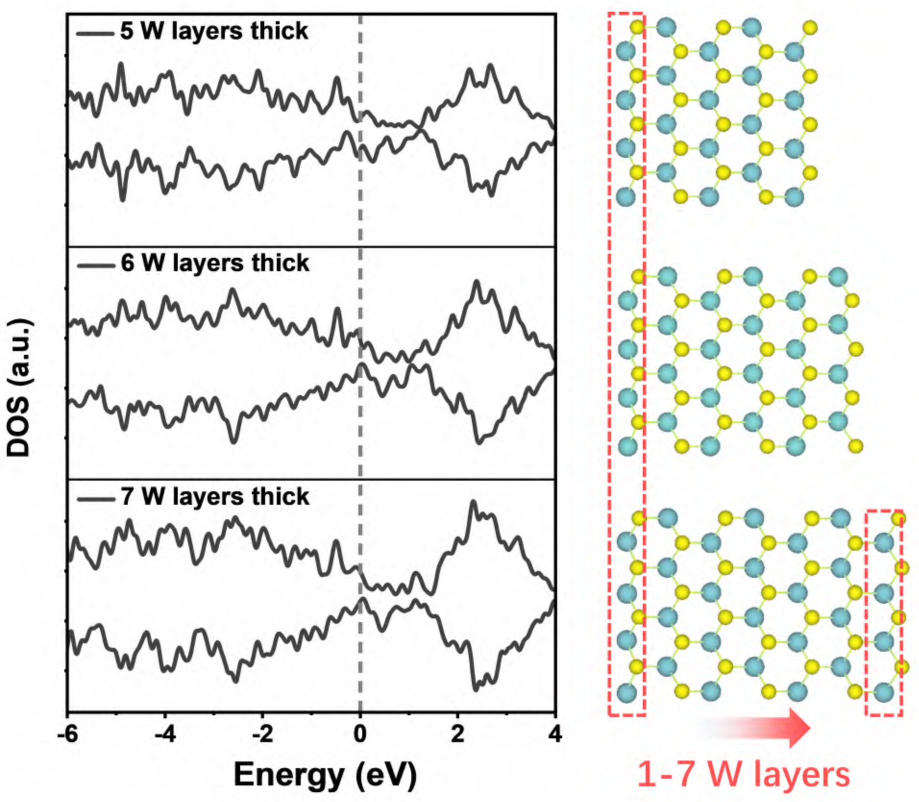

Fig. S4. Convergence test for 2H-WS_2 zigzag edge. The DOS of 2H-WS_2 zigzag edge with different W layers and corresponding structures. The DOS does not change significantly with the increase of the W layers suggesting that a thickness of 5 layers is sufficient for the calculation.

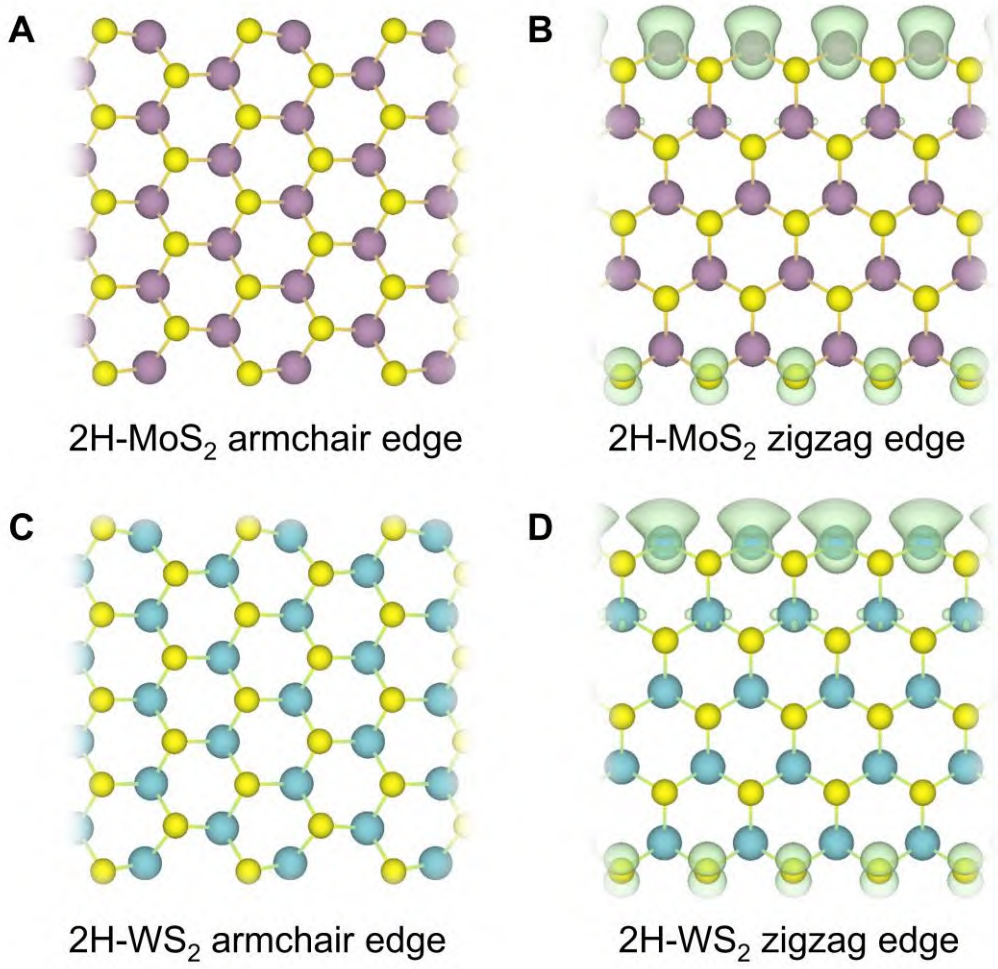

Fig. S5. Spin density distribution. The spin density distributions for (**A**) 2H-MoS_2 armchair edge; (**B**) 2H-MoS_2 zigzag edge; (**C**) 2H-WS_2 armchair edge; (**D**) 2H-WS_2 zigzag edge. The isosurface level is 0.006 e/$Å^3$.

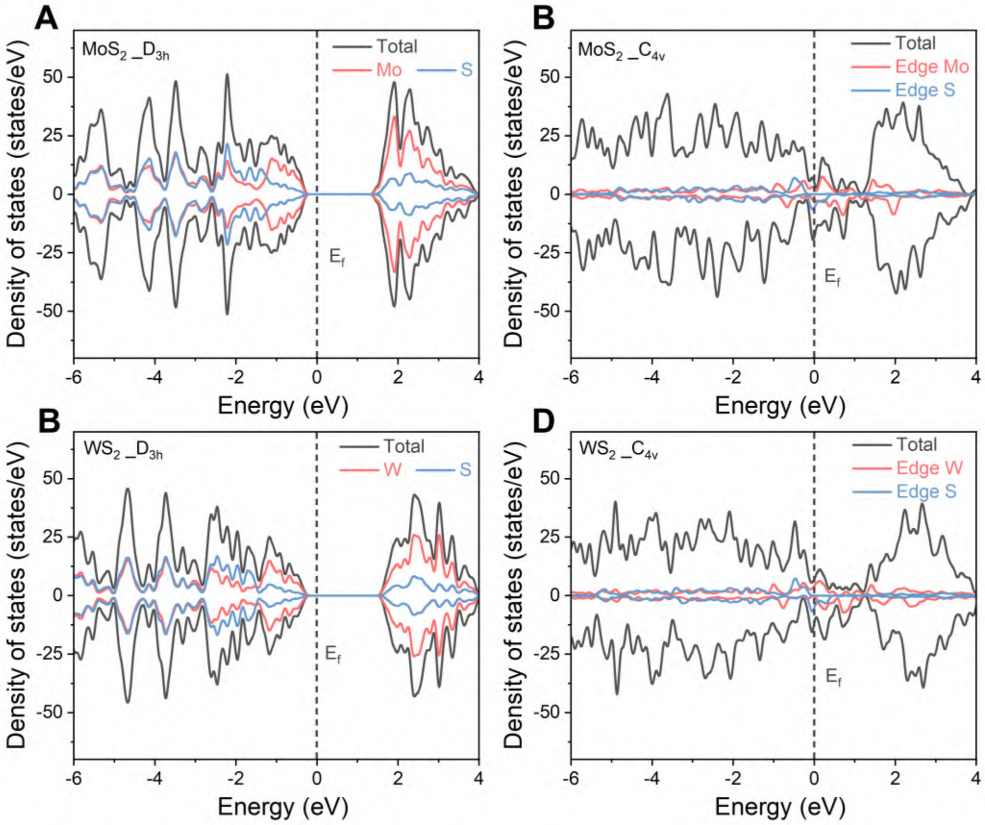

Fig. S6. Density of states comparison. The DOS for (**A**) 2H-MoS_2 basal plane; (**B**) 2H- MoS_2 zigzag edge; (**C**) 2H-WS_2 basal plane; (**D**) 2H- WS_2 zigzag edge.

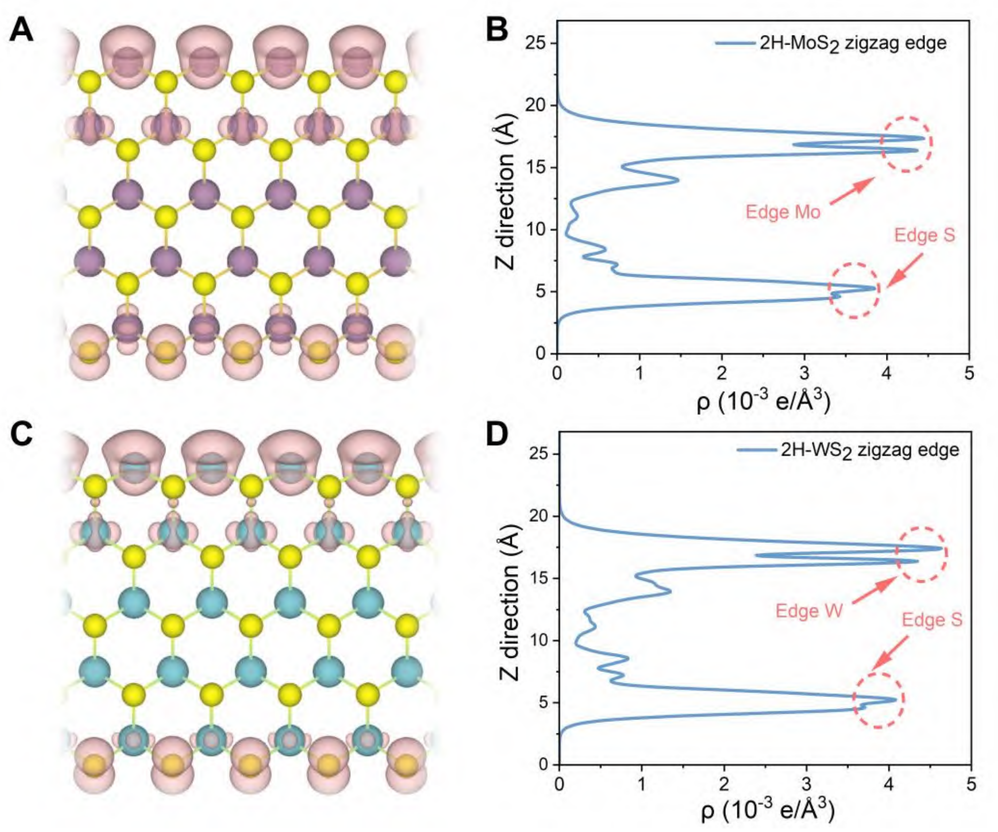

Fig. S7. Partial charge density near Fermi level. (**A**) The partial charge density near the Fermi energy level (-0.5 to 0.5 eV) of the 2H-MoS_2 zigzag edge and (**B**) the corresponding 1D planar-average partial charge density. (**C**) The partial charge density near the Fermi energy level (-0.5 to 0.5 eV) of the 2H-WS_2 zigzag edge and (**D**) the corresponding 1D planar-average partial charge density. The results indicate that the electronic states near the Fermi energy level are mainly contributed by Mo/W and S at the zigzag edges. The isosurface level is 0.01 e/Å^3.

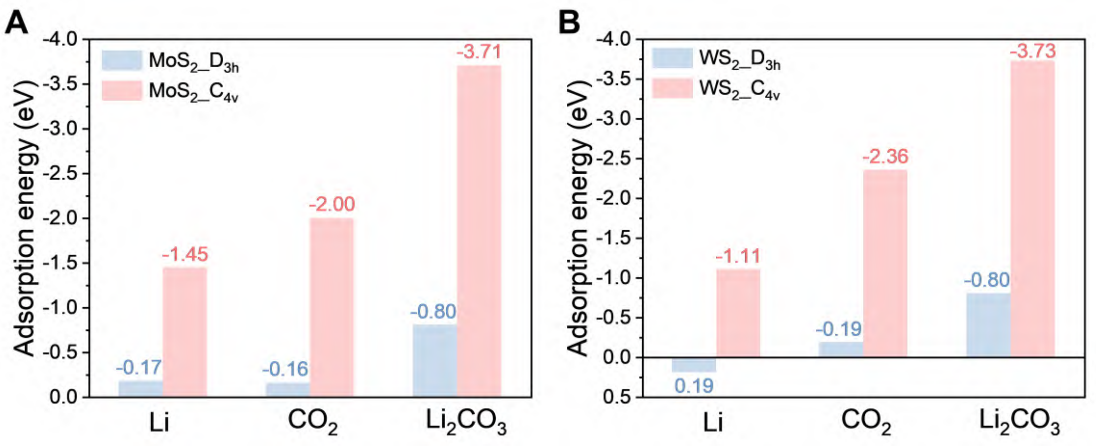

Fig. S8. Adsorption energy comparison. The adsorption energy of Li, CO_2 and Li_2CO_3 on (**A**) 2H-MoS_2, and (**B**) 2H-WS_2 basal plane and zigzag edge, respectively.

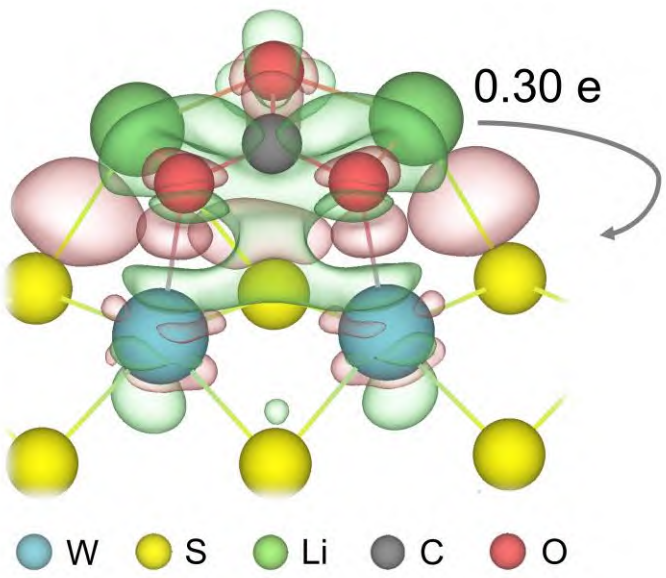

Fig. S9. Charge density difference upon Li_2CO_3 adsorption. Charge density difference analysis for Li_2CO_3 adsorption on WS_2_C_{4v}. The red and green isosurfaces represent regions of electron accumulation and depletion, respectively (isosurface value = 0.0035 e Å^{-3}).

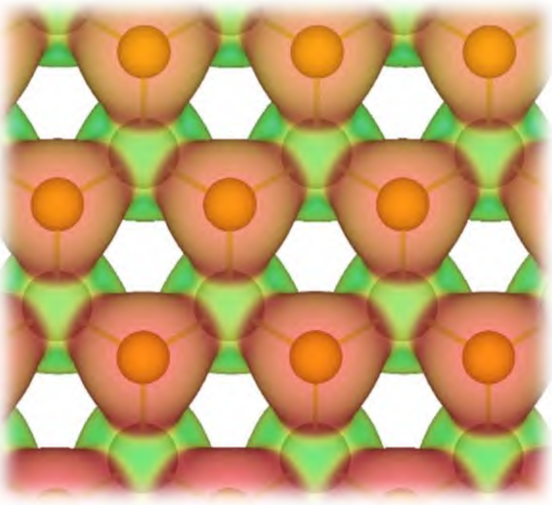

Fig. S10. Surface electrostatic potential. The surface electrostatic potential diagrams for 2H-WS_2 basal plane. The findings indicate that the absence of electrophilic sites on the basal plane cannot provide CO_2 adsorption sites.

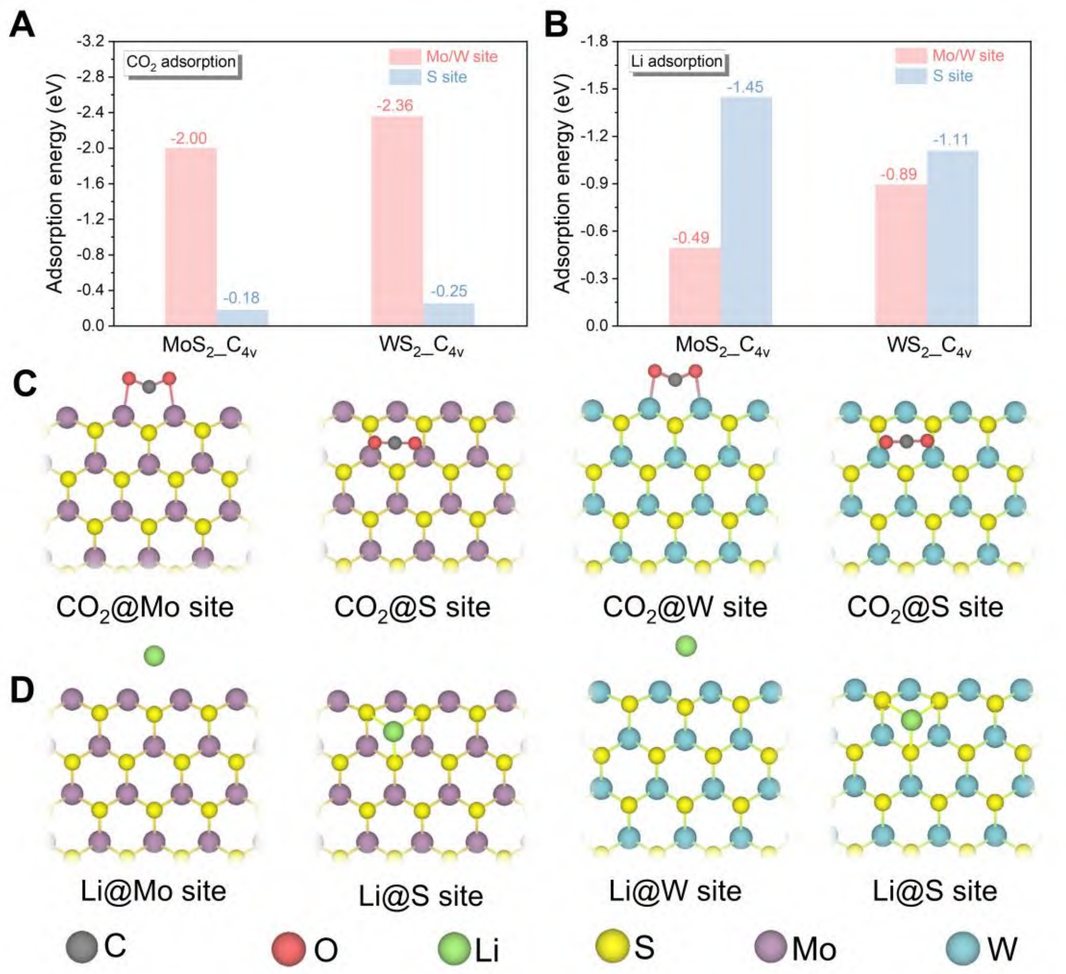

Fig. S11. Site-dependent adsorption energies. The adsorption energies of (**A**) CO_2 and (**B**) Li at Mo/W and S sites, respectively. (**C**, **D**) The pictures show corresponding adsorption structures.

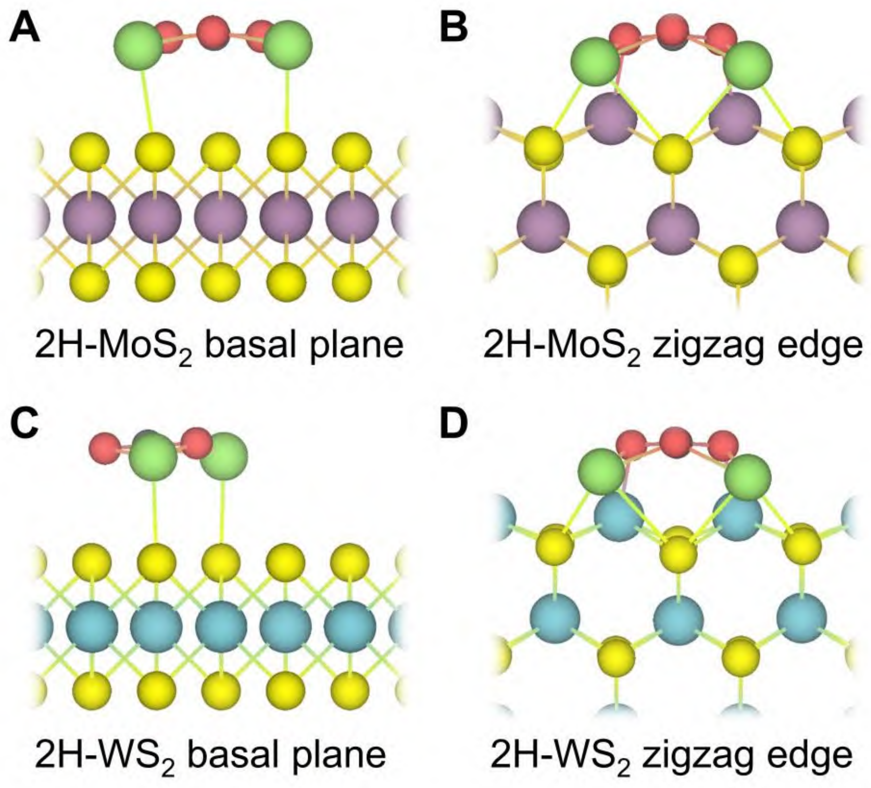

Fig. S12. Li_2CO_3 adsorption configurations. The adsorption configurations of Li_2CO_3 on (**A**) 2H-MoS_2 basal plane, (**B**) 2H-MoS_2 zigzag edge, (**C**) 2H-WS_2 basal plane and (**D**) 2H-WS_2 zigzag edge respectively.

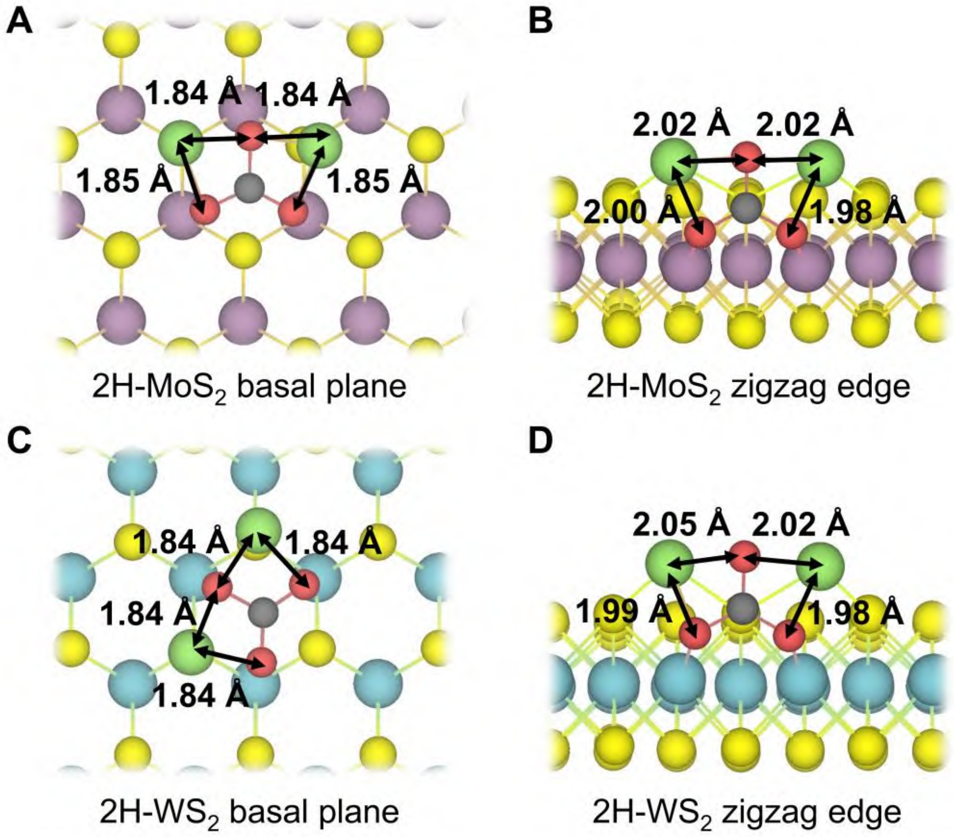

Fig. S13. Li-O bond lengths in adsorbed Li_2CO_3. The Li-O bond lengths of Li_2CO_3 adsorbed on (**A**) 2H-MoS_2 basal plane, (**B**) 2H-MoS_2 zigzag edge, (**C**) 2H-WS_2 basal plane and (**D**) 2H-WS_2 zigzag edge respectively.

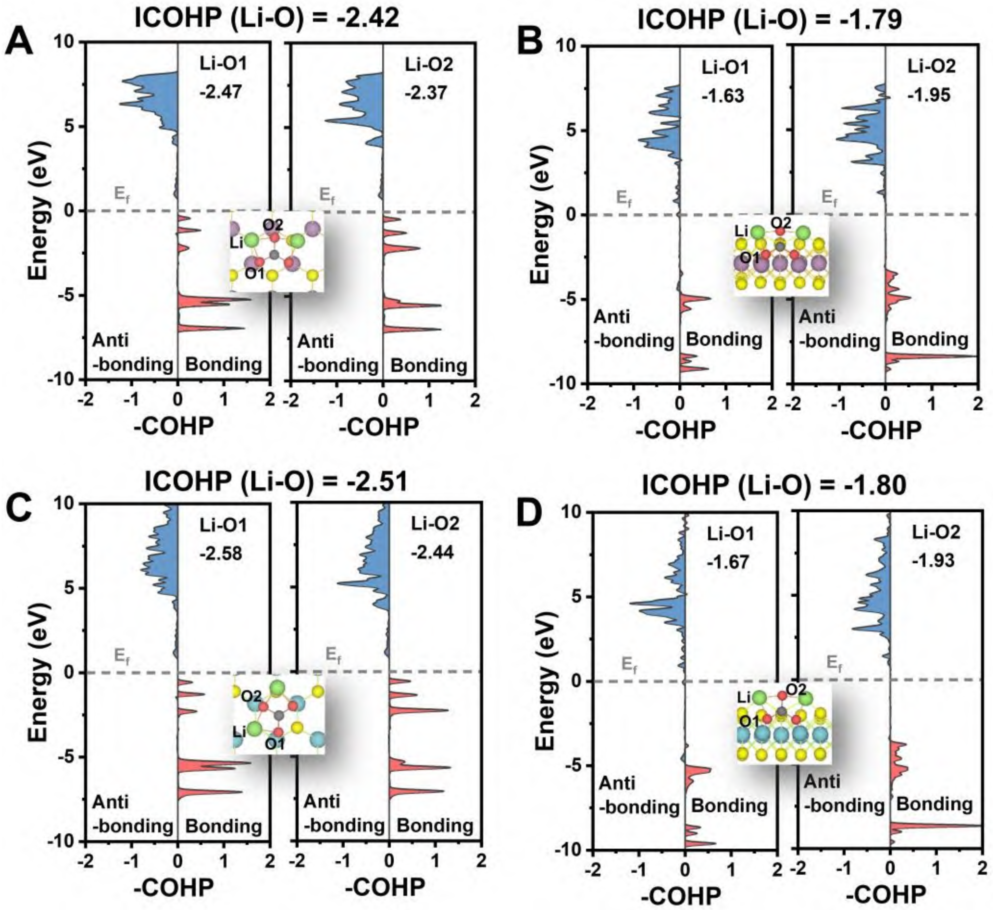

Fig. S14. ICOHP analysis of Li-O bond. The Integrated Crystal Occupation Hamiltonian Population (ICOHP) of Li-O bond of Li_2CO_3. (**A**) 2H-MoS_2 basal plane, (**B**) 2H-MoS_2 zigzag edge, (**C**) 2H-WS_2 basal plane and (**D**) 2H-WS_2 zigzag edge respectively.

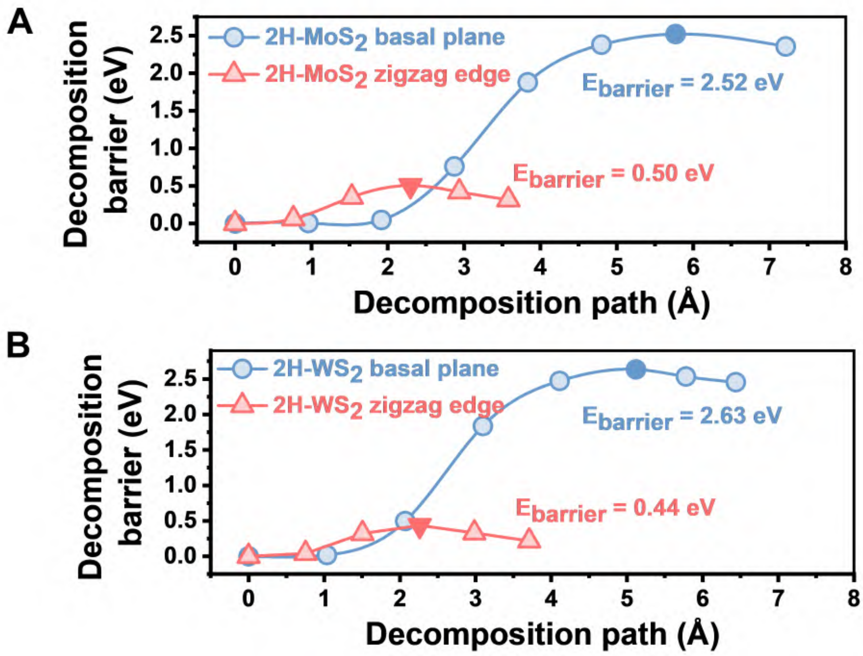

Fig. S15. Li_2CO_3 decomposition barriers. (**A**) Decomposition barriers of Li_2CO_3 on 2H-MoS_2 basal plane and zigzag edge. (**B**) Decomposition barriers of Li_2CO_3 on 2H-WS_2 basal plane and zigzag edge. The decomposition barriers of Li_2CO_3 at the zigzag edges are significantly lower than at the basal plane, indicating that the zigzag edges are capable of functioning as active sites for the reaction.

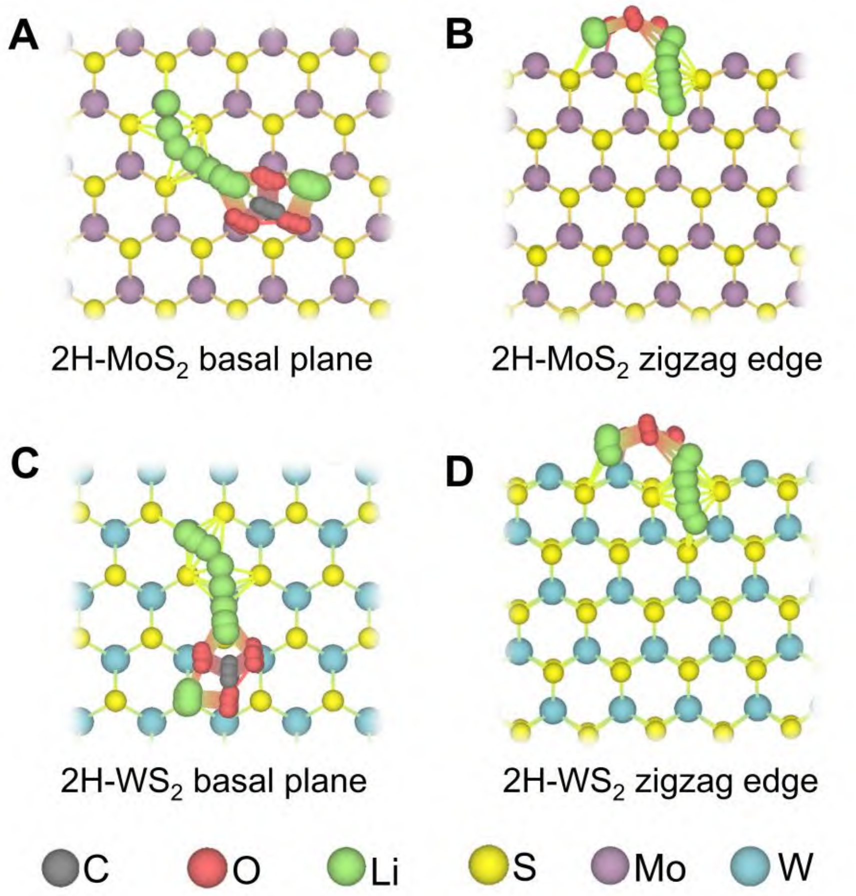

Fig. S16. Li_2CO_3 decomposition paths. Decomposition paths of Li_2CO_3 on (**A**) 2H-MoS_2 basal plane, (**B**) 2H-MoS_2 zigzag edge, (**C**) 2H-WS_2 basal plane, and (**D**) 2H-WS_2 zigzag edge.

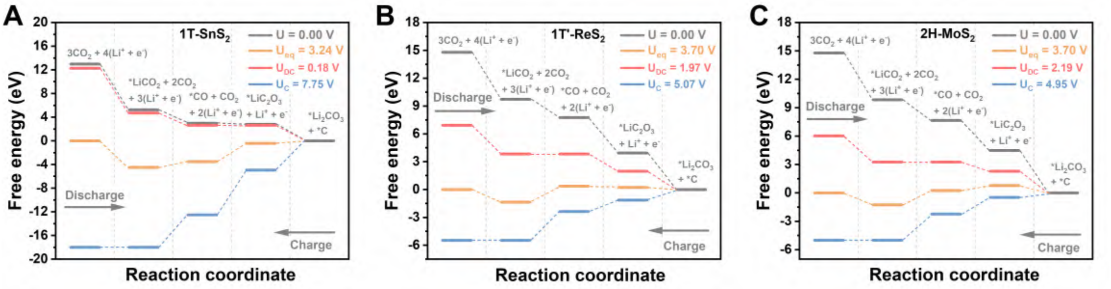

Fig. S17. Calculated discharge/charge and equilibrium potential. Calculated discharge/charge and equilibrium potential for (**A**) 1T-SnS_2, (**B**) 1T′-ReS_2, and (**C**) 2H-MoS_2.

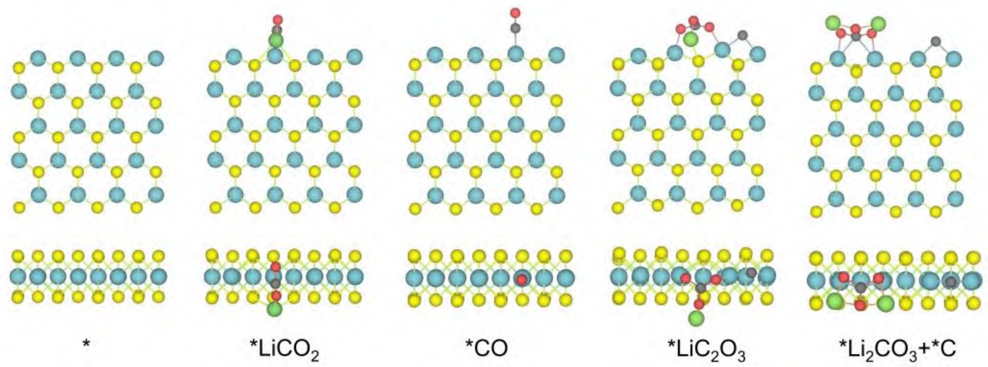

Fig. S18. Optimized geometric structures of key intermediates. The side and top views of optimized geometric structures of key intermediates on 2H-WS_2 along reaction pathway.

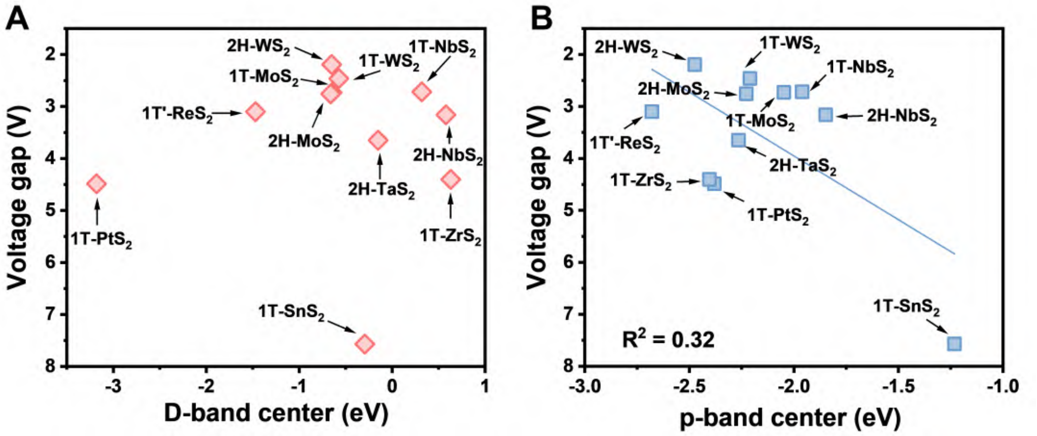

Fig. S19. Relationship between voltage gap and band centers. The Relationship between the voltage gap and (**A**) *d*-band center of metal, and (**B**) *p*-band center of sulfur in 2D transition metal dichalcogenides (TMDs) systems.

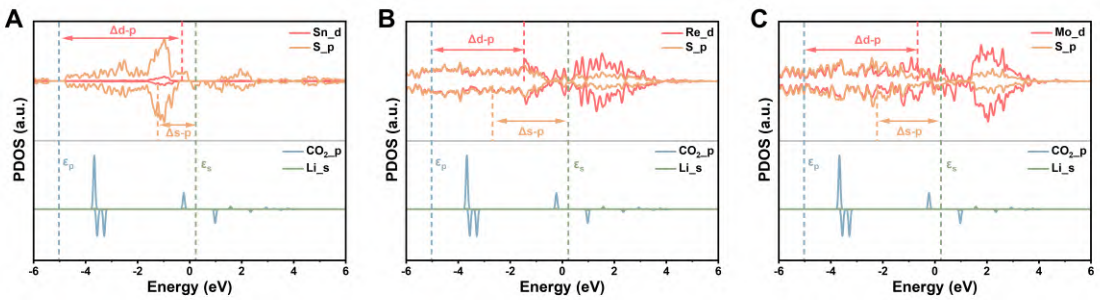

Fig. S20. DOS of TMDs and $LiCO_2$ intermediate. The DOS of (**A**) 1T-SnS_2, (**B**) 1T′-ReS_2, (**C**) 2H-MoS_2, and $LiCO_2$ intermediates. The energy gap (Δd-p) between metal and CO_2, and the energy gap (Δs-p) between Li and S are plotted.

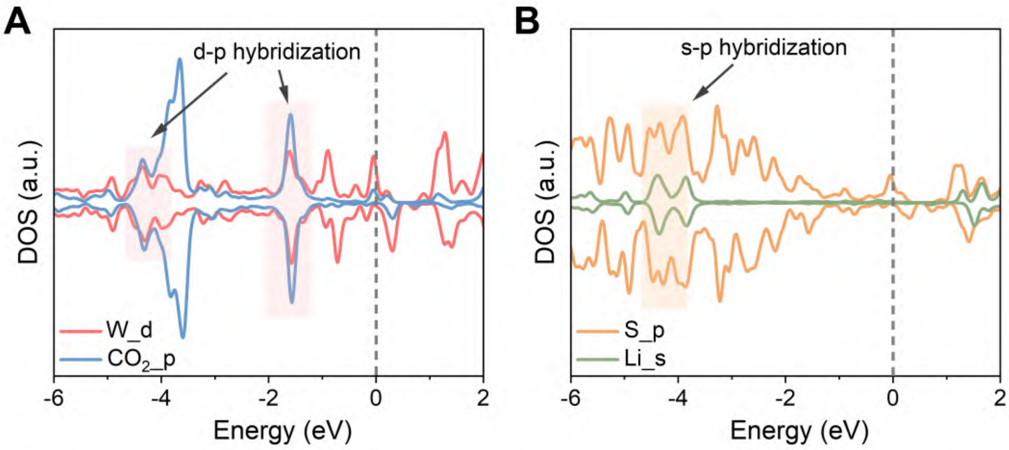

Fig. S21. d-p and s-p hybridization after $LiCO_2$ adsorption. The DOS at the active site after $LiCO_2$ intermediate adsorbed on 2H-WS_2. (**A**) *d-p* hybridization and (**B**) *s-p* hybridization (The DOS for the Li_s is magnified by a factor of 10 for better visibility).

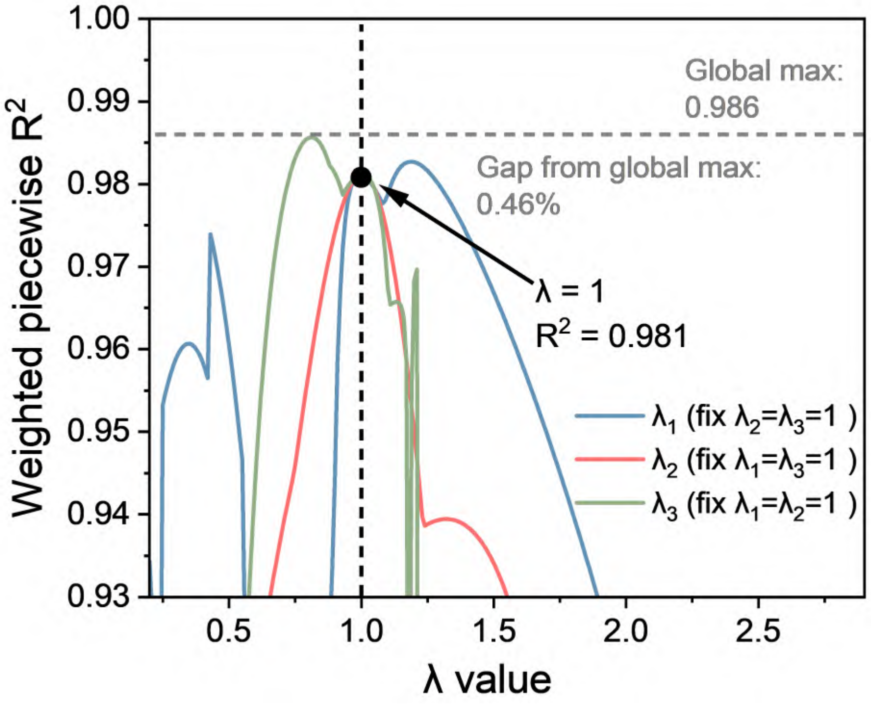

Fig. S22. Sensitivity analysis of descriptor R^2. Sensitivity of R^2 to λ perturbation. λ scanned from 0.2 to 2.8 in steps of 0.01.

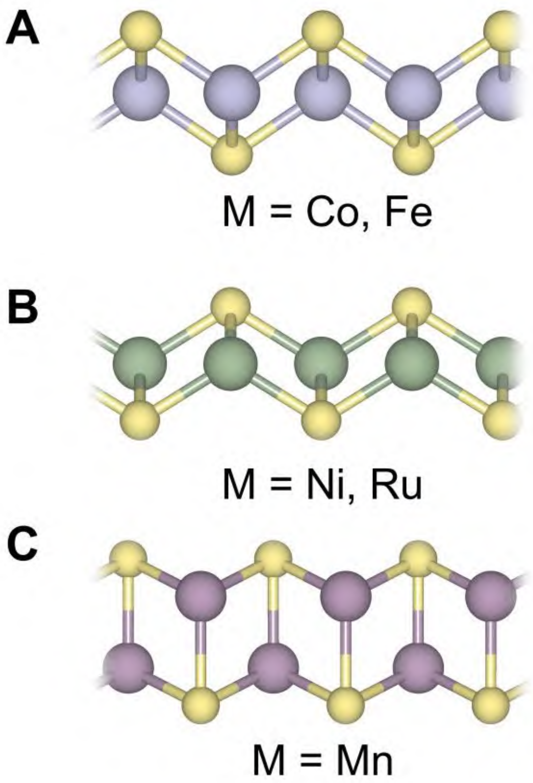

Fig. S23. Edge structures of other sulfides. Edge structures of (a) Co/FeS, (b) Ni/RuS and (c) MnS.

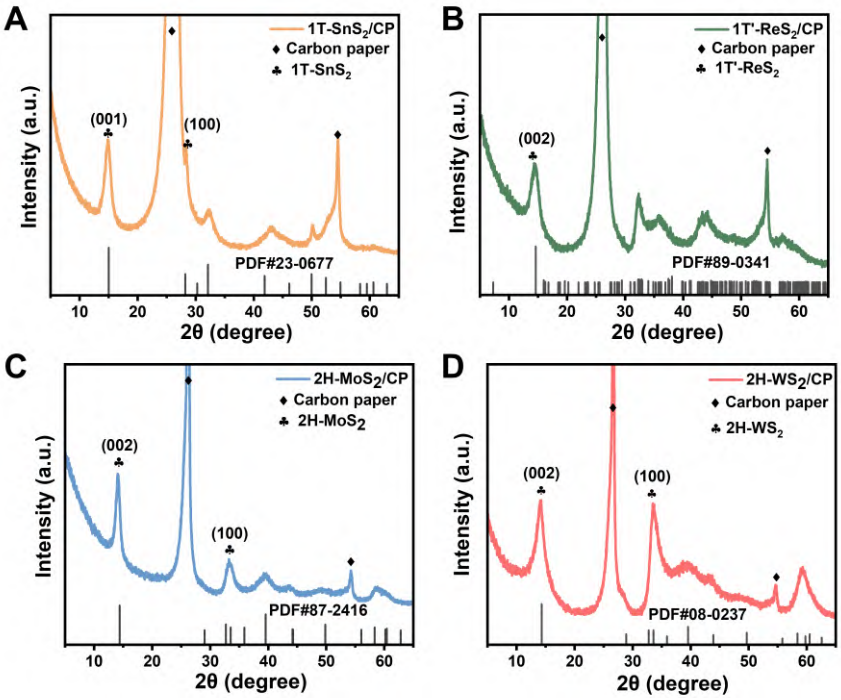

Fig. S24. XRD patterns of TMDs/CP electrodes. X-ray diffraction (XRD) patterns of (**A**)1T-SnS_2/carbon paper (CP), (**B**) 1T′-ReS_2/CP, (**C**) 2H-MoS_2/CP and (**D**) 2H-WS_2/CP.

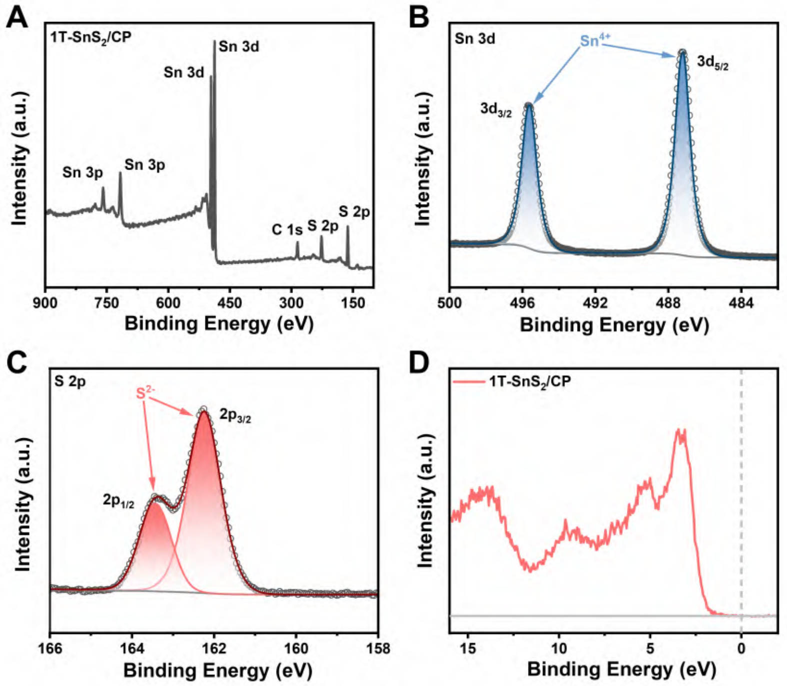

Fig. S25. XPS characterization of 1T-SnS_2/CP. (**A**) X-ray photoelectron spectroscopy (XPS) spectra, (**B**) Sn 3*d* spectra, (**C**) S 2*p* spectra and (**D**) valence band (VB) spectra of 1T-SnS_2/CP.

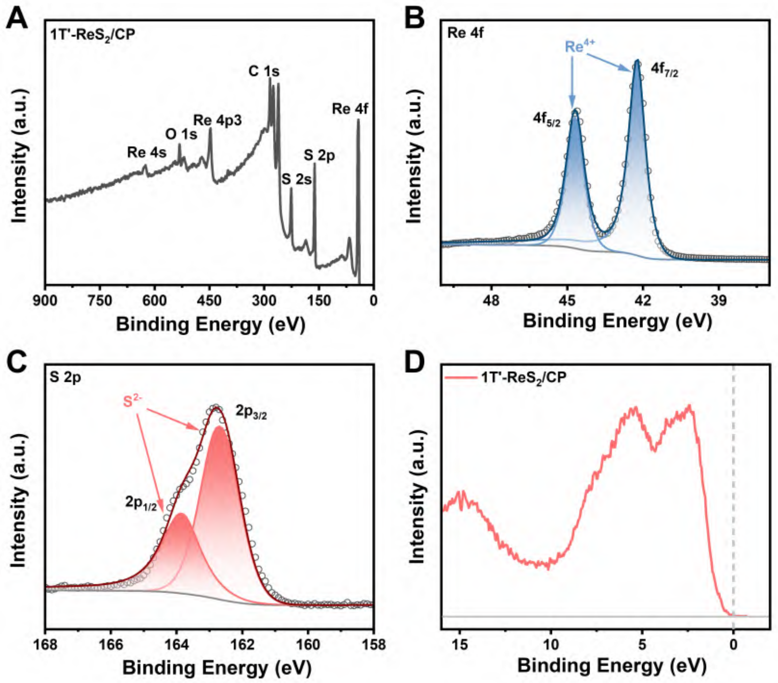

Fig. S26. XPS characterization of 1T′-ReS$_2$/CP. (**A**) XPS spectra, (**B**) Re 4*f* spectra, (**C**) S 2*p* spectra and (**D**) VB spectra of 1T′-ReS$_2$/CP.

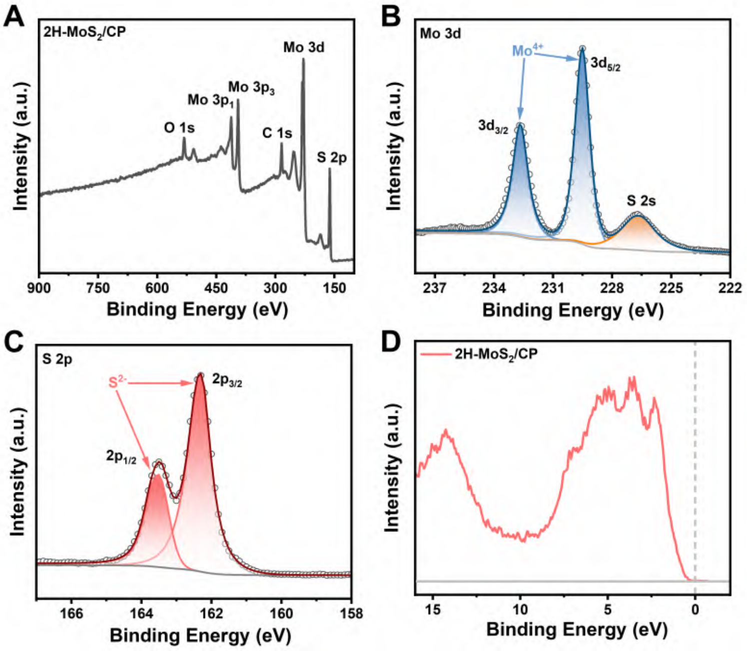

Fig. S27. XPS characterization of 2H-MoS$_2$/CP. (**A**) XPS spectra, (**B**) Mo 3*d* spectra, (**C**) S 2*p* spectra and (**D**) VB spectra of 2H-MoS$_2$/CP.

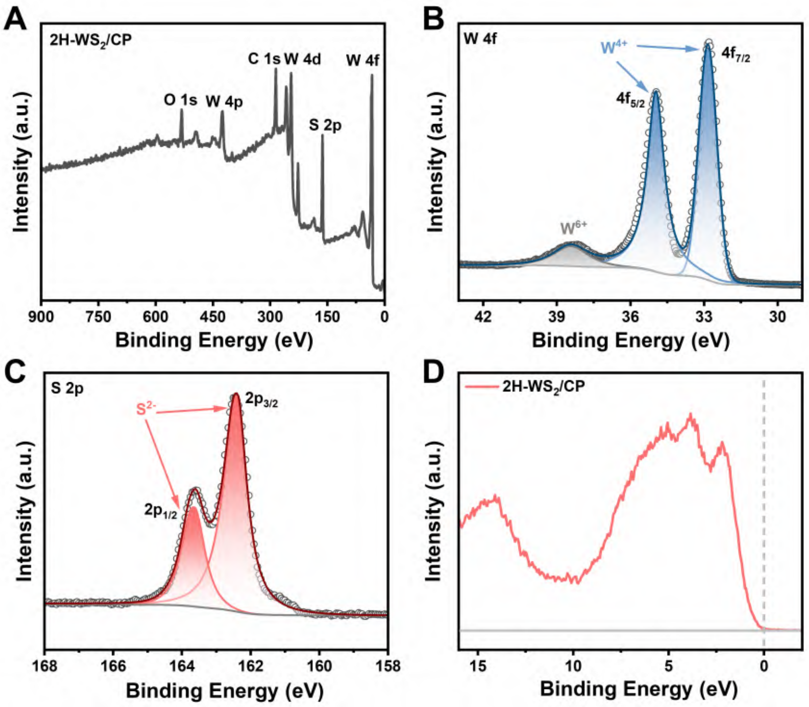

Fig. S28. XPS characterization of 2H-WS$_2$/CP. (**A**) XPS spectra, (**B**) W 4*f* spectra, (**C**) S 2*p* spectra and (**D**) VB spectra of 2H-WS$_2$/CP.

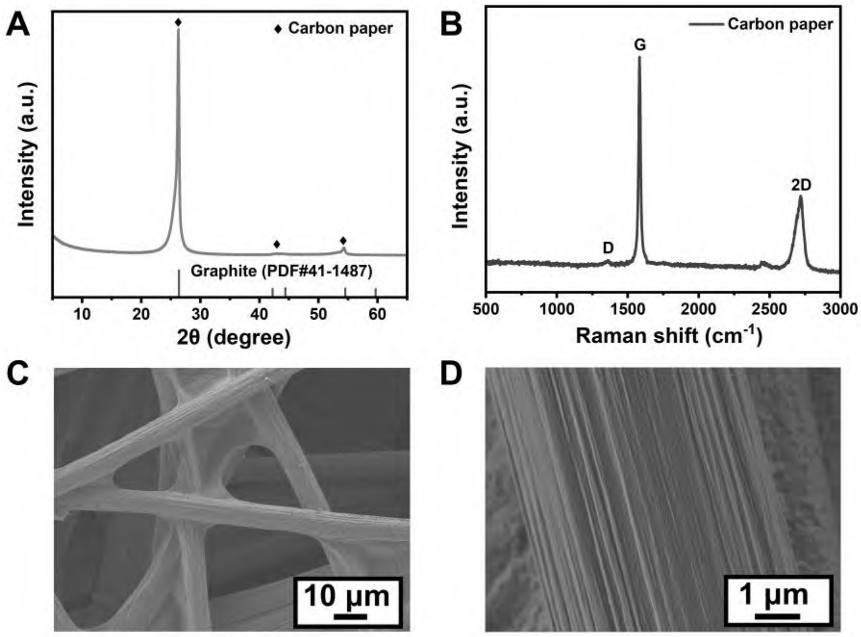

Fig. S29. Characterization of carbon paper (CP) substrate. (**A**) XRD pattern, (**B**) Raman spectrum and (**C**, **D**) scanning electron microscope (SEM) images of CP. The CP with a rough surface and a high degree of graphitization are capable of acting as a skeleton for the growth of 2D TMDs.

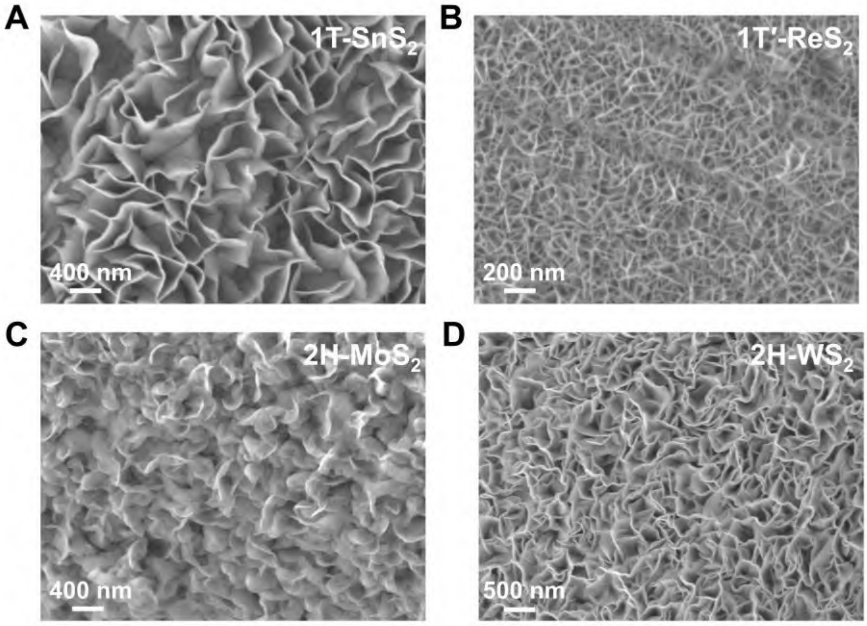

Fig. S30. SEM images of TMDs materials. SEM images of (**A**) 1T-SnS_2, (**B**) 1T′-ReS_2, (**C**) 2H-MoS_2 and (**D**) 2H-WS_2.

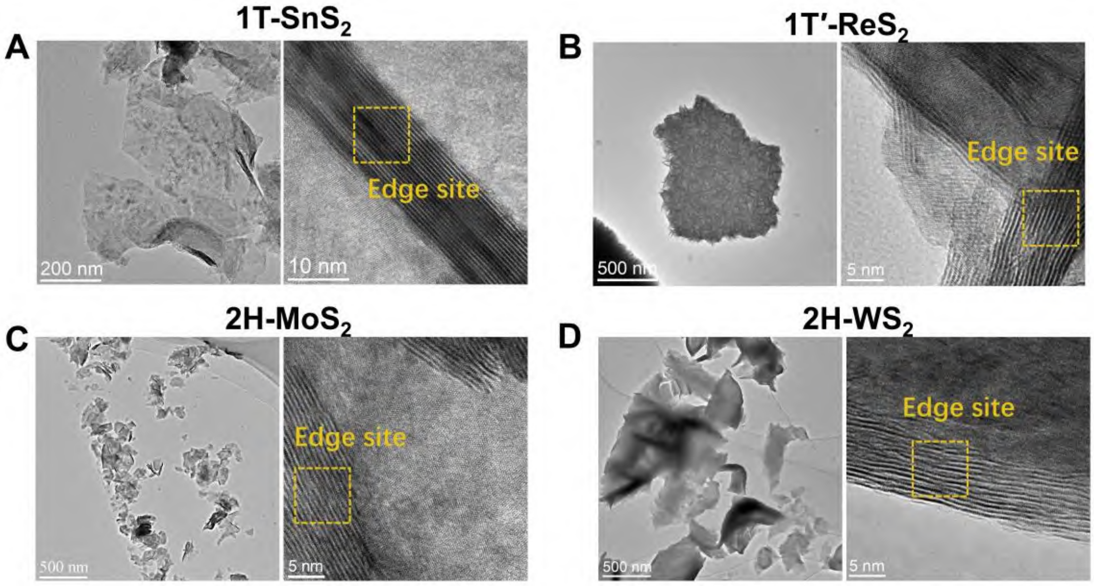

Fig. S31. TEM and HRTEM images of TMDs. High-resolution transmission electron microscopy (HRTEM) images of (**A**)1T-SnS_2, (**B**) 1T′-ReS_2, (**C**) 2H-MoS_2 and (**D**) 2H-WS_2.

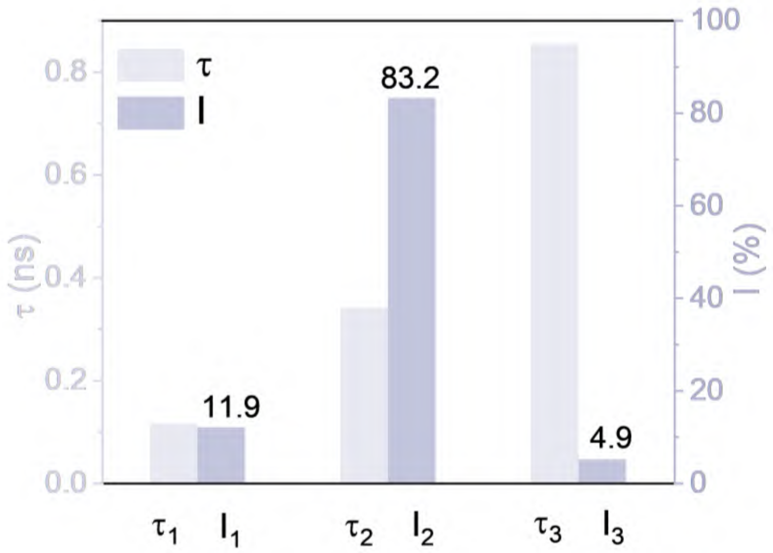

Fig. S32. Positron lifetime parameters. Positron lifetime parameters of WS_2_D_{3h}.

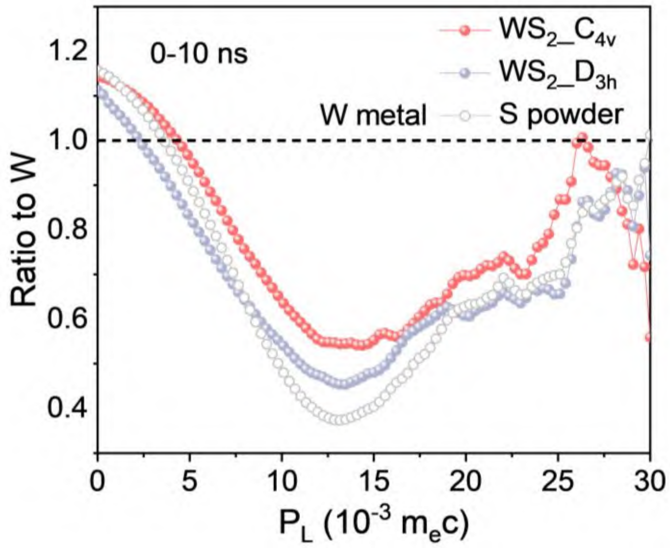

Fig. S33. Doppler broadening ratio curve. Ratio curve of Doppler broadening (derived from positron annihilation age-momentum correlation (AMOC) spectroscopy in the positron age region of 0-10 ns) of WS_2_C_{4v} and WS_2_D_{3h} samples with respect to that of pure W metal.

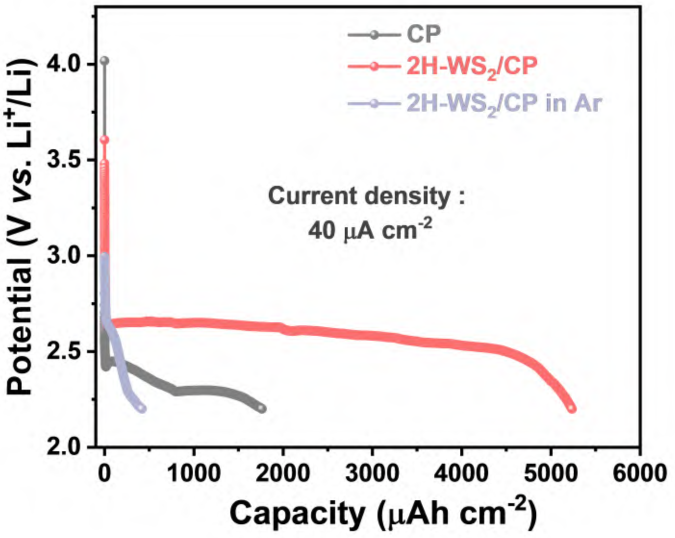

Fig. S34. Full discharge curves. Fully discharging curves at 40 μA cm^{-2} for CP and 2H-WS_2/CP.

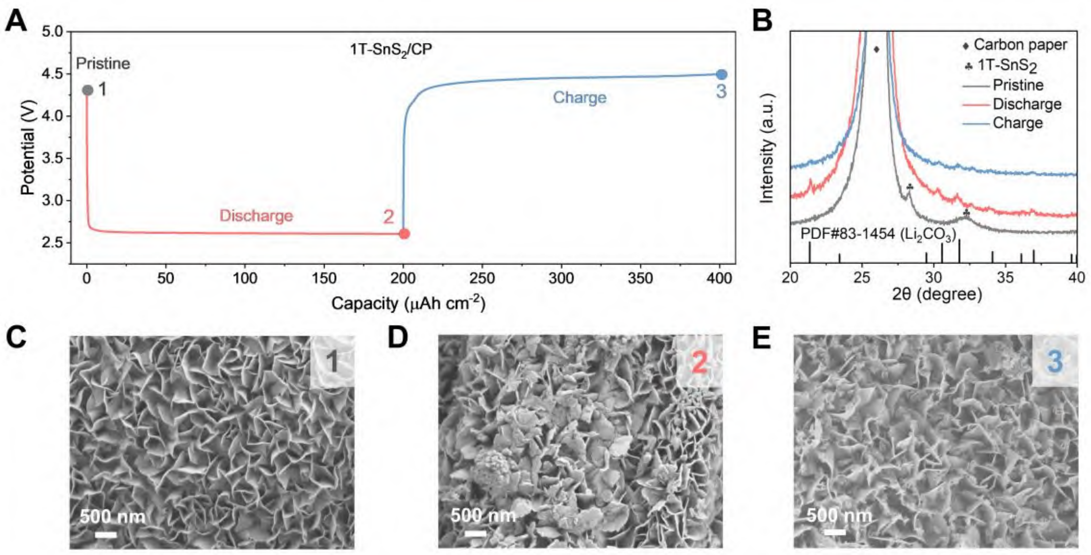

Fig. S35. Ex-situ XRD and SEM of 1T-SnS_2/CP during cycling. (**A**) Galvanostatic discharge-charge (GDC) profiles of the 1T-SnS_2/CP at 20 μA cm^{-2} with a capacity limit of 200 μAh cm^{-2}. The labels 1, 2 and 3 represent the pristine state, electrochemical states after discharging with 200, and charging with 200 μAh cm^{-2}, respectively. (**B**) XRD patterns, (**C-E**) SEM images under pristine, discharged and charged states corresponding to (**A**).

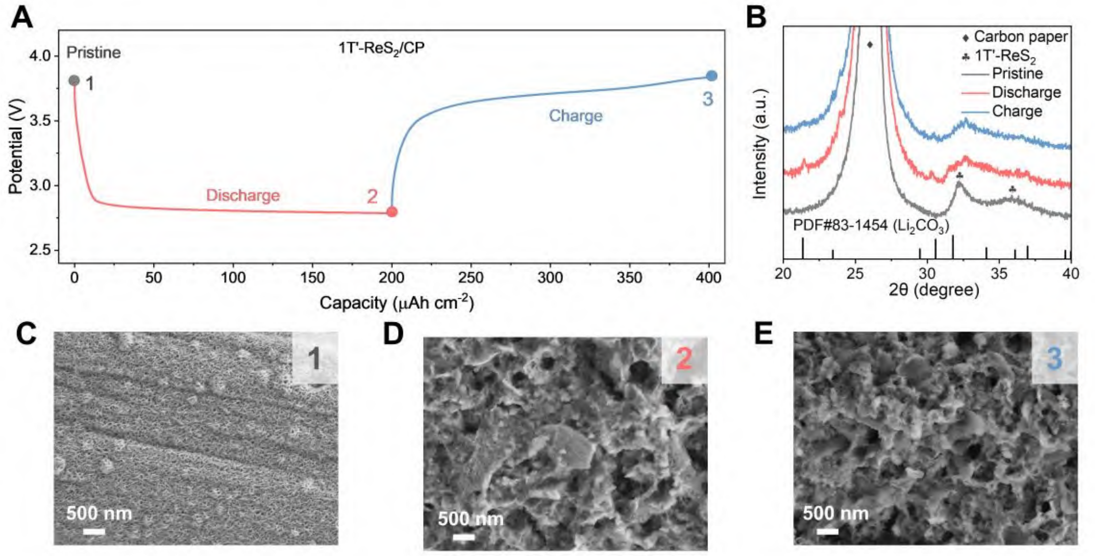

Fig. S36. Ex-situ XRD and SEM of 1T′-ReS2 during cycling. (**A**) GDC profiles of the 1T′-ReS_2 at 20 μA cm^{-2} with a capacity limit of 200 μAh cm^{-2}. The labels 1, 2 and 3 represent the pristine state, electrochemical states after discharging with 200, and charging with 200 μAh cm^{-2}, respectively. (**B**) XRD patterns, (**C-E**) SEM images under pristine, discharged and charged states corresponding to (**A**).

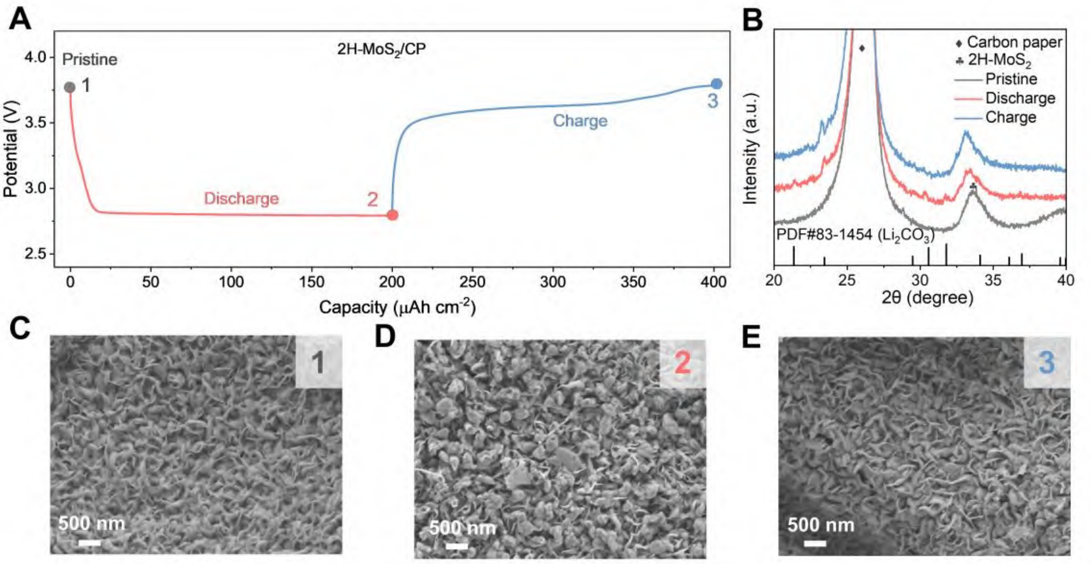

Fig. S37. **Ex-situ XRD and SEM of 2H-MoS$_2$ during cycling.** (**A**) GDC profiles of the 2H-MoS$_2$ at 20 μA cm^{-2} with a capacity limit of 200 μAh cm^{-2}. The labels 1, 2 and 3 represent the pristine state, electrochemical states after discharging with 200, and charging with 200 μAh cm^{-2}, respectively. (**B**) XRD patterns, (**C-E**) SEM images under pristine, discharged and charged states corresponding to (a).

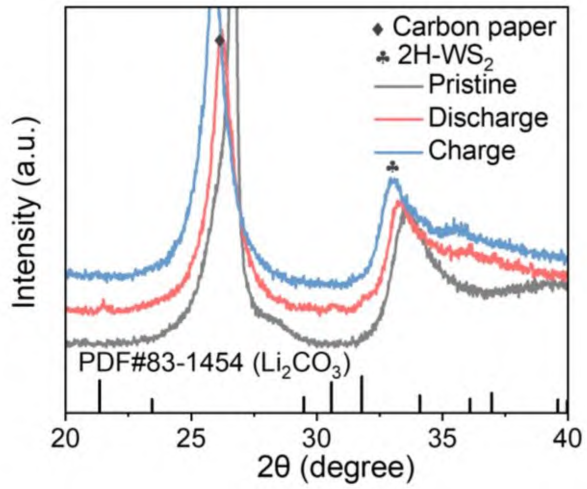

Fig. S38. XRD of 2H-WS_2 electrode at different states. XRD patterns of the 2H-WS_2 electrode at different states: pristine, discharged, and charged, corresponding to its GDC profile measured at 20 μA cm^{-2} with a capacity limit of 200 μAh cm^{-2}.

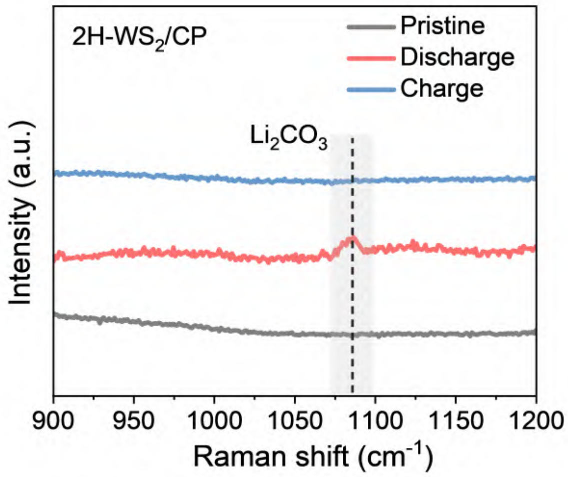

Fig. S39. Raman spectra of Li_2CO_3. Raman spectra of Li_2CO_3 on 2H-WS_2/CP under pristine, discharged and charged states, measured with a limited capacity of 200 μAh cm^{-2} at a current density of 20 μA cm^{-2}.

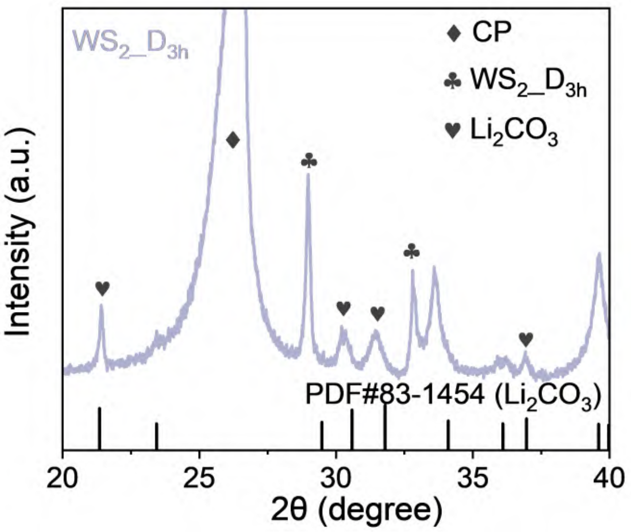

Fig. S40. XRD after deep discharge. XRD patterns of WS_2_D_{3h} after deep discharge at 20 μA cm^{-2} with a capacity limit of 3000 μAh cm^{-2}.

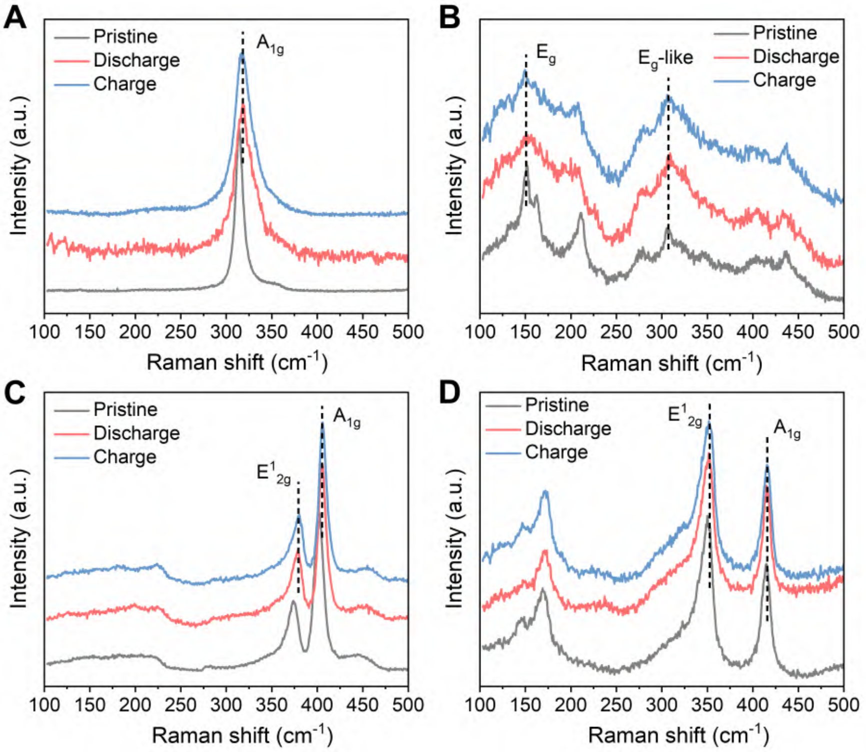

Fig. S41. Raman spectra of various TMDs/CP electrodes. Raman spectra of (**A**)1T-SnS_2/CP, (**B**) 1T′-ReS_2/CP, (**C**) 2H-MoS_2/CP and (**D**) 2H-WS_2/CP under pristine, discharged and charged states, measured with a limited capacity of 200 μAh cm^{-2} at a current density of 20 μA cm^{-2}.

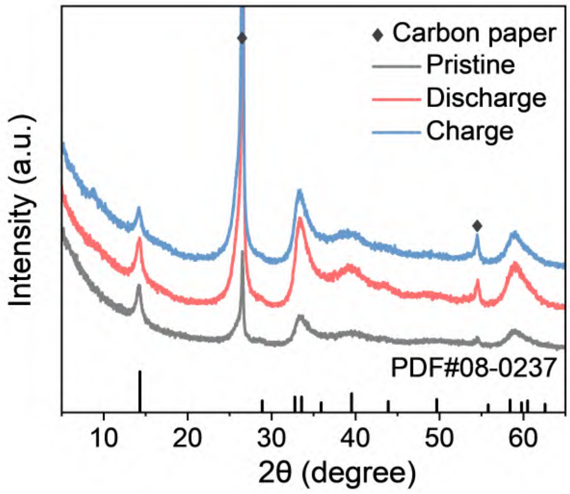

Fig. S42. XRD of 2H-WS_2/CP at limited capacity. XRD pattern of 2H-WS_2/CP under pristine, discharged and charged states, measured with a limited capacity of 100 μAh cm^{-2} at a current density of 20 μA cm^{-2}.

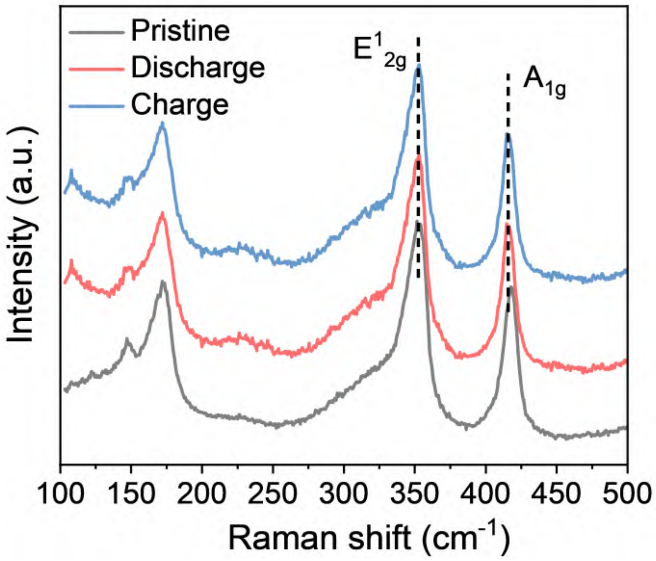

Fig. S43. **Raman of 2H-WS_2/CP at limited capacity.** Raman spectra of 2H-WS_2/CP under pristine, discharged and charged states, measured with a limited capacity of 100 μAh cm^{-2} at a current density of 20 μA cm^{-2}.

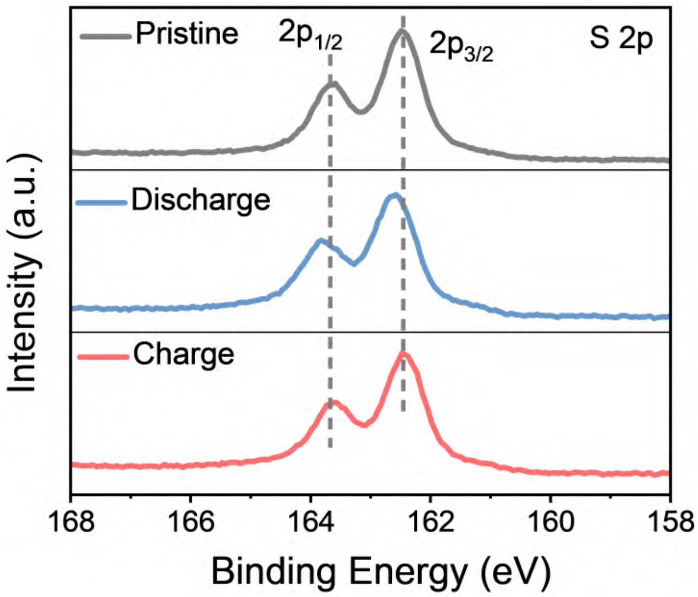

Fig. S44. S 2*p* X-ray photoelectron spectroscopy (XPS) spectra. S 2*p* spectra of 2H-WS_2/CP under pristine, discharged and charged states.

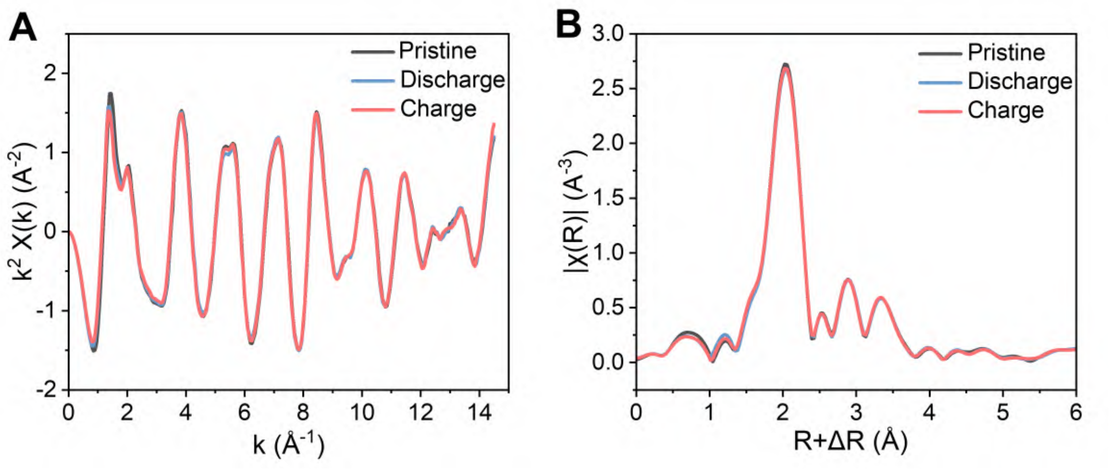

Fig. S45. EXAFS analysis of W L_3-edge. (**A**) k^2-weighted Extended X-ray absorption fine structure (EXAFS) oscillations and (**B**) Fourier-transformed R-space spectra of the W L_3-edge under pristine, discharged and charged states.

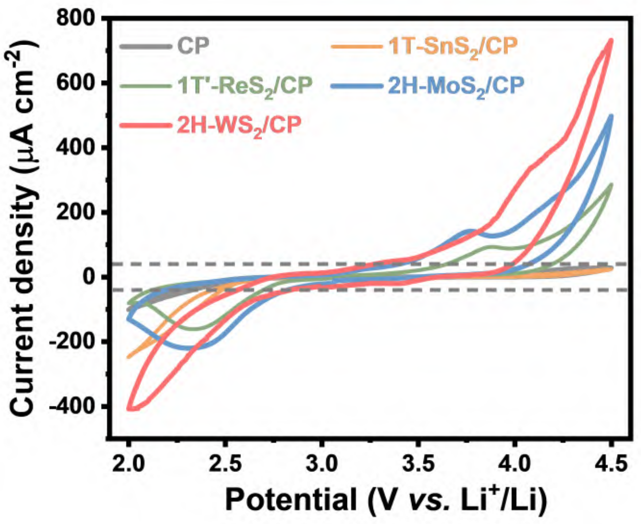

Fig. S46. Cyclic voltammetry (CV) curves. CV curves of CP, 1T-SnS_2/CP, 1T′-ReS_2/CP, 2H-MoS_2/CP, and 2H-WS_2/CP electrodes.

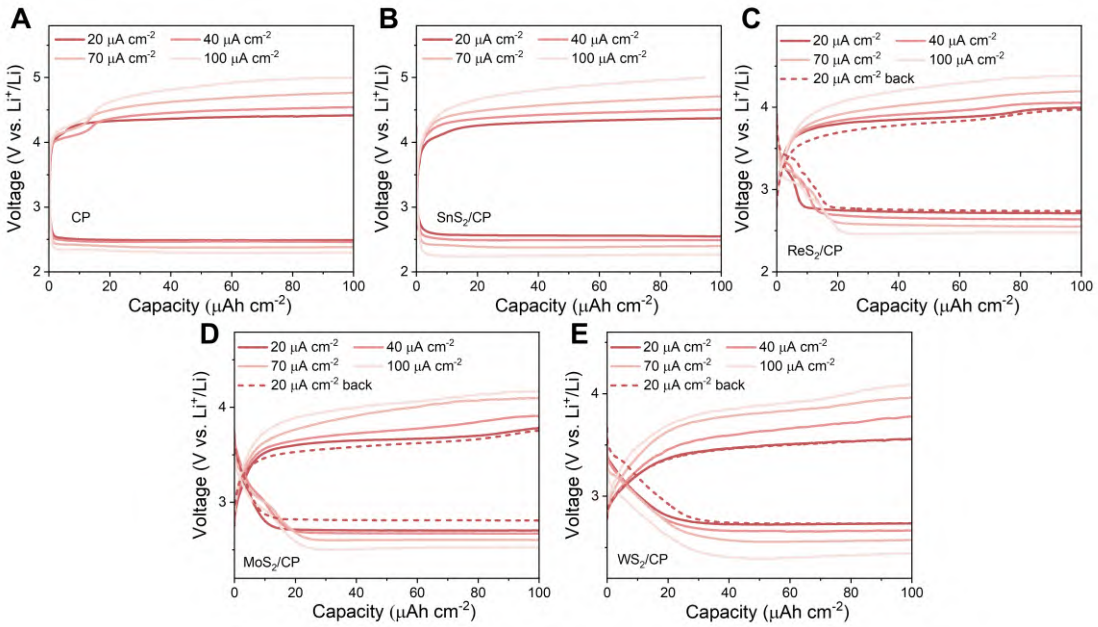

Fig. S47. Rate performance. GDC profiles of a limited capacity 100 μAh cm^{-2} at different current densities for (**A**) CP, (**B**)1T-SnS_2/CP, (**C**) 1T′-ReS_2/CP, (**D**) 2H-MoS_2/CP and (**E**) 2H-WS_2/CP.

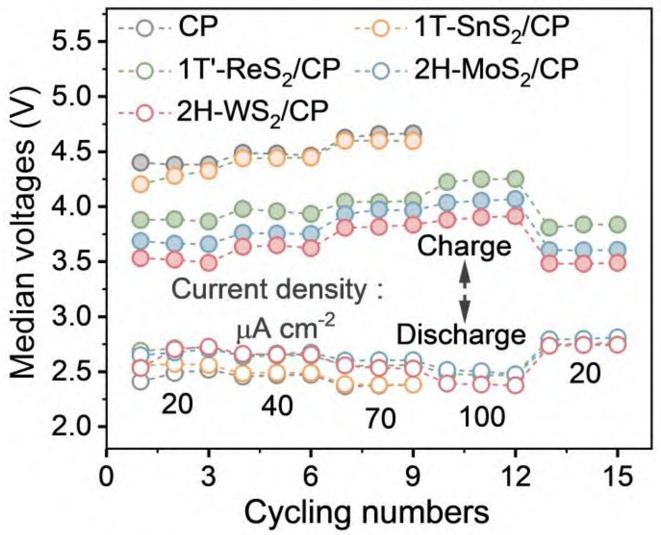

Fig. S48. Median voltage at different current densities. Discharge and charge median voltage at different current densities for CP, 1T-SnS_2/CP, 1T′-ReS_2/CP, 2H-MoS_2/CP, and 2H-WS_2/CP electrodes, respectively.

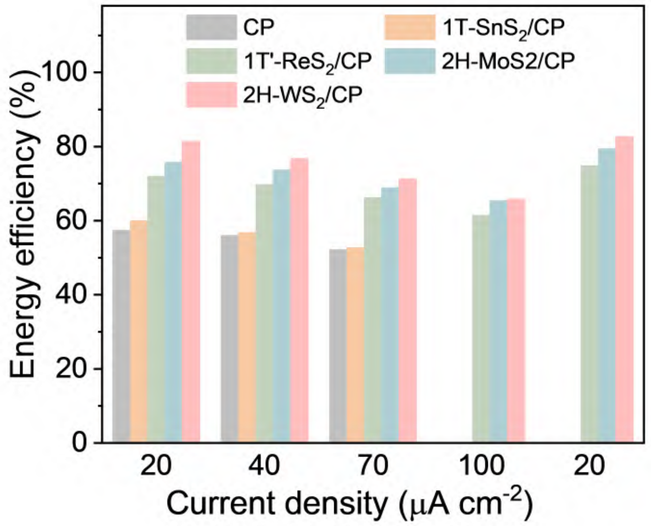

Fig. S49. Energy efficiency at different current densities. Energy efficiencies at different current densities for CP, 1T-SnS_2/CP, 1T′-ReS_2/CP, 2H-MoS_2/CP, and 2H-WS_2/CP electrodes, respectively.

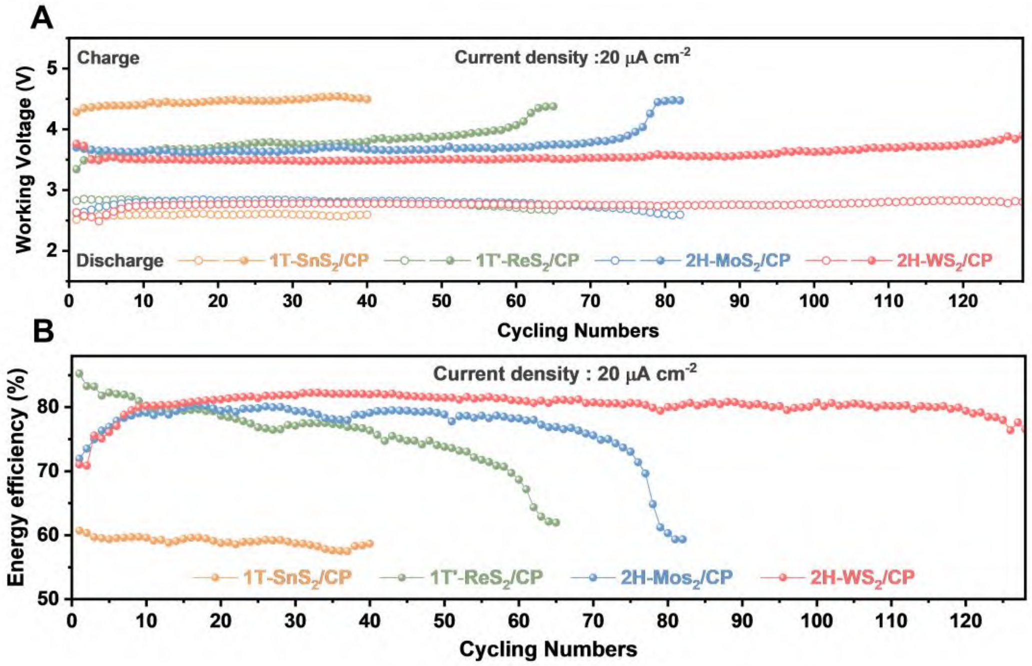

Fig. S50. Cycling performance at 20 μA cm^{-2}. Cycling performance of 1T-SnS_2/CP, 1T′-ReS_2/CP, 2H-MoS_2/CP, and 2H-WS_2/CP electrodes. (**A**) median voltage and (**B**) energy efficiency at 20 μA cm^{-2}.

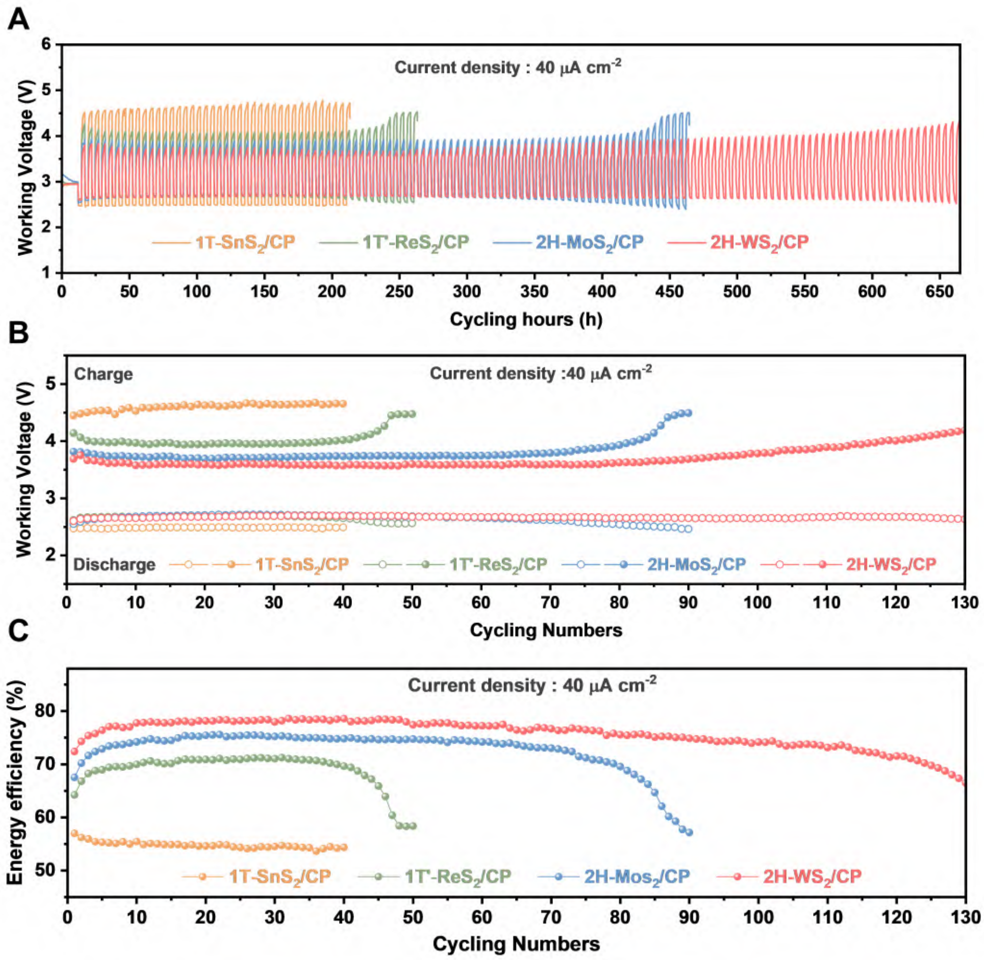

Fig. S51. Cycling performance at 40 μA cm^{-2}. Cycling performance of 1T-SnS_2/CP, 1T′-ReS_2/CP, 2H-MoS_2/CP, and 2H-WS_2/CP electrodes. (**A**) Time-voltage curves, (**B**) median voltage, and (**C**) energy efficiency at 40 μA cm^{-2}.

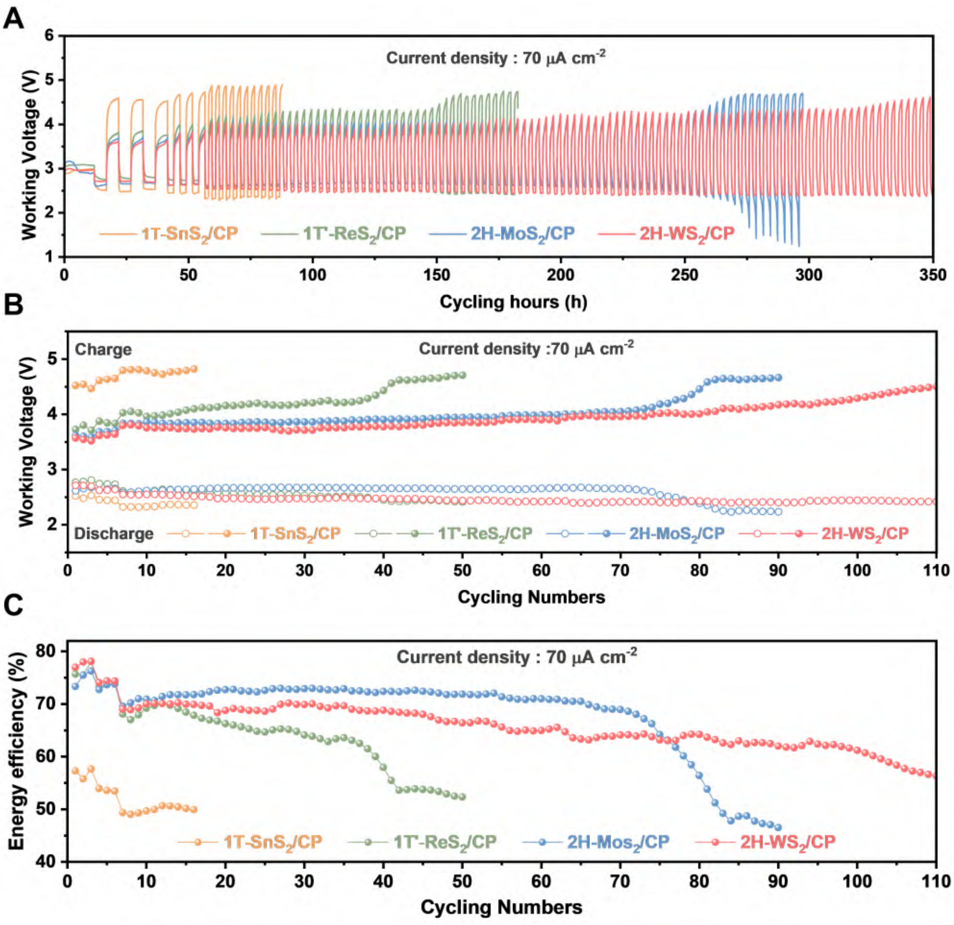

Fig. S52. Cycling performance at 70 μA cm^{-2}. Cycling performance of 1T-SnS_2/CP, 1T′-ReS_2/CP, 2H-MoS_2/CP, and 2H-WS_2/CP electrodes. (**A**) Time-voltage curves, (**B**) median voltage, and (**C**) energy efficiency at 70 μA cm^{-2}.

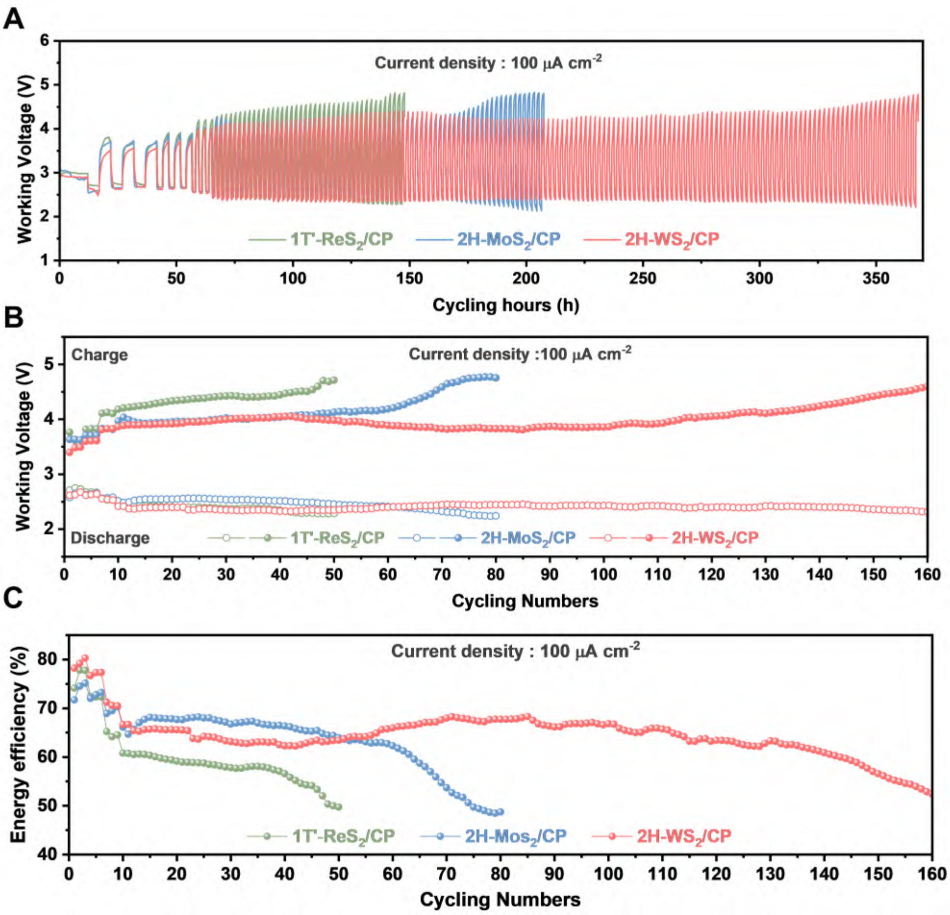

Fig. S53. Cycling performance at 100 μA cm^{-2}. Cycling performance of 1T-SnS_2/CP, 1T′-ReS_2/CP, 2H-MoS_2/CP, and 2H-WS_2/CP electrodes. (**A**) Time-voltage curves, (**B**) median voltage, and (**C**) energy efficiency at 100 μA c cm^{-2}.

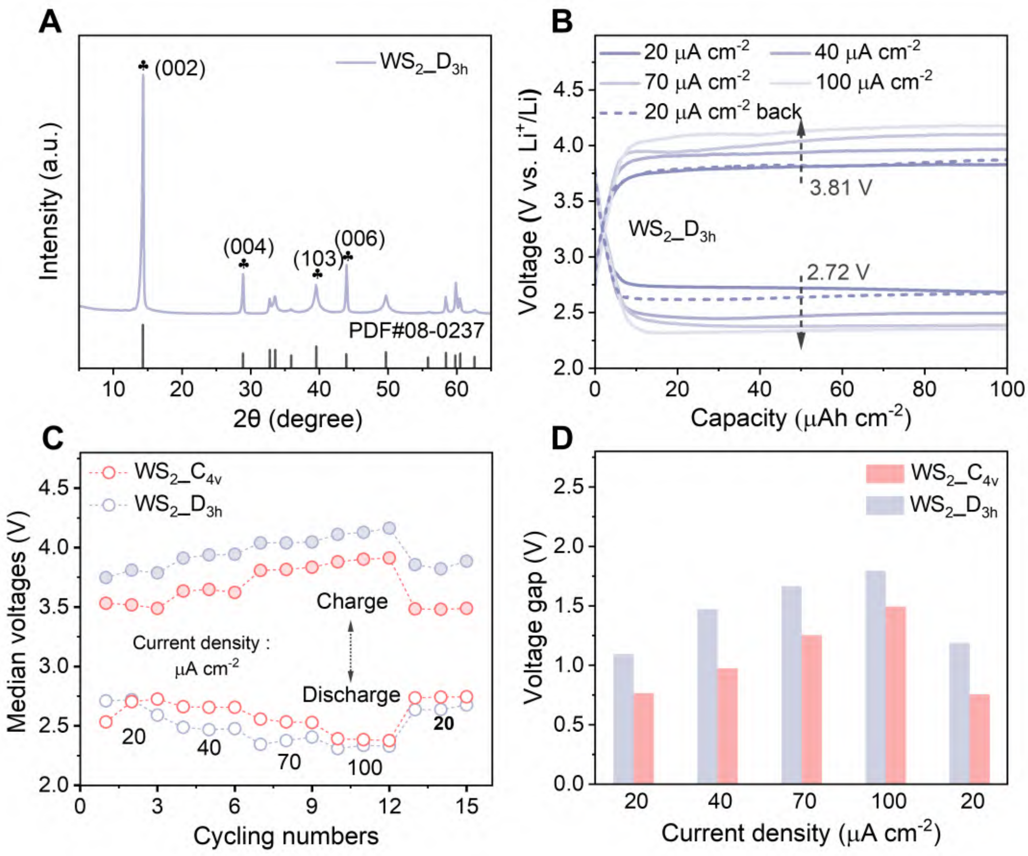

Fig. S54. Electrochemical performance of $WS_2\_D_{3h}$. (**A**) XRD patterns, (**B**) GDC profiles, (**C**) median voltages and (**D**) voltage gaps of $WS_2\_D_{3h}$.

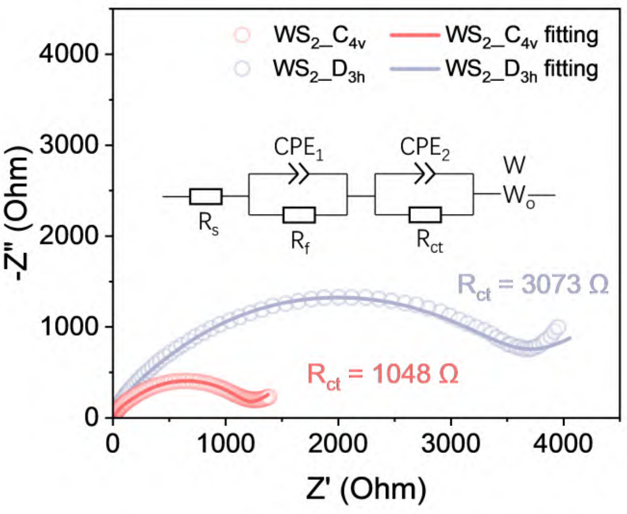

Fig. S55. Nyquist plots of $WS_2\_C_{4v}$ and $WS_2\_D_{3h}$ cells. The insets are the equivalent circuit models.

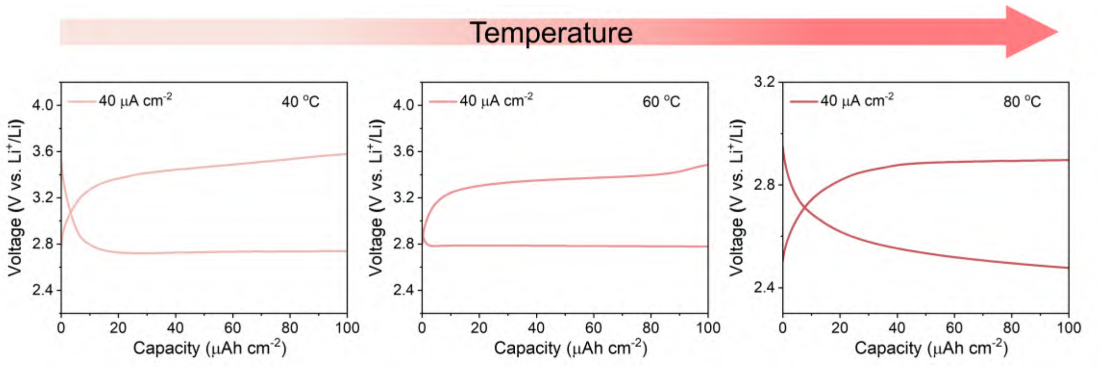

Fig. S56. High-temperature performance. The GDC profiles for coin cell at different temperature.

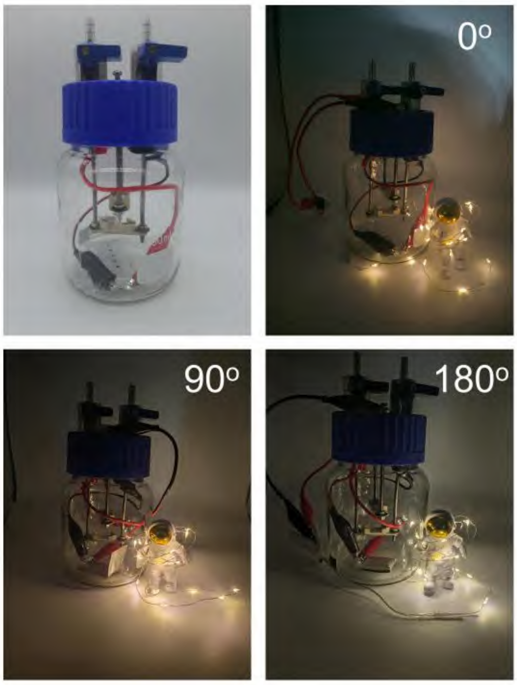

Fig. S57. Demonstration of flexible Li-CO_2 batteries. LED strips lit up by the flexible Li-CO_2 batteries under different deformations.

Table S1. Convergence tests. Convergence tests for 2H-MoS_2 and 2H-WS_2 zigzag edges.

	ENCUT	Energy (eV)	Average energy (eV/atom)	Relative energy (eV/atom)
2H-MoS_2	400	-426.687	-7.111	0.000
	450	-426.340	-7.106	0.006
	500	-426.641	-7.111	-0.005
	550	-426.599	-7.110	0.001
2H-WS_2	400	-463.039	-7.717	0.000
	450	-462.529	-7.709	0.009
	500	-462.836	-7.714	-0.005
	550	-462.976	-7.716	-0.002

	KPOINTS	Energy (eV)	Average energy (eV/atom)	Relative energy (eV/atom)
2H-MoS_2	111	-426.807	-7.113	0.000
	121	-426.340	-7.106	0.008
	131	-426.396	-7.107	-0.001
	141	-426.370	-7.106	0.000
2H-WS_2	111	-463.011	-7.717	0.000
	211	-462.529	-7.709	0.008
	311	-462.652	-7.711	-0.002
	411	-462.667	-7.711	0.000

	EDIFFG	Energy (eV)	Average energy (eV/atom)	Relative energy (eV/atom)
2H-MoS_2	-0.08	-426.370	-7.106	0.000
	-0.05	-426.340	-7.106	0.000
	-0.03	-426.377	-7.106	-0.001
	-0.02	-426.380	-7.106	0.000
2H-WS_2	-0.08	-462.645	-7.711	0.000
	-0.05	-462.529	-7.709	0.002
	-0.03	-462.649	-7.711	-0.002
	-0.02	-462.650	-7.711	0.000

Table S2. Rate performance summary. Rate performance of CP, 1T-SnS_2/CP, 1T′-ReS_2/CP, 2H-MoS_2/CP, and 2H-WS_2/CP.

Electrodes	Rate performance<br>Median charge voltage/<br>median discharge voltage	Voltage gap/<br>energy efficiency	Current densities
CP	4.38, 4.48, 4.66 V/<br>2.49, 2.47, 2.37 V	1.89, 2.01, 2.28 V/<br>57.43, 55.98, 52.12%	20, 40, 70<br>μA cm^{-2}
1T-SnS_2/CP	4.32, 4.44, 4.60 V/<br>2.56, 2.49, 2.38 V	1.76, 1.95, 2.22 V/<br>59.97, 56.76, 52.68 %	20, 40, 70<br>μA cm^{-2}
1T′-ReS_2/CP	3.87, 3.93, 4.05, 4.25, 3.81 V/<br>2.72, 2.65, 2.57, 2.47, 2.75 V	1.14, 1.29, 1.49, 1.78, 1.06 V/<br>71.94, 69.71, 66.20, 61.43, 74.81%	20, 40, 70, 100, 20<br>μA cm^{-2}
2H-MoS_2/CP	3.66, 3.75, 3.96, 4.04, 3.60 V/<br>2.70, 2.67, 2.60, 2.52, 2.81 V	0.96, 1.08, 1.36, 1.52, 0.79 V/<br>75.73, 73.66, 68.89, 65.39, 79.40%	20, 40, 70, 100, 20<br>μA cm^{-2}
2H-WS_2/CP	3.49, 3.63, 3.81, 3.88, 3.48 V/<br>2.73, 2.66, 2.56, 2.39, 2.74 V	0.76, 0.97, 1.25, 1.49, 0.75 V/<br>81.39, 76.76, 71.24, 65.84, 82.74%	20, 40, 70, 100, 20<br>μA cm^{-2}

Table S3. Comparison of voltage gaps with literature. Comparison of the voltage gaps and charge voltage for Li-CO_2 batteries.

Cathode catalysts	Charge voltage (V)	Voltage gap (V)	Reference
**1.1 Carbon based catalysts**			
graphene	4.3	1.55	(44)
CNT	4.26	1.59	(45)
B-NCNT	4.08	1.33	(46)
N-CNTs@Ti	4.25	1.65	(47)
CM/CNTs	4.4	1.76	(48)
**1.2 Single atom catalysts**			
Fe-ISA/N,S-HG	3.95	1.17	(49)
SA Ru-Co_3O_4/CC	3.8	1.05	(50)
Cr-NG	3.9	1.1	(51)
0.2Cu–Co_4N@CC	4.25	1.32	(52)
Cu/NCNF	4.09	1.29	(53)
TeAC@NCNS	4.1	1.4	(54)
**1.3 Transition metal oxides catalysts**			
NiO NFs/CNTs	4.2	1.5	(55)
MnO@NC-rGO	3.95	0.88	(56)
IrO_2/MnO_2	4	1.4	(57)
α-MnO_2/CNT	3.98	1.35	(58)
MnO@NMCNFs	4.25	1.57	(59)
$Co_{0.1}Ni_{0.9}O_x$/CNT	4.24	1.56	(67)
**1.4 Nobel metal catalysts**			
Ru	3.6	1.1	(68)
Ir/CNFs	4.14	1.38	(60)
Ru/NS-G	4.04	1.13	(61)
Ru-GDYS	3.61	1.11	(62)
PdCu/N-CNF	4.1	1.2	(63)
Ir-Te NWs	4.4	1.7	(64)
**1.5 Transition metal sulfide catalysts**			
MoS_2-NS	3.87	1.02	(69)
NiS_2/FeS_2-NSGA	3.9	1.1	(70)
Commercial WS_2	3.81	1.09	This work
WS_2/CP	3.49	0.76	This work

Table S4. Voltage gap reduction by symmetry breaking. Comparison of voltage gaps between metal sulfide catalysts with and without symmetry breaking.

Cathode catalysts	Voltage gap (V)	Voltage gap (V)	Reference
V-MoS_2/Co_9S_8@CP	1.29	**0.68**	(33)
NS_V-ReS_2(5)/CP	1.28	**0.66**	(20)
CoS_2	0.89	**0.52**	(8)
Vs-Co_2CuS_4	1.14	**0.73**	(65)
S_V-CoS	0.88	**0.43**	(66)

Note: The voltage gaps for metal sulfide catalysts with symmetry breaking are indicated in boldface type.

REFERENCES

1. L. Liu, S. Shen, J. Li, N. Zhao, X. Yin, H. Zhao, W. Yu, Y. Su, B. Y. Xia, S. Ding, *d*-electrons of platinum alloy steering CO pathway for low-charge potential Li-CO_2 batteries. *Angew. Chem. Int. Ed.* **64**, e202415728 (2025).

2. J. Zhou, Z. Xu, K. Cui, J.-A. Yin, H.-C. Chen, Y. Wang, F. Liu, T. Wang, F. Hao, Y. Xiong, C. Wang, Y. Ma, P. Lu, J. Yin, L. Guo, X. Meng, C. Ye, H. M. Chen, Y. Zhu, J. Lu, Z. Fan, Theory-guided design of unconventional phase metal heteronanostructures for higher-rate stable Li-CO_2 and Li-air batteries. *Angew. Chem. Int. Ed.* **64**, e202416947 (2025).

3. F. Zhang, W. Zhang, J. A. Yuwono, D. Wexler, Y. Fan, J. Zou, G. Liang, L. Sun, Z. Guo, Catalytic role of in-situ formed C-N species for enhanced Li_2CO_3 decomposition. *Nat. Commun.* **15**, 3393 (2024).

4. Y. Liu, T. Liu, X. Wang, J. Zhang, X. Zhai, T. Wei, Q. Shi, C. Lu, H. Yan, Y. Xia, W. Cheng, M. Zhou, Subsurface electron trap enabled long-cycling oxalate-based Li-CO_2 battery. *Adv. Mater.* **37**, e2507871 (2025).

5. J. Zhang, P. Shen, Y. Liu, T. Wei, X. Zhai, B. Zhu, J. Zeng, K. Xu, M. Zhou, Dynamic Ni-O bonding induced by orbital degeneracy breaking for efficient Li_2CO_3 decomposition. *Adv. Mater.* **38**, e17957 (2025).

6. X. Sun, X. Mu, W. Zheng, L. Wang, S. Yang, C. Sheng, H. Pan, W. Li, C.-H. Li, P. He, H. Zhou, Binuclear Cu complex catalysis enabling Li-CO_2 battery with a high discharge voltage above 3.0 V. *Nat.Commun.* **14**, 536 (2023).

7. C. Guo, F. Zhang, X. Han, L. Zhang, Q. Hou, L. Gong, J. Wang, Z. Xia, J. Hao, K. Xie, Intrinsic descriptor guided noble metal cathode design for Li-CO_2 battery. *Adv. Mater.* **35**, e2302325 (2023).

8. Y. Liu, Z. Zhang, J. Tan, B. Chen, B. Lu, R. Mao, B. Liu, D. Wang, G. Zhou, H.-M. Cheng, Deciphering the contributing motifs of reconstructed cobalt (II) sulfides catalysts in Li-CO_2 batteries. *Nat.Commun.* **15**, 2167 (2024).

9. K. Zhu, X. Li, J. Choi, C. Choi, S. Hong, X. Tan, T.-S. Wu, Y.-L. Soo, L. Hao, A. W. Robertson, Y. Jung, Z. Sun, Single-atom cadmium-N_4 Sites for rechargeable Li–CO_2 batteries with high capacity and ultra-long lifetime. *Adv. Funct. Mater.* **33**, 2213841 (2023).

10. Y. Liu, X. Wu, H. Qu, G. Lu, Y. Chen, B. Lu, Y. Song, G. Zhou, H.-M. Cheng, Regulating the local spin states in spinel oxides to promote the activity of Li-CO_2 batteries. *Adv. Mater.* **37**, e2411652 (2025).

11. B. Lu, X. Wu, M. Zhang, X. Xiao, B. Chen, Y. Liu, R. Mao, Y. Song, X.-X. Zeng, J. Yang, G. Zhou, Steering the orbital hybridization to boost the redox kinetics for efficient Li-CO_2 batteries. *J. Am. Chem. Soc.* **146**, 20814–20822 (2024).

12. Y. Chen, J. Li, B. Lu, Y. Liu, R. Mao, Y. Song, H. Li, X. Yu, Y. Gao, Q. Peng, X. Qi, G. Zhou, Activated Co in thiospinel boosting Li_2CO_3 decomposition in Li-CO_2 batteries. *Adv. Mater.* **36**, e2406856 (2024).

13. B. Chen, D. Wang, B. Zhang, X. Zhong, Y. Liu, J. Sheng, Q. Zhang, X. Zou, G. Zhou, H.-M. Cheng, Engineering the active sites of graphene catalyst: From CO_2 activation to activate Li-CO_2 batteries. *ACS Nano* **15**, 9841–9850 (2021).

14. H. Chen, X. Li, Z. Liu, Y. Xu, Y. Yan, P. Li, K. Chang, X. Huang, J. He, T. Wang, Ferrocene-based nickel metal-organic framework nanosheets as efficient, long-cycle cathode catalyst for Li-CO_2 battery. *Adv. Funct. Mater.* **35**, 2412387 (2025).

15. Y. Shi, B. Wei, D. Legut, S. Du, J. S. Francisco, R. Zhang, Highly stable single-atom modified MXenes as cathode-active bifunctional catalysts in Li–CO_2 battery. *Adv. Funct. Mater.* **32**, 2210218 (2022).

16. M. Chhowalla, H. S. Shin, G. Eda, L.-J. Li, K. P. Loh, H. Zhang, The chemistry of two-dimensional layered transition metal dichalcogenide nanosheets. *Nat. Chem.* **5**, 263–275 (2013).

17. C. Zhang, Y. Luo, J. Tan, Q. Yu, F. Yang, Z. Zhang, L. Yang, H.-M. Cheng, B. Liu, High-throughput production of cheap mineral-based two-dimensional electrocatalysts for high-current-density hydrogen evolution. *Nat. Commun.* **11**, 3724 (2020).

18. B. Chen, D. Chao, E. Liu, M. Jaroniec, N. Zhao, S.-Z. Qiao, Transition metal dichalcogenides for alkali metal ion batteries: Engineering strategies at the atomic level. *Energy Environ. Sci.* **13**, 1096–1131 (2020).

19. A. Ahmadiparidari, R. E. Warburton, L. Majidi, M. Asadi, A. Chamaani, J. R. Jokisaari, S. Rastegar, Z. Hemmat, B. Sayahpour, R. S. Assary, B. Narayanan, P. Abbasi, P. C. Redfern, A. Ngo, M. Vörös, J. Greeley, R. Klie, L. A. Curtiss, A. Salehi-Khojin, A long-cycle-life lithium-CO_2 battery with carbon neutrality. *Adv. Mater.* **31**, e1902518 (2019).

20. B. Chen, D. Wang, J. Tan, Y. Liu, M. Jiao, B. Liu, N. Zhao, X. Zou, G. Zhou, H.-M. Cheng, Designing electrophilic and nucleophilic dual centers in the ReS_2 plane toward efficient bifunctional catalysts for Li-CO_2 batteries. *J. Am. Chem. Soc.* **144**, 3106–3116 (2022).

21. T. F. Jaramillo, K. P. Jørgensen, J. Bonde, J. H. Nielsen, S. Horch, I. Chorkendorff, Identification of active edge sites for electrochemical H_2 evolution from MoS_2 nanocatalysts. *Science* **317**, 100–102 (2007).

22. H. Wang, Q. Zhang, H. Yao, Z. Liang, H.-W. Lee, P.-C. Hsu, G. Zheng, Y. Cui, High electrochemical selectivity of edge versus terrace sites in two-dimensional layered MoS_2 materials. *Nano Lett.* **14**, 7138–7144 (2014).

23. H. Rostami, R. Asgari, F. Guinea, Edge modes in zigzag and armchair ribbons of monolayer MoS_2. *J. Phys. Condens. Matter* **28**, 495001 (2016).

24. H. Pan, Y.-W. Zhang, Edge-dependent structural, electronic and magnetic properties of MoS_2 nanoribbons. *J. Mater. Chem.* **22**, 7280–7290 (2012).

25. H. Schweiger, P. Raybaud, G. Kresse, H. Toulhoat, Shape and edge sites modifications of MoS_2 catalytic nanoparticles induced by working conditions: A theoretical study. *J. Catal.* **207**, 76–87 (2002).

26. S. Tongay, S. S. Varnoosfaderani, B. R. Appleton, J. Wu, A. F. Hebard, Magnetic properties of MoS_2: Existence of ferromagnetism. *Appl. Phys. Lett.* **101**, 123105 (2012).

27. N. Huo, Y. Li, J. Kang, R. Li, Q. Xia, J. Li, Edge-states ferromagnetism of WS_2 nanosheets. *Appl. Phys. Lett.* **104**, 202406 (2014).

28. L. Pang, Z. Zhao, T. Liu, G. Wang, S. Dong, Z. Peng, Unlock CO_2 reduction reaction pathways in aprotic Li-CO_2 batteries with in situ isotope-labeled spectroscopy and theoretical calculations. *J. Am. Chem. Soc.* **146**, 17917–17923 (2024).

29. R. Zeng, Q. Gao, L. Xiao, W. Wang, Y. Gu, H. Huang, Y. Tan, D. Tang, S. Guo, Precise tuning of the *d*-band center of dual-atomic enzymes for catalytic therapy. *J. Am. Chem. Soc.* **146**, 10023–10031 (2024).

30. X. Sun, L. Sun, G. Li, Y. Tuo, C. Ye, J. Yang, J. Low, X. Yu, J. H. Bitter, Y. Lei, D. Wang, Y. Li, Phosphorus tailors the *d*-band center of copper atomic sites for efficient CO_2 photoreduction under visible-light irradiation. *Angew. Chem. Int. Ed.* **61**, e202207677 (2022).

31. Z. Huang, S. Hu, M. Sun, Y. Xu, S. Liu, R. Ren, L. Zhuang, T.-S. Chan, Z. Hu, T. Ding, J. Zhou, L. Liu, M. Wang, Y.-C. Huang, N. Tian, L. Bu, B. Huang, X. Huang, Implanting oxophilic metal in PtRu nanowires for hydrogen oxidation catalysis. *Nat. Commun.* **15**, 1097 (2024).

32. Z. Han, R. Gao, T. Wang, S. Tao, Y. Jia, Z. Lao, M. Zhang, J. Zhou, C. Li, Z. Piao, X. Zhang, G. Zhou, Machine-learning-assisted design of a binary descriptor to decipher electronic and structural effects on sulfur reduction kinetics. *Nat. Catal.* **6**, 1073–1086 (2023).

33. B. Lu, B. Chen, D. Wang, C. Li, R. Gao, Y. Liu, R. Mao, J. Yang, G. Zhou, Engineering the interfacial orientation of MoS_2/Co_9S_8 bidirectional catalysts with highly exposed active sites for reversible Li-CO_2 batteries. *Proc. Natl. Acad. Sci. U.S.A.* **120**, e2216933120 (2023).

34. M. Luo, F. Wei, C. Jiang, J. H. Zhang, W. Xu, J. D. Liu, B. J. Ye, H. J. Zhang, Effect of free-volume holes on mechanical properties of carbon-fiber-reinforced polymers (CFRPs) studied by positron annihilation age-momentum correlation spectroscopy. *Polymer* **320**, 128076 (2025).

35. A. Siegle, H. Stoll, P. Castellaz, J. Major, H. Schneider, A. Seeger, Two-dimensional analysis of positron age-momentum correlation (AMOC) data. *Appl. Surf. Sci.* **116**, 140–144 (1997).

36. W. Zhan, X. Zhai, Y. Li, M. Wang, H. Wang, L. Wu, X. Tang, H. Zhang, B. Ye, K. Tang, G. Wang, M. Zhou, Regulating local atomic environment around vacancies for efficient hydrogen evolution. *ACS Nano* **18**, 10312–10323 (2024).

37. A. R. Woldu, P. Talebi, A. G. Yohannes, J. Xu, X.-D. Wu, S. Siahrostami, L. Hu, X.-C. Huang, Insights into electrochemical CO_2 reduction on SnS_2: Main product switch from hydrogen to formate by pulsed potential electrolysis. *Angew. Chem. Int. Ed.* **62**, e202301621 (2023).

38. Q.-Q. Pang, Z.-L. Niu, S.-S. Yi, S. Zhang, Z.-Y. Liu, X.-Z. Yue, Hydrogen-etched bifunctional sulfur-defect-rich ReS_2/CC electrocatalyst for highly efficient HER and OER. *Small* **16**, e2003007 (2020).

39. G. Kresse, J. Furthmüller, Efficient iterative schemes for ab initio total-energy calculations using a plane-wave basis set. *Phys. Rev. B* **54**, 11169–11186 (1996).

40. G. Kresse, D. Joubert, From ultrasoft pseudopotentials to the projector augmented-wave method. *Phys. Rev. B.* **59**, 1758–1775 (1999).

41. J. P. Perdew, K. Burke, M. Ernzerhof, Generalized gradient approximation made simple. *Phys. Rev. Lett.* **77**, 3865–3868 (1996).

42. S. Grimme, J. Antony, S. Ehrlich, H. Krieg, A consistent and accurate ab initio parametrization of density functional dispersion correction (DFT-D) for the 94 elements H-Pu. *J. Chem. Phys.* **132**, 154104–154100 (2010).

43. J. Zhang, H. Zhang, H. Ye, Y. Zheng, Free-end adaptive nudged elastic band method for locating transition states in minimum energy path calculation. *J. Chem. Phys.* **145**, 094104 (2016).

44. Z. Zhang, Q. Zhang, Y. Chen, J. Bao, X. Zhou, Z. Xie, J. Wei, Z. Zhou, The first introduction of graphene to rechargeable Li-CO_2 batteries. *Angew. Chem. Int. Ed.* **54**, 6550–6553 (2015).

45. X. Zhang, Q. Zhang, Z. Zhang, Y. Chen, Z. Xie, J. Wei, Z. Zhou, Rechargeable Li-CO_2 batteries with carbon nanotubes as air cathodes. *Chem. Commun.* **51**, 14636–14639 (2015).

46. X. Li, J. Zhou, J. Zhang, M. Li, X. Bi, T. Liu, T. He, J. Cheng, F. Zhang, Y. Li, X. Mu, J. Lu, B. Wang, Bamboo-like nitrogen-doped carbon nanotube forests as durable metal-free catalysts for self-powered flexible Li-CO_2 batteries. *Adv. Mater.* **31**, e1903852 (2019).

47. Y. Li, J. Zhou, T. Zhang, T. Wang, X. Li, Y. Jia, J. Cheng, Q. Guan, E. Liu, H. Peng, B. Wang, Highly surface-wrinkled and N-doped CNTs anchored on metal wire: A novel fiber-shaped cathode toward high-performance flexible Li–CO_2 batteries. *Adv. Funct. Mater.* **29**, 1808117 (2019).

48. X. Ji, Y. Liu, Z. Zhang, J. Cui, Y. Fan, Y. Qiao, Porous carbon foam with carbon nanotubes as cathode for Li−CO_2 batteries. *Chem. A Eur. J.* **30**, e202303319 (2024).

49. C. Hu, L. Gong, Y. Xiao, Y. Yuan, N. M. Bedford, Z. Xia, L. Ma, T. Wu, Y. Lin, J. W. Connell, R. Shahbazian-Yassar, J. Lu, K. Amine, L. Dai, High-performance, long-life, rechargeable Li-CO_2 batteries based on a 3D holey graphene cathode implanted with single iron atoms. *Adv. Mater.* **32**, e1907436 (2020).

50. Z. Lian, Y. Lu, C. Wang, X. Zhu, S. Ma, Z. Li, Q. Liu, S. Zang, Single-atom Ru implanted on Co_3O_4 nanosheets as efficient dual-catalyst for Li-CO_2 batteries. *Adv. Sci.* **8**, e2102550 (2021).

51. Y. Liu, S. Zhao, D. Wang, B. Chen, Z. Zhang, J. Sheng, X. Zhong, X. Zou, S. P. Jiang, G. Zhou, H.-M. Cheng, Toward an understanding of the reversible Li-CO_2 batteries over metal-N_4-functionalized graphene electrocatalysts. *ACS Nano* **16**, 1523–1532 (2022).

52. X. Ma, W. Zhao, Q. Deng, X. Fu, L. Wu, W. Yan, Y. Yang, In-situ construction of Cu-Co_4N@CC hierarchical binder-free cathode for advanced and flexible Li-CO_2 batteries: Electron structure and mass transfer modulation. *J. Power Sources* **535**, 231446 (2022).

53. Y. Xu, H. Gong, L. Song, Y. Kong, C. Jiang, H. Xue, P. Li, X. Huang, J. He, T. Wang, A highly efficient and free-standing copper single atoms anchored nitrogen-doped carbon nanofiber cathode toward reliable Li-CO_2 batteries. *Mater. Today Energy* **25**, 100967 (2022).

54. K. Wang, D. Liu, L. Liu, X. Li, H. Wu, Z. Sun, M. Li, A. S. Vasenko, S. Ding, F. Wang, C. Xiao, Isolated metalloid tellurium atomic cluster on nitrogen-doped carbon nanosheet for high-capacity rechargeable lithium-CO_2 battery. *Adv. Sci.* **10**, e2205959 (2023).

55. S. Lu, Y. Shang, S. Ma, Y. Lu, Q. C. Liu, Z. J. Li, Porous NiO nanofibers as an efficient electrocatalyst towards long cycling life rechargeable Li-CO_2 batteries. *Electrochim. Acta* **319**, 958–965 (2019).

56. S. Li, Y. Liu, J. Zhou, S. Hong, Y. Dong, J. Wang, X. Gao, P. Qi, Y. Han, B. Wang, Monodispersed MnO nanoparticles in graphene-an interconnected N-doped 3D carbon framework as a highly efficient gas cathode in Li-CO_2 batteries. *Energy Environ. Sci.* **12**, 1046–1054 (2019).

57. Y. Mao, C. Tang, Z. Tang, J. Xie, Z. Chen, J. Tu, G. Cao, X. Zhao, Long-life Li-CO_2 cells with ultrafine IrO_2-decorated few-layered δ-MnO_2 enabling amorphous Li_2CO_3 growth. *Energy Storage Mater.* **18**, 405–413 (2019).

58. D. Lei, S. Ma, Y. Lu, Q. Liu, Z. Li, High-performance Li-CO_2 batteries with α-MnO_2/CNT cathodes. *J. Electron. Mater.* **48**, 4653–4659 (2019).

59. S. Li, Y. Liu, X. Gao, J. Wang, J. Zhou, L. Wang, B. Wang, Improving areal capacity of flexible Li-CO_2 batteries by constructing a freestanding cathode with monodispersed MnO nanoparticles in N-doped mesoporous carbon nanofibers. *J. Mater. Chem. A* **8**, 10354–10362 (2020).

60. C. Wang, Q. Zhang, X. Zhang, X.-G. Wang, Z. Xie, Z. Zhou, Fabricating Ir/C nanofiber networks as free-standing air cathodes for rechargeable Li-CO_2 batteries. *Small* **14**, e1800641 (2018).

61. Y. Qiao, J. Wu, J. Zhao, Q. Li, P. Zhang, C. Hao, X. Liu, S. Yang, Y. Liu, Synergistic effect of bifunctional catalytic sites and defect engineering for high-performance Li-CO_2 batteries. *Energy Storage Mater.* **27**, 133–139 (2020).

62. Y. Ma, H. Qu, W. Wang, Z. Guo, Y. Yu, F. Liu, B. Yu, M. Tian, Z. Li, B. Li, L. Wang, Graphdiyne scaffold anchored highly dispersed ruthenium nanoparticles as an efficient cathode catalyst for rechargeable Li-CO_2 battery. *Chin. Chem. Lett.* **35**, 108352 (2024).

63. H. Gong, X. Yu, Y. Xu, B. Gao, H. Xue, X. Fan, H. Guo, T. Wang, J. He, Long-life reversible Li-CO_2 batteries with optimized Li_2CO_3 flakes as discharge products on palladium-copper nanoparticles. *Inorg. Chem. Front.* **9**, 1533–1540 (2022).

64. Y. Zhai, H. Tong, J. Deng, G. Li, Y. Hou, R. Zhang, J. Wang, Y. Lu, K. Liang, P. Chen, F. Dang, B. Kong, Super-assembled atomic Ir catalysts on Te substrates with synergistic catalytic capability for Li-CO_2 batteries. *Energy Storage Mater.* **43**, 391–401 (2021).

65. B. Lu, X. Wu, X. Xiao, B. Chen, W. Zeng, Y. Liu, Z. Lao, X.-X. Zeng, G. Zhou, J. Yang, Energy band engineering guided design of bidirectional catalyst for reversible Li-CO_2 batteries. *Adv. Mater.* **36**, e2308889 (2024).

66. R. Mao, Y. Liu, P. Shu, B. Lu, B. Chen, Y. Chen, Y. Song, Y. Jia, Z. Zheng, Q. Peng, G. Zhou, Tailoring Li_2CO_3 configuration and orbital structure of CoS to improve catalytic activity and stability for Li-CO_2 batteries. *EcoMat* **6**, e12449 (2024).

67. X. Xiao, Z. Zhang, W. Yu, W. Shang, Y. Ma, X. Zhu, P. Tan, Ultrafine Co-Doped NiO nanoparticles decorated on carbon nanotubes improving the electrochemical performance and cycling stability of Li-CO_2 batteries. *ACS Appl. Energy Mater.* **4**, 11858–11866 (2021).

68. Y. Qiao, J. Yi, S. Wu, Y. Liu, S. Yang, P. He, H. Zhou, Li-CO_2 electrochemistry: A new strategy for CO_2 fixation and energy storage. *Joule* **1**, 359–370 (2017).

69. R. Pipes, J. He, A. Bhargav, A. Manthiram, Efficient Li-CO_2 batteries with molybdenum disulfide nanosheets on carbon nanotubes as a catalyst. *ACS Appl. Energy Mater.* **2**, 8685–8694 (2019).

70. Y. Jin, Y. Liu, L. Song, J. Yu, K. Li, M. Zhang, J. Wang, Interfacial engineering in hollow NiS_2/FeS_2-NSGA heterostructures with efficient catalytic activity for advanced Li-CO_2 battery. *Chem. Eng. J.* **430**, 133029 (2022).
